# π‐Electron Donation at the Sulfoximidoyl Nitrogen Atom

**DOI:** 10.1002/anie.202510744

**Published:** 2025-06-23

**Authors:** Benjamin J. Statham, Florian F. Mulks, Carsten Bolm

**Affiliations:** ^1^ Institute of Organic Chemistry RWTH Aachen University Landoltweg 1 52074 Aachen Germany

**Keywords:** +M‐effect, DFT, *N*‐functionalized sulfoximines, Properties, Reactivity

## Abstract

Knowledge of the physicochemical properties and the reactivity of functional groups is invaluable in organic chemistry. Although substantial understanding of properties/reactivities exists for standard functional groups, moieties which have entered the chemist's toolbox more recently are often not as well characterized. Sulfoximines constitute such a class of recently strongly promoted compounds, which nowadays have emerged as relevant molecules in an industrial context and in academic research. However, theoretical studies on the properties of the sulfoximidoyl group remain sparse. The aim of the present work was to investigate the interactions of the sulfoximidoyl nitrogen lone pairs with adjacent π‐acceptor orbitals. Density functional theory‐based (DFT‐based) methods were employed throughout the investigation to quantify the strength of these interactions and to characterize their influence on the properties/reactivities of *N*‐functionalized sulfoximines. Although sulfoximines are commonly characterized as electron‐withdrawing, our analysis suggests a strong positive mesomeric effect of the sulfoximidoyl group at nitrogen. This effect increases the basicity and nucleophilicity of conjugated substituents at the sulfoximidoyl nitrogen atom.

## Graphical abstract

Density functional theory (DFT)‐based evidence for a strong positive mesomeric effect (+M‐effect) of the sulfoximidoyl group at N is presented. Our findings contrast with common characterizations of the sulfoximidoyl group as an exclusively electron‐withdrawing moiety. The implications for reactivity are discussed, and it is demonstrated that the +M‐effect explains experimental observations in the field of sulfoximine chemistry.

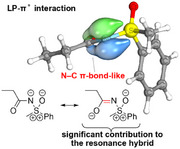

## Introduction

Sulfoximines, first discovered by Bentley and coworkers in 1950, have been established as interesting candidates for drugs and agrochemicals over the last 70 years.^[^ ^1^, ^2^, ^3^, ^4^, ^5^, ^6^, ^7^, ^8^, ^9^, ^10^, ^11^, ^12^ ^]^ Besides their bioactivity, sulfoximines possess a range of properties that make them valuable in this context, including good solubility in water, hydrogen bond donor and acceptor capabilities, and an additional point of substitution at the sulfoximidoyl nitrogen atom compared to the related sulfones.^[^ ^5^, ^6^, ^7^, ^8^ ^]^ Variations of the substituent at nitrogen significantly affect the acidity of aliphatic units in alpha position to the sulfoximidoyl sulfur atom and can be utilized to fine‐tune compound properties.^[^ ^5^, ^6^, ^7^, ^8^, ^12^, ^13^, ^14^, ^15^, ^16^ ^]^ Several strategies have been established to introduce substituents at the sulfoximidoyl nitrogen atom, encompassing transformations such as acylations, arylations, and alkynylations, to name a few.^[^ ^17^, ^18^, ^19^, ^20^, ^21^, ^22^, ^23^, ^24^, ^25^, ^26^, ^27^, ^28^, ^29^, ^30^, ^31^, ^32^, ^33^, ^34^, ^35^, ^36^, ^37^, ^38^, ^39^ ^]^ Such reactions not only provide access to an array of new compounds but also allow to synthesize heterocyclic sulfoximines in intramolecular ring closure reactions, which are of special interest for pharmaceutical and agrochemical research.^[^ ^40^, ^41^, ^42^, ^43^, ^44^, ^45^, ^46^, ^47^, ^48^ ^]^

Although much synthetic work has been conducted with the aim of introducing new functionalities at the sulfoximidoyl nitrogen atom or developing novel methodologies to achieve such functionalizations,^[^ ^17^, ^18^, ^19^, ^20^, ^21^, ^22^, ^23^, ^24^, ^25^, ^26^, ^27^, ^28^, ^29^, ^30^, ^31^, ^32^, ^33^, ^34^, ^35^, ^36^, ^37^, ^38^, ^39^ ^]^ little theoretical work on *N*‐substituted sulfoximines exists.^[^ ^49^, ^50^, ^51^ ^]^ Considering the increasing importance of the sulfoximidoyl group in industry and academic research, this lack of theoretical work is surprising since a robust knowledge of the reactivities/properties of functional groups can greatly assist the design of new transformations and the rationalization of reaction outcomes. With the current work, we contribute to filling this gap in the research literature.

**Figure 1:**
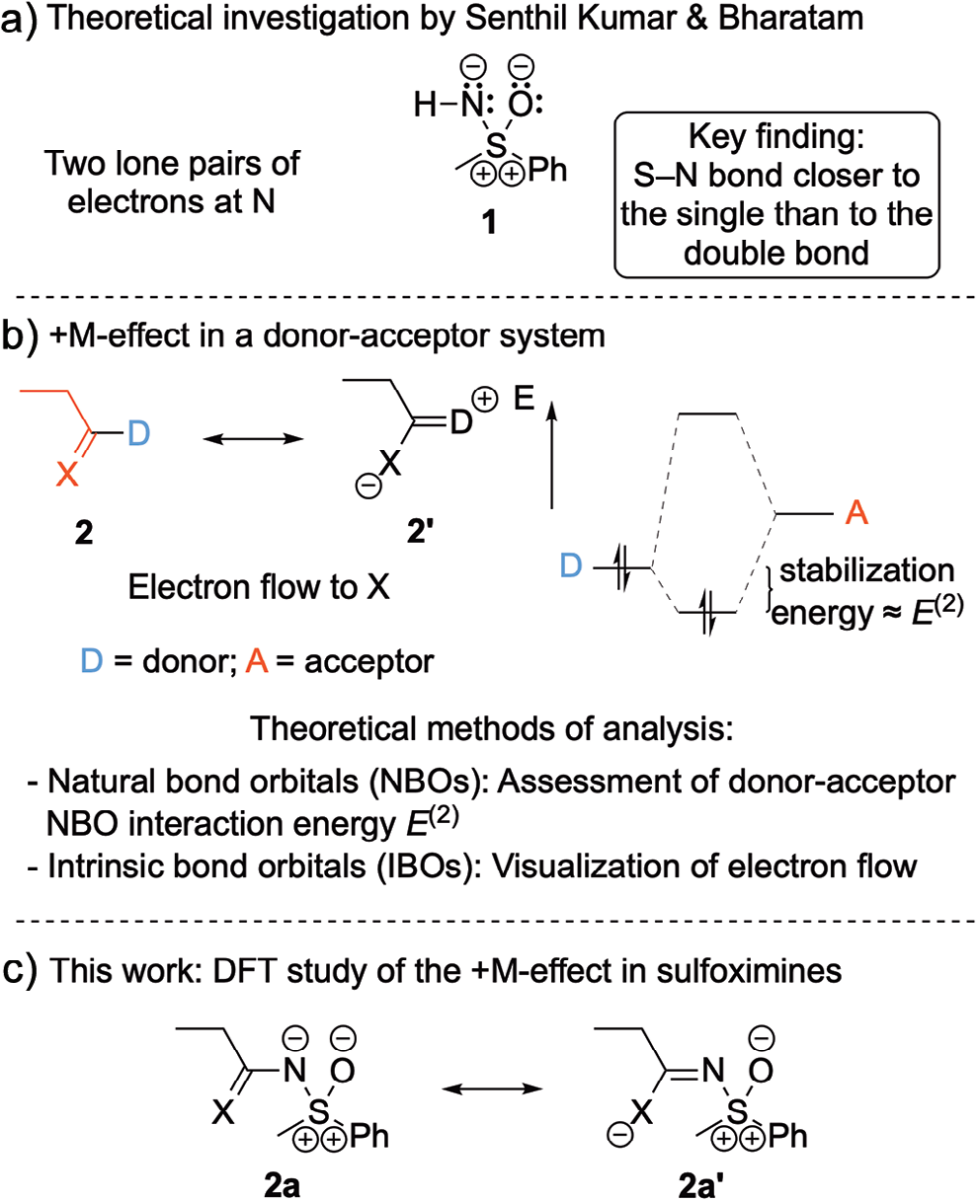
Summary of previous work and the present study. a) Previous theoretical investigations into the nature of the S─N bond in sulfoximines. b) +M‐effect in a general donor–acceptor system and theoretical methods of analysis. c) DFT investigation of the +M‐effect in sulfoximines (this work).

Theoretical studies by Senthil Kumar and Bharatam support that the S─N bonds in sulfoximines are closer to single than to double bonds.^[^ ^50^ ^]^ This is confirmed by related work from Stalke.^[^ ^52^, ^53^, ^54^ ^]^ Hence, for an *N*H‐sulfoximine such as *S*‐methyl‐*S*‐phenyl sulfoximine, a zwitterionic resonance form **1** can be written (Figure 1a). Although the depicted resonance form differs from common representations of sulfoximines in the literature, it represents the most accurate Lewis representation of the structure (see NBO analysis in the following text). We were interested in studying the interactions of the two sulfoximidoyl nitrogen lone pairs in resonance form **1** with acceptor orbitals adjacent to N; i.e., we wanted to know whether the sulfoximidoyl group exhibits a positive mesomeric effect (+M‐effect). For a donor–acceptor system such as **2**, such an interaction implies a significant contribution of resonance form **2′** to the resonance hybrid (see Figure 1b). The relevance of +M‐effects in π‐donor‐acceptor systems can be evaluated with natural bond orbitals (NBOs) and intrinsic bond orbitals (IBOs).^[^ ^55^, ^56^, ^57^, ^58^ ^]^ While NBO theory can be employed to assess the donor–acceptor NBO interaction energy, IBOs can be utilized to visualize the electron flow to the acceptor.^[^ ^55^, ^56^, ^57^, ^58^ ^]^ Contrary to previous reports that the sulfoximidoyl moiety is an exclusively electron‐withdrawing moiety, we describe herein for the first time the occurrence of π‐electron donation from the sulfoximidoyl nitrogen to adjacent, suitable π‐acceptors (Figure 1c). The sulfoximidoyl group is electron withdrawing at sulfur, but, as will be shown in the following text, exhibits electron donating properties at the sulfoximidoyl nitrogen atom. Density functional theory (DFT) based evidence for this effect is provided and implications for the reactivity of sulfoximines are discussed.

## Results and Discussion

**Figure 2:**
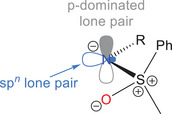
Lewis‐type, lone pair NBOs at the sulfoximidoyl nitrogen atom corresponding to a p‐dominated orbital (gray) and an sp*^n^*‐hybrid orbital (blue).

Our study commenced with NBO and IBO analyses of the donor–acceptor systems **2** shown in Figure 1b, comprising selected π‐acceptor substituted sulfoximines **2aa**–**2ac**, **2bc**, and **2cc** (D = ─N═S(═O)R_2_; X = CH_2_, NMe, and O). The results of these assessments are summarized in Table 1 and are compared with results obtained for a dimethylamino donor group **2da**–**2dc** (D = ─NMe_2_; X = CH_2_, NMe, and O). According to the NBO analysis of the acceptor‐substituted sulfoximines and in agreement with the computational work from Senthil Kumar and Bharatam, the best Lewis type description of the S─N bond is that of a single bond, in which two lone pairs are located on the sulfoximidoyl nitrogen atom. The two lone pairs are non‐equivalent and correspond to a p‐dominated orbital in one case and an sp*^n^*‐hybrid orbital (*n* = 1.4–1.7) in the other (Figure 2). The second order energies *E* ^(2)^ of the donor–acceptor NBO interactions indicate that the p‐type lone pair, unlike the sp*^n^*‐hybrid, interacts strongly with the π^*^‐acceptor orbitals on the substituents (see Table 1). This effect is of a similar or greater magnitude as compared to the lone pair‐π^*^‐interaction (LP‐π^*^‐interaction) involving the strong donor dimethylamino‐substituent.^[^ ^59^, ^60^ ^]^ The interaction of the nitrogen lone pair with the antibonding π^*^‐orbital of the acceptor constitutes the strongest determined interaction.

**Table 1:** Results of the NBO and IBO analysis of selected acceptor substituted sulfoximines, which are compared with the corresponding results obtained for the dimethylamino donor functionality.

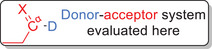						
Donor‐acceptor systems **2**	*E* ^(2)^ (kJ mol^−1^)^a)^	NBO LP occupancy ()^b)^	NBO π^*^ occupancy ()^c)^	IAO charge at N (e^−^)^d)^	IAO charge at C^α^ (e^−^)^d)^	IAO charge at X (e^−^)^d)^
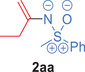	−168	1.65	0.16	1.61	0.11	0.05
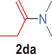	−118	1.76	0.19	1.73	0.13	0.06
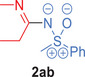	−193	1.65	0.21	1.60	0.15	0.04
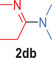	−114	1.78	0.21	1.72	0.15	0.05
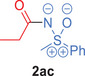	−270	1.63	0.26	1.57	0.23	0.03
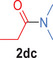	−267	1.67	0.32	1.60	0.30	0.04
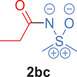	−281	1.63	0.27	1.57	0.25	0.03
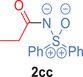	−262	1.62	0.25	1.56	0.23	0.03

^a)^ Second order estimate of the energy of the interaction between the nitrogen lone pair NBO and the π^*^‐NBO on the substituent. In the case of sulfoximines **2aa**–**2ac**, **2bc,** and **2cc**, the nitrogen lone pair involved in the interaction is the p‐dominated one.

^b)^ Occupancy of the nitrogen lone pair in the donor unit. For sulfoximines **2aa**‐**2ac**, **2bc**, and **2cc**, the occupancy of the p‐dominated lone pair is shown.

^c)^ Occupancy of the C═X π^*^‐orbital.

^d)^ IAO charges associated with the S─N π‐IBO.

The electronic occupations of the nitrogen lone pair and the antibonding π^*^‐orbital are listed in Table 1 and support the same conclusion. The occupation number of the p‐dominated lone pair shows the strongest deviation from a full occupation of 2.0, indicating that a significant portion of the electron density is redistributed to suitable, non‐Lewis type acceptor orbitals. The latter orbitals include the antibonding π^*^‐orbitals on the substituents which in turn are significantly electronically occupied (see Table 1). The occupation numbers found for the nitrogen lone pair and the antibonding π^*^‐orbital are of a similar magnitude to those found for the dimethylamino group.

**Figure 3:**
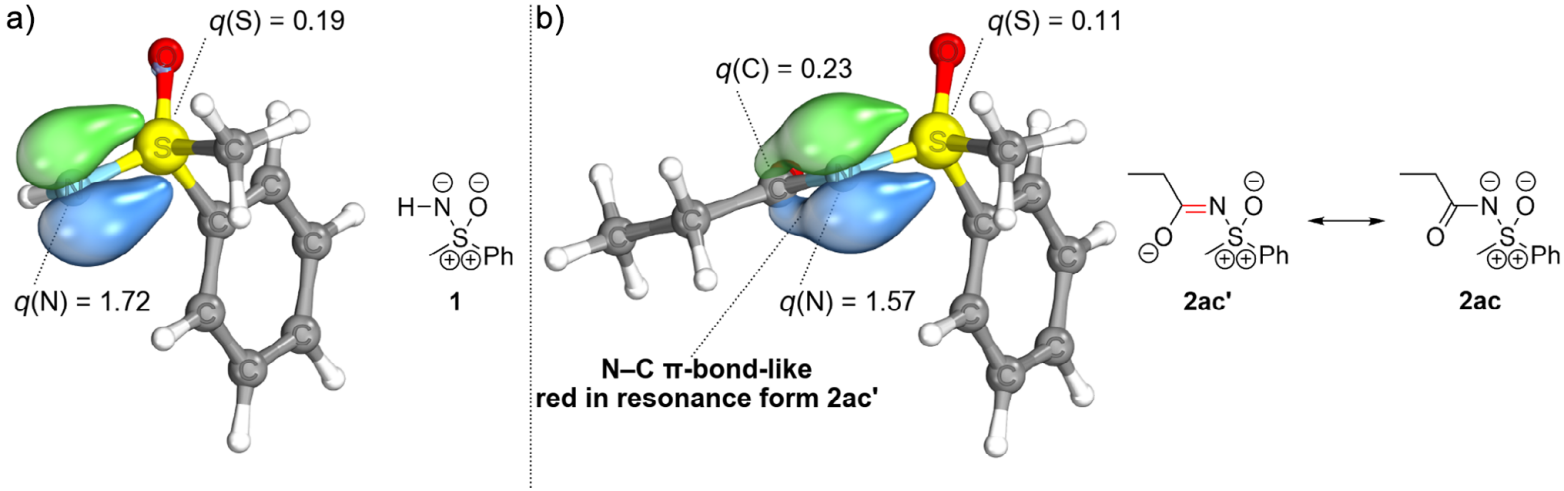
Intrinsic bond orbitals and their IAO partial charges. a) π‐Bond of the S─N unit in *S*‐methyl‐*S*‐phenyl sulfoximine. b) π‐Bond of the S─N unit in *N*‐propionyl‐*S*‐methyl‐*S*‐phenyl sulfoximine.

**Figure 4:**
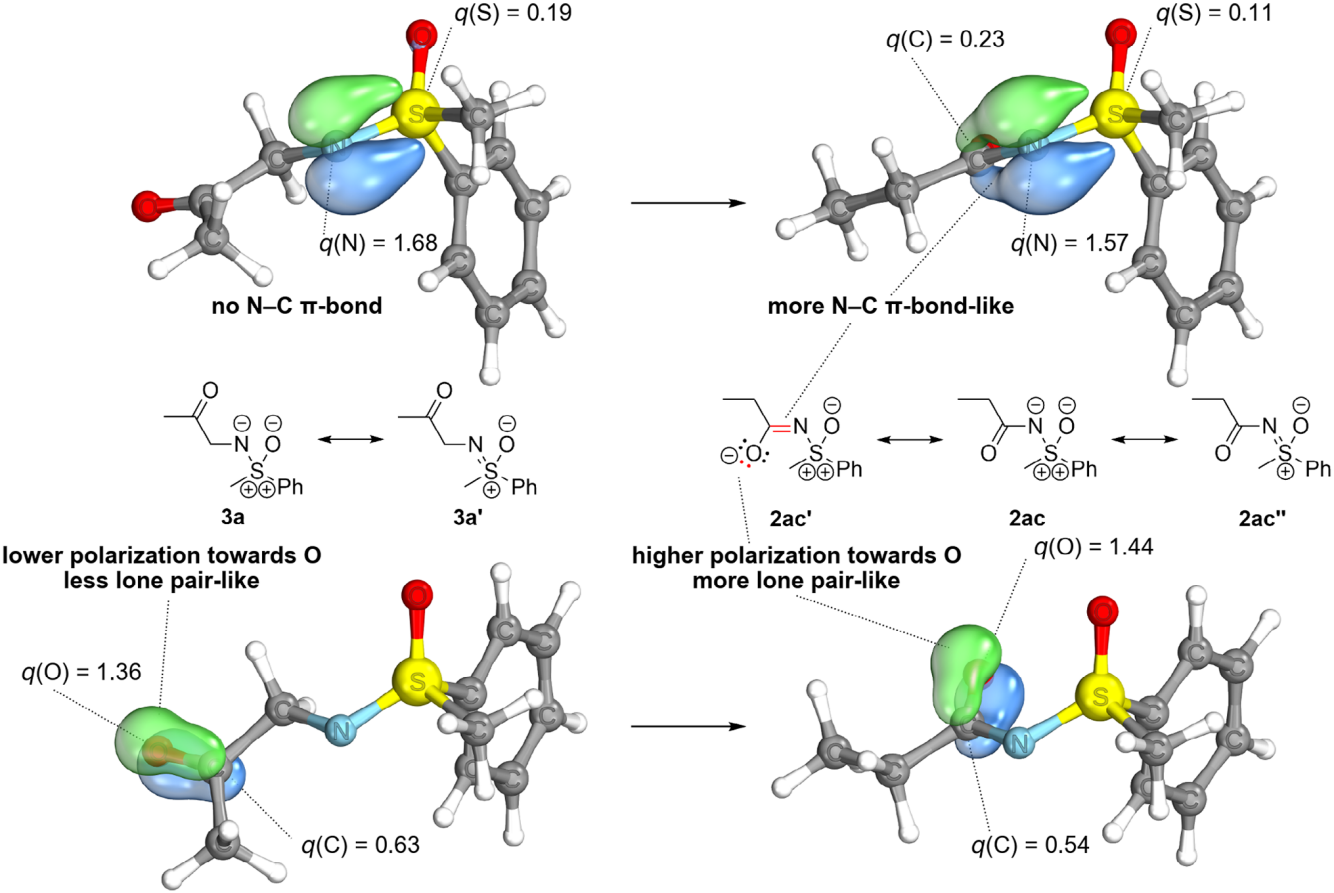
Two most relevant orbital changes in the isomerization of *N*‐(2‐oxopropyl)‐*S*‐methyl‐*S*‐phenyl sulfoximine (**3a**) to *N*‐propionyl‐*S*‐methyl‐*S*‐phenyl sulfoximine (**2ac**), accounting for 47% of the total density change. The changes in the two orbitals reflect the occurrence of a LP‐π^*^ interaction in the product of isomerization, as opposed to the educt.

Analogously, an IBO analysis offers a comparable perspective of the bonding situation. When first performing an IBO analysis of an *N*H‐sulfoximine such as *S*‐methyl‐*S*‐phenyl sulfoximine (**1**) or a substituted sulfoximine such as the *N*‐2‐oxopropyl congener **3a** bearing no or a very weak π‐acceptor in *α*‐position to the sulfoximidoyl nitrogen, the S─N bond description encompasses a two‐center S─N σ‐bond and a two‐center π‐bond, the latter of which is almost completely polarized toward the sulfoximidoyl nitrogen atom (for the S─N π‐bond, see the left structures in Figure 3 and Figure 4). The observation of a highly polarized S─N π‐bond is similar to an IBO assessment of the π‐bonds of formally hypervalent SO_3_ by Knizia and indicates that the S─N bond is closer to a single than to a double bond.^[^ ^57^ ^]^ Compared to *N*H‐sulfoximine **1** (no π‐acceptor), the contributions of the S─N π‐bonds on sulfur are further reduced for the acceptor substituted sulfoximines **2** and significant delocalization of the polarized S─N π‐orbital to the carbon in alpha position to the sulfoximidoyl nitrogen atom can be observed.

**Figure 5:**
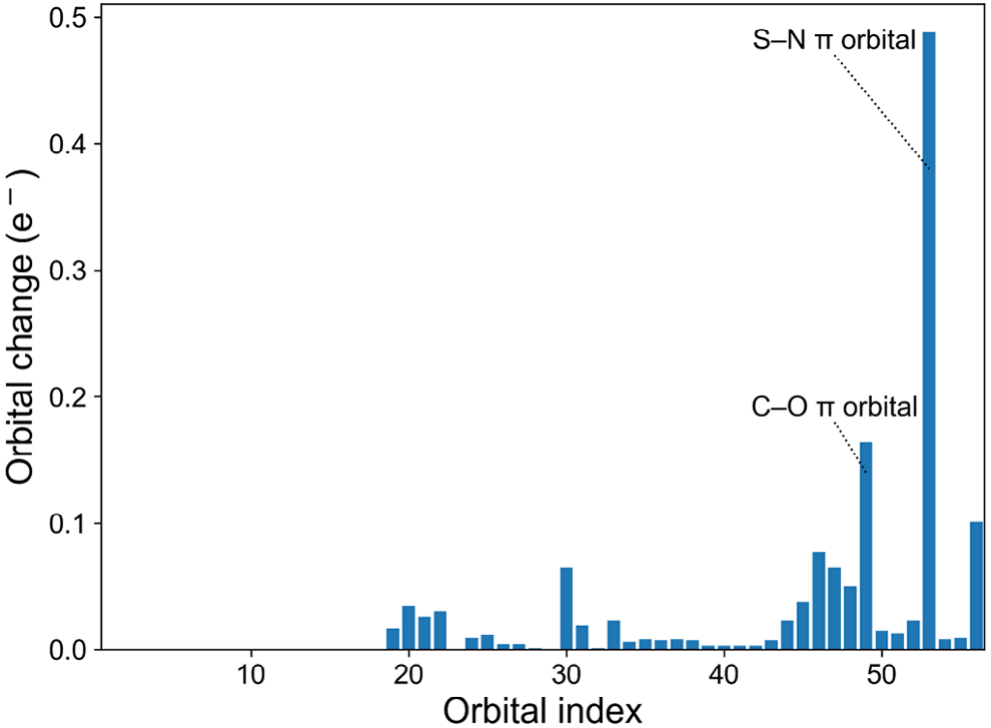
Changes in the intrinsic bond orbitals upon isomerization of *N*‐(2‐oxopropyl)‐*S*‐methyl‐*S*‐phenyl sulfoximine (**3a**) to *N*‐propionyl‐*S*‐methyl‐*S*‐phenyl sulfoximine (**2ac**) (see Scheme 1).

The delocalization described above is reflected in the intrinsic atomic orbital (IAO) partial charges of the S─N π‐bonds in the acceptor substituted sulfoximines, which are nonzero at the carbon atom in *α*‐position to the sulfoximidoyl nitrogen (see Table 1). Additionally, the delocalization of the S─N π‐bond is illustrated in Figure 3 by comparing the S─N π‐bond in *S*‐methyl‐*S*‐phenyl sulfoximine **1** with the corresponding bond in the acceptor substituted *N*‐propionyl congener **2ac** and in Figure 4 by examining the hypothetical isomerization of *N*‐2‐oxopopyl‐*S*‐methyl‐*S*‐phenyl sulfoximine (**3a**) (no LP‐π^*^‐interaction) to the *N*‐propionyl isomer **2ac** (strong LP‐π^*^‐interaction). The significant contribution of the π‐bond on the carbon atom in alpha position to the sulfoximidoyl nitrogen agrees with the resonance picture associated with a significant LP‐π^*^‐interaction (see Figure 3 and Figure 4) and agrees qualitatively with the IBO delocalization of the nitrogen lone pair observed for the reference systems encompassing the dimethylamino donor. The IAO partial charges of the lone pairs in the dimethylamino donor systems are also listed in Table 1 for means of comparison. Besides the S─N π‐bond, the perturbation of the C─O π‐IBO upon isomerization is shown for one example in Figure 4. In analogy to the S─N π‐bond, the changes in the C─O π‐IBO also reflect the expected changes in the composition of the resonance hybrid upon isomerization, i.e., a contribution of resonance form **2ac’** to the structure of the product (see Figure 4). In total, the changes in the S─N π‐orbital and the C─O π‐orbital account for 47% of the total orbital change in the transformation of *N*‐2‐oxopropyl‐*S*‐methyl‐*S*‐phenyl sulfoximine (**3a**) to the *N*‐propionyl congener **2ac** (see Figure 5). In summary, the NBO and IBO analyses consistently provide confirmation for a π‐electron donating effect from the sulfoximidoyl nitrogen atom to the N‐bound substituent.

**Scheme 1:**
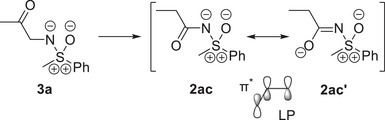
Hypothetical isomerization of *N*‐(2‐oxopropyl)‐*S*‐methyl‐*S*‐phenyl sulfoximine (**3a**) to the *N*‐propionyl congener **2ac**. A LP‐π^*^ interaction is only expected in the product of isomerization.

### Effects of π‐Donation on Conformation

**Figure 6:**
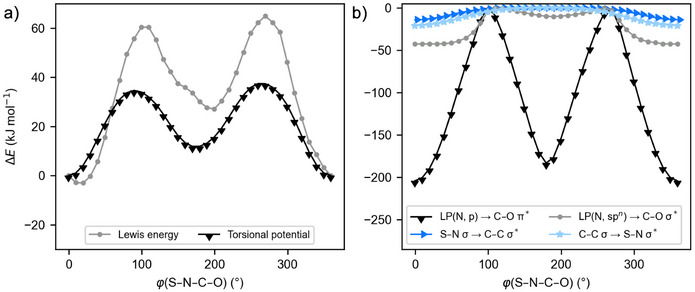
Torsional dependencies of the potential, the Lewis energy and selected resonance stabilization energies in *N*‐propionyl‐*S*‐methyl‐*S*‐phenyl sulfoximine (**2ac**) computed at the B3LYP‐D3/6‐311++G(d,p)//r2SCAN‐3c/def2‐mTZVPP+SMD(DCM) level of theory. a) Torsional potential and its Lewis energy contribution. b) Torsional dependencies of the delocalization energy contributions which exhibit global minima at *ϕ*(S─N─C─O) = 0°.

**Figure 7:**
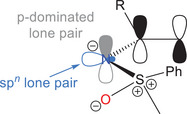
Stabilizing alignment of the p‐dominated lone pair on the sulfoximidoyl nitrogen atom and the π^*^‐orbital of the acceptor, which is observed in the most stable conformers of compounds **2aa**–**2ac**, **2bc**, and **2cc**. In these conformations, the S─N─C─X dihedral angle is close/equal to zero or in one instance 180 degrees.

The quantities from the NBO and IBO analyses in Table 1 were in all cases computed for the lowest energy conformers (details on the conformer search procedure are provided in the Supporting Information, Section 1.2). We became interested in the torsional dependencies of the donor–acceptor interactions involving the two nitrogen lone pairs and how these relate to the geometry of the lowest energy conformation. Focusing on the S─N─C─O dihedral angle *ϕ* in *N*‐propionyl‐*S*‐methyl‐*S*‐phenyl sulfoximine (**2ac**), the torsional potential and its Lewis energy contribution were assessed.^[^ ^61^ ^]^ Additionally, the *ϕ*‐dependencies of the donor–acceptor interactions which delocalize electron density from the sulfoximidoyl fragment to the propionyl group and vice versa were computed. The torsional potential is depicted in Figure 6a and can be described as a double‐well potential with minima at dihedral angles of zero and approximately 180 degrees. The minimum at zero degrees is favored by 12 kJ mol^−1^ over the one at 180 degrees. Approximately, the same double‐well dependence is found for the Lewis energy contribution. Resonance interactions which stabilize the preferred zero‐degree S─N─C─O dihedral angle in the lowest conformer are summarized in Figure 6b. These are primarily π‐donation from the p‐dominated lone pair into the C─O π^*^‐orbital and the interaction of the sp*^n^*‐hybridized nitrogen lone pair with the C─O σ^*^‐orbital. Interactions not included in Figure 6b are either negligible or not stabilizing at *ϕ*(S─N─C─O) = 0°. In conclusion, the geometry of the lowest energy conformer is rationalized, at least to a significant degree, by the stabilizing alignment of the p‐type lone pair with the C─O π^*^‐orbital and, in turn, provides an indication for the +M‐effect at nitrogen (see Figure 7).

### Confirming π‐Donation with Hammett Constants and Hypothetical Isomerization Reactions

Hammett constants are a useful measure for the electron donating properties of a functional group. We computed Hammett constants for three selected sulfoximidoyl donors and, as expected, found these to bear a negative sign. Despite relatively large errors on the computed constants, the comparison of the computed and experimental values in Table 2 suggests that sulfoximidoyl donors are more electron donating than a methoxy group, but less electron donating than a dimethylamine moiety on the Hammett scale.

**Table 2:** Computed Hammett constants for three sulfoximidoyl donors and computed and experimental Hammett constants for other, selected functional groups.

Donor D	Computed Hammet constant^a)^, ^b)^	Experimental Hammet constant^c)^
Me	−0.02 ± 0.21	−0.17
OMe	−0.22 ± 0.21	−0.27
OH	−0.25 ± 0.21	−0.37
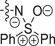	−0.33 ± 0.21	
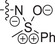	−0.43 ± 0.21	
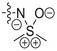	−0.50 ± 0.21	
NMe_2_	−0.81 ± 0.22	−0.83

^a)^ Obtained at the r2SCAN‐3c/def2‐mTZVPP+SMD(water) level of theory by an empirical scaling approach (for details see Supporting Information, Section 1.5).

^b)^ Errors correspond to 95%‐prediction intervals.

^c)^ Obtained from Ref. [62].

**Scheme 2:**
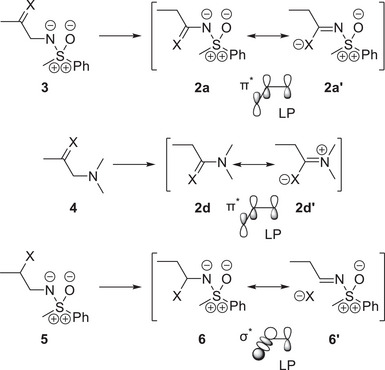
Hypothetical isomerization reactions involving a representative sulfoximidoyl donor group and the dimethylamino donor group. The corresponding energies of isomerization are shown in Figure 8. The donor–acceptor interactions in the products of isomerization can be classified as LP‐π^*^ or LP‐σ^*^ interactions.

Besides the Hammett constants, thermodynamic quantities can be employed to provide additional proof for the π‐electron donating effect outlined above. In this regard, isomerization energies are helpful. The selected reactions in Scheme 2 are homodesmotic; they are comparable as they have the same number and types of bonds and merely differ by the position of the substituent X. Therefore, the most significant difference between structures **3**, **4**, and **5** and their respective isomers **2a**, **2d**, and **6** is the absence or presence of a π‐donor–acceptor interaction. Correspondingly, it can be expected that thermodynamic differences are governed by this interaction.

**Figure 8:**
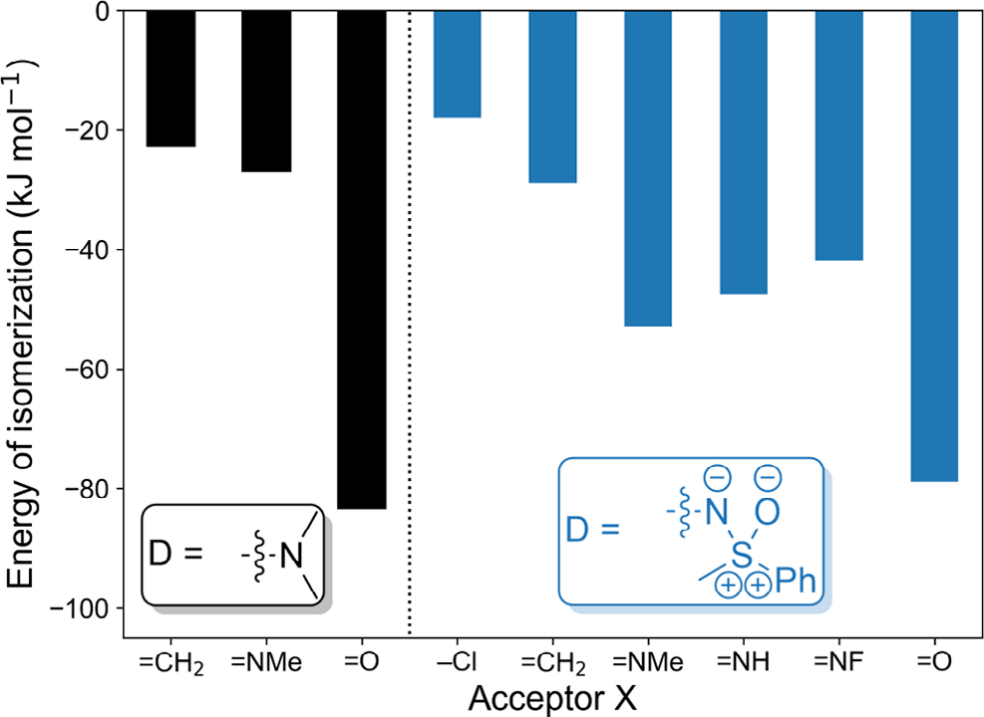
Energies of isomerization computed for the hypothetical isomerization reactions in Scheme 2 at the r2SCAN‐3c/def2‐mTZVPP level of theory in an SMD DCM solvent environment.

Isomerization energies were computed for six isomerization reactions with a sulfoximidoyl donor group and three transformations with a dimethylamino donor functionality, serving as a reference. The computed, negative isomerization energies support a stabilizing impact of the π‐interaction in the conjugated isomers **2a**, **2d**, and **6**, respectively (see Figure 8).

The deprotonation energy (DPE, proton affinity of the conjugated base) of the sulfur‐bound methyl group is another useful probe for the electron richness of the sulfur. We expect a decline upon isomerization due to the accompanying withdrawal of electron density from the sulfoximidoyl sulfur atom. The proton affinity (PA) at the substituent X helps judge its electron‐richness. As expected, decreases are observed for the CH_3_ deprotonation energy, whereas increases are observed for the latter quantity (see Table 3). The significant changes in the deprotonation energies upon isomerization shown in Table 3 align with the strong dependencies of the p*K*_a_ values of aliphatic units in *α*‐position to the sulfoximidoyl sulfur on the substituents at N, which are experimentally well known.^[^ ^5^, ^13^, ^14^, ^15^, ^16^ ^]^ For example, a p*K*_a_ decreases from 33 to 24.5 is observed in DMSO for the sulfur‐bound methyl group when an inductively electron‐donating methyl substituent at N is replaced by an electron‐withdrawing *p*‐toluene sulfonyl group.^[^ ^13^ ^]^ Such p*K*_a_ decrease can largely be attributed to the demonstrated electron‐withdrawing effect of π‐acceptors on the sulfoximidoyl moiety. The changes in the acidity and basicity outlined in Table 3 indicate that the LP‐ π^*^‐interaction strongly affects the chemical behavior of acceptor substituted sulfoximines.

**Table 3:** Changes in the proton affinity (PA) at X of structures **2a** and **3** and the deprotonation energy DPE at the sulfoximidoyl sulfur bound methyl group upon isomerization according to Scheme 2.

(1) $$\begin{array}{c} \Delta \mathrm{PA}=\mathrm{PA}(2)-\mathrm{PA}(3) \end{array}$$ (2) $$\begin{array}{c} \Delta \mathrm{DPE}=\mathrm{DPE}(2)-\mathrm{DPE}(3) \end{array}$$			
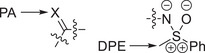			
Acceptor X	Donor D	ΔPA^a)^ (kJ mol^−1^)	ΔDPE^a)^ (kJ mol^−1^)
=CH_2_	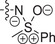	110	−13
=NCH_3_		26	−14
=NH		15	−7
=NF		31	−19
=O		42	−15

^a)^ Computed at the r2SCAN‐3c/def2‐mTZVPP level of theory in an SMD DCM solvent environment.

### Reactions of *N*‐Alkynylated Sulfoximines with Acids

To understand how the +M‐effect of the sulfoximidoyl group affects the reactivity of the nitrogen‐bound substituent, known chemical transformations were examined. We focused on the basicity and nucleophilicity of the substituent. In this regard, *N*‐alkynylated sulfoximines caught our attention since the alkynic moieties in these compounds exhibit well‐documented basic and nucleophilic properties.^[^ ^36^, ^63^, ^64^, ^65^ ^]^

**Scheme 3:**
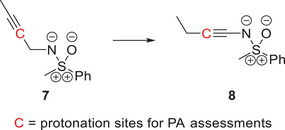
Hypothetical isomerization reaction which was assessed at the r2SCAN‐3c/def2‐mTZVPP level of theory in a THF SMD solvent environment to demonstrate the heightened basicity of the alkynic carbon in *β*‐position to the sulfoximidoyl nitrogen atom in *N*‐alkynylated sulfoximine **8**.

**Figure 9:**
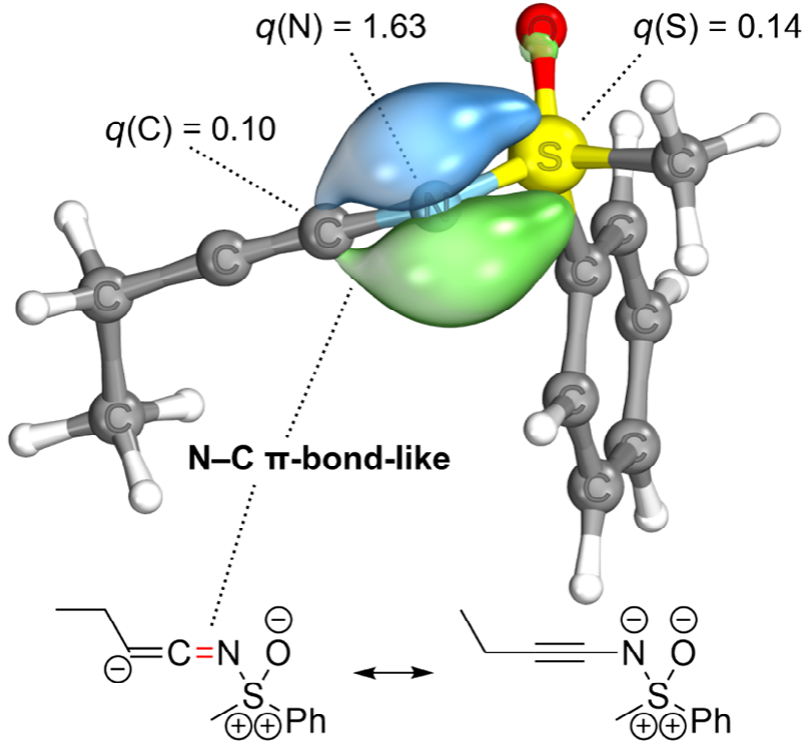
Geometry and S∼‐N πIBO of N‐alkylated sulfoximine **8**, both obtained at the r2SCAN‐3c/def2‐mTZVPP+SMD(THF) level of theory. The depicted orbital indicates that an electron flow to the substituent occurs.

In analogy to the previous assessments, the elevated proton affinity at the substituent of these compounds can be revealed by considering a hypothetical isomerization, e.g., the reaction in Scheme 3. For the depicted example, the proton affinity of the alkynic carbon in *β*‐position to the sulfoximidoyl nitrogen increases by 149 kJ mol^−1^ upon isomerization from structure **7** to *N*‐alkynylated sulfoximine **8**, which agrees with a positive mesomeric effect of the sulfoximidoyl group. In line with previous results, the LP‐π^*^‐interaction results in a negative isomerization energy of −19 kJ mol^−1^ and an electron flow to the substituent is indicated by the delocalization of the S─N π‐IBO to the nitrogen‐bound alkynic carbon atom in compound **8** (see Figure 9).

**Scheme 4:**
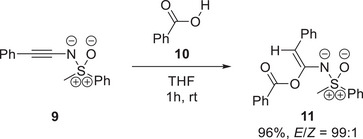
Addition of benzoic acid **10** to *N*‐alkynylated sulfoximine **9** in THF at room temperature.^[^ ^64^ ^]^

The elevated basicity of sulfoximidoyl‐substituted alkynes enabled our group to develop addition reactions of acids to *N*‐alkynylated sulfoximines, of which one representative example is shown in Scheme 4.^[^ ^64^ ^]^ We had limited understanding of the reaction at the time and aim to provide deeper insights into this transformation with the new perspective presented herein.

**Figure 10:**
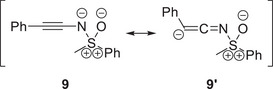
Two selected resonance structures of *N*‐alkynylated sulfoximine **9**.

In Scheme 4, benzoic acid **10** is added to *N*‐alkynylated sulfoximine **9** to provide adduct **11** predominantly in an (*E*)‐configuration. Although the reaction is shown for the specific substrates **9** and **10**, the transformation can also be performed with derivatives thereof, resulting in yields ranging from 60% to 99% and *E*/*Z* ratios exceeding 95:5 in all cases.^[^ ^64^ ^]^ As can be observed from Scheme 4, the proton is thereby introduced at the alkynic carbon atom in *β*‐position to the sulfoximidoyl nitrogen. The observed regioselectivity agrees with a 112 kJ mol^−1^ higher proton affinity which was computed for the *β*‐position as opposed to the alkynic *α*‐carbon (compare with resonance structure **9′** in Figure 10).

**Figure 11:**
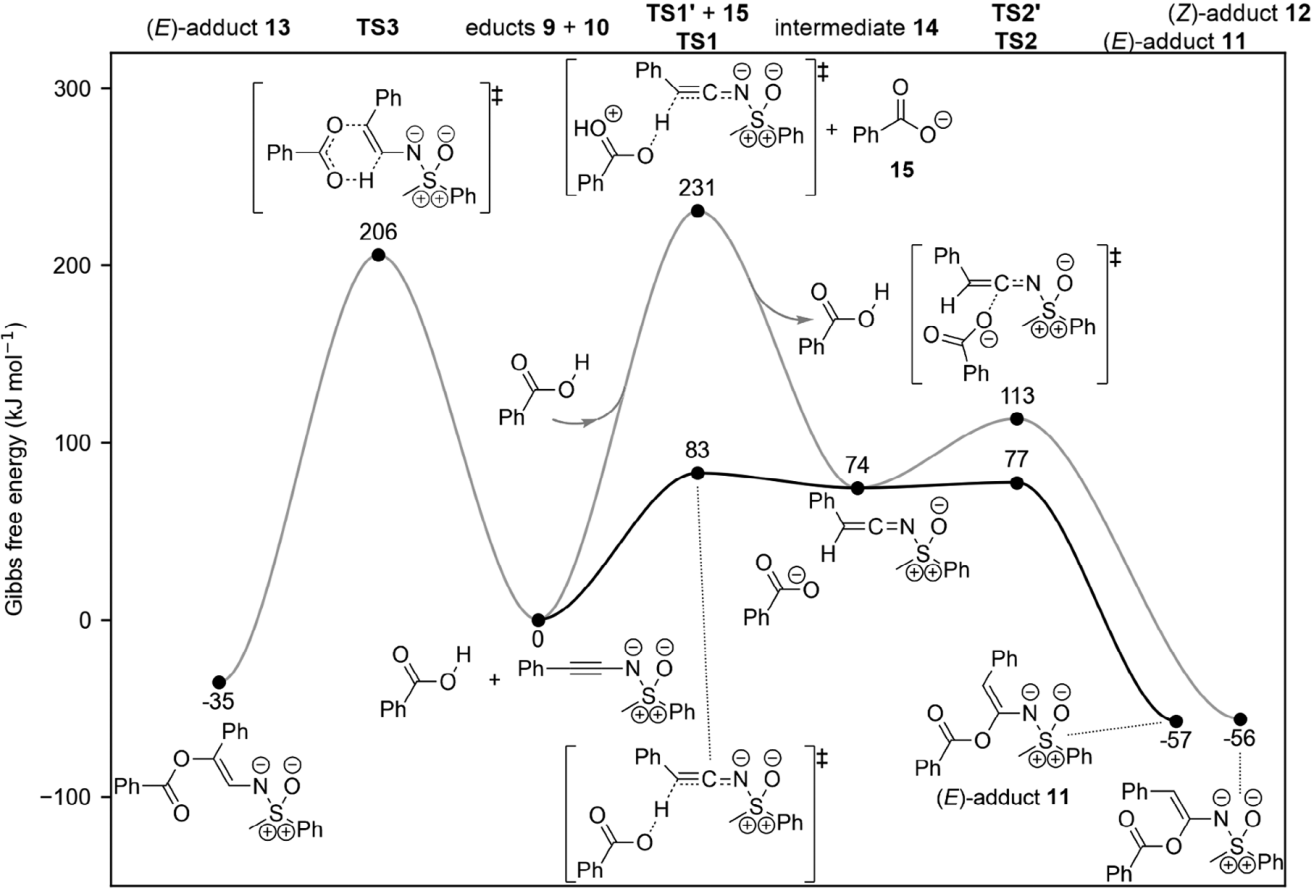
Gibbs energy profiles computed at the ωB97M‐V/def2‐QZVP//PBE0‐D3/def2‐SVP+SMD(THF) level of theory for the reactions of *N*‐alkynylated sulfoximine **9** with benzoic acid **10**, leading to the adducts **11** (experimentally observed), and **12** and **13** (not observed experimentally). Gibbs energies were computed at a temperature of 298.15 K and for a concentration of 1 mol L^−1^ in solution.

To elucidate whether sequential or concerted processes occur in the reactions leading to structure **11**, the alternative (*Z*)‐adduct **12** and the *α*‐protonated product **13**, a DFT analysis of the mechanism was conducted. First, the barriers of the sequential mechanism leading to (*E*)‐adduct **11** were assessed and were indeed found to be appropriate for a transformation conducted at room temperature (Figure 11, Sections 5.1.1 and 5.1.2 in the Supporting Information). The proton transfer was identified as the rate‐limiting step in this mechanism. Since the cation and the benzoate anion resulting from the rate‐limiting and charge separating *β*‐protonation exhibit high stabilities, the process is overall feasible at room temperature, even when a relatively apolar solvent such as THF is employed. The high ion stabilities imply a relatively stable transition state of the rate‐limiting step, which in accordance with the Hammond postulate resembles structurally and energetically the ion pair intermediate **14**.^[^ ^66^ ^]^ The preferential formation of the (*E*)‐configured adduct **11** over the (*Z*)‐adduct **12** from the common intermediate **14** is explained by a lower Gibbs energy barrier in the second reaction step (77 kJ mol^−1^ vs. 113 kJ mol^−1^; Figure 11). The involvement of protonated benzoic acid in the rate‐limiting protonation of the alkyne was ruled out based on a significantly higher Gibbs energy barrier of 231 kJ mol^−1^ compared to the 83 kJ mol^−1^ found for the reaction with neutral benzoic acid. All attempts to locate a transition state for a concerted mechanism leading directly from substrates **9** and **10** to product **11** were unsuccessful. Additionally, a relaxed surface scan indicated that a concerted process involves a significantly higher energy barrier (see Supporting Information, Section 5.1.3).

Unlike the sequential process leading to adduct **11**, formation of the *α*‐protonated structure **13** proceeds via a concerted mechanism (Figure 11). The concerted nature of the mechanism leading to alkene **13** is not unexpected and aligns with the 112 kJ mol^−1^ lower stability of the cation which is generated by protonation of the alkynic carbon atom in *α*‐position to the sulfoximidoyl nitrogen. Thereby, the partial proton transfer in the transition state and the resulting formation of a hole force the carbonyl oxygen atom in benzoic acid to immediately stabilize the, otherwise highly unstable, cation, resulting in a concerted bond breaking and bond forming process (energetically coupled processes, see Section 5.1.4 in the Supporting Information). Evidently, the aforementioned stabilization is not sufficient to lower the Gibbs energy barrier below the barrier of 83 kJ mol^−1^ of the mechanism leading to the experimentally observed adduct **11** or is offset by other factors, e.g. steric repulsion, and hence a significantly higher barrier of 206 kJ mol^−1^ is observed for the reaction leading to alternative structure **13**, which is in line with experimental observation.

Thus, the regioselectivity observed in the addition of benzoic acid **10** to *N*‐alkynylated sulfoximine **9** can be understood from the significantly higher stability of the cation formed by protonation of the alkynic carbon atom in *β*‐position to the sulfoximidoyl nitrogen, which is a direct consequence of the favorable LP‐π^*^ interaction delineated herein. The stability of the *β*‐protonated cation not only renders the addition of benzoic acid feasible at room temperature but also results in a sequential process instead of a concerted reaction pathway.

Finally, the effect of different donating groups on the Gibbs energy barrier of the considered reaction is demonstrated in Table 4. Therein, it is shown that additions of benzoic acid to methoxy, sulfoximidoyl, and dimethyl amino substituted alkynes (i.e., alkynes bearing mesomeric donors) proceed with significantly reduced Gibbs energy barriers compared to the unsubstituted congener. The order of the barriers coincides with the order of the Hammett constants reported in Table 2.

**Table 4:** Gibbs energy barriers for the rate‐limiting step in the additions of benzoic acid to phenylacetylenes with alkyne‐bound donors. In the considered reactions, the proton is introduced in *β*‐position to the donor D.

Donor D	Δ*G* ^‡^ (kJ mol^−1^)^a)^
H	249
OMe	122
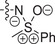	83
NMe_2_	56

^a)^ Computed at the ωB97M‐V/def2‐QZVP//PBE0‐D3/def2‐SVP+SMD(THF) level of theory.

### Reactions of Acceptor‐Substituted Sulfoximines with More Complex Electrophiles

**Scheme 5:**
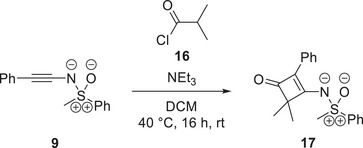
Cyclization of *N*‐alkynylated sulfoximine **9** with dimethyl ketene, which is formed in‐situ from isobutyryl chloride (**16**) in the presence of base.^[^ ^63^ ^]^

Additionally, the reaction shown in Scheme 5 caught our attention. The depicted transformation was developed by our group and was previously interpreted as a [2+2]‐cycloaddition reaction of in‐situ formed ketenes with *N*‐alkynylated sulfoximines.^[^ ^63^ ^]^ The observed regioselectivity reignited our interest since it aligns with the +M‐effect of the sulfoximidoyl group.

**Figure 12:**
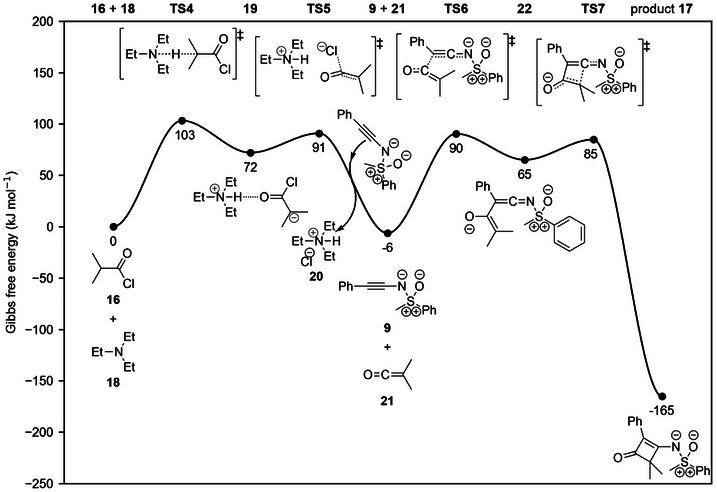
Gibbs energy profile for the in‐situ formation of dimethyl ketene (**21**) from isobutyryl chloride (**16**) and the subsequent cyclization with *N*‐alkynylated sulfoximine **9** to product **17** assessed at the ωB97M‐V/def2‐QZVP//PBE0‐D3/def2‐SVP+SMD(DCM) level of theory. Gibbs energies were computed at a temperature of 313.15 K and for a concentration of 1 mol L^−1^ in solution.

The mechanism of the reaction depicted in Scheme 5 was evaluated at the DFT level and, instead of the concerted [2+2]‐cycloaddition that was previously proposed, a sequential process was found for the cyclization of dimethyl ketene with sulfoximine **9** (Figure 12, Section 5.2.1 in the Supporting Information). The DFT determined Gibbs energy barriers are of the correct magnitude for a reaction at 40 °C (Section 5.2.2 in the Supporting Information). In the first step of the process, a zwitterionic intermediate is formed by electrophilic attack of the carbonyl carbon of dimethyl ketene at the alkynic carbon atom in *β*‐position to sulfoximidoyl nitrogen. In the second step, the zwitterionic intermediate then cyclizes to the final product. The electrophilic attack occurs at the alkynic carbon atom in *β*‐position to the sulfoximidoyl nitrogen, which is rendered basic and nucleophilic by the π‐donating effect discussed herein. Hence, similar to the addition of benzoic acid, the regioselectivity of the cyclization can be understood solely on the basis of the LP‐π^*^‐interaction. Potential alternative mechanisms leading to cyclization product **17** were found to be less likely (Sections 5.2.3 to 5.2.5 in the Supporting Information).

## Conclusion

In the current article, evidence for a significant donation of the p‐dominated lone pair of the sulfoximidoyl nitrogen atom to π‐acceptor orbitals was presented. Our analysis suggests that this ubiquitous functional group, previously assumed to be electron‐withdrawing, is actually a mesomeric donor. This knowledge can have a significant impact on the planning of syntheses and in rationalizing reaction outcomes in the field of sulfoximine chemistry. The LP‐π^*^‐interaction was demonstrated with second order perturbation energies and occupation numbers of NBO analyses of acceptor substituted sulfoximines. This was further supported by IBO analyses, revealing electron flow from the S─N π‐orbital to the nitrogen‐bound substituent. This induced a higher computed basicity of the substituent and higher acidity at the sulfoximidoyl sulfur‐bound methyl group. Ultimately, it was shown that the LP‐π^*^‐interaction described herein provides a rationale for known reaction patterns of π‐acceptor‐substituted sulfoximines. The key takeaway is that sulfoximidoyl groups are electron‐donating groups, primarily introducing basicity and nucleophilicity to conjugated substituents.

## Supporting Information

The authors have cited additional references within the Supporting Information.^[^ ^67^, ^68^, ^69^, ^70^, ^71^, ^72^, ^73^, ^74^, ^75^, ^76^, ^77^, ^78^, ^79^, ^80^, ^81^, ^82^, ^83^, ^84^, ^85^, ^86^, ^87^, ^88^, ^89^, ^90^, ^91^, ^92^, ^93^, ^94^, ^95^, ^96^, ^97^, ^98^, ^99^, ^100^, ^101^, ^102^, ^103^, ^104^, ^105^, ^106^, ^107^, ^108^, ^109^, ^110^, ^111^, ^112^, ^113^, ^114^, ^115^, ^116^, ^117^, ^118^, ^119^, ^120^, ^121^, ^122^, ^123^, ^124^, ^125^, ^126^ ^]^

## Conflict of Interests

The authors declare no conflict of interest.

## Supporting information



Supporting Information

## Data Availability

The data that support the findings of this study are available in the Supporting Information of this article.
