# Supplementary material for: π‐Electron Donation at the Sulfoximidoyl Nitrogen Atom
Source: Angew Chem Int Ed Engl. 2025 Jun 23;64(33):e202510744. doi: 10.1002/anie.202510744 (PMC12338397; doi:10.1002/anie.202510744)
Supplement: Supplementary file 1 — Supporting Information [file ANIE-64-e202510744-s001.pdf]

Supporting Information
©Wiley-VCH 2021
69451 Weinheim, Germany

Table of Contents

1. Computational details S3

1.1 General information S3

1.2 Conformational analyses S3

1.3 NBO and IBO analyses (Table 1, Figure 3, Figure 4, Figure 9, Figure S2, Figure S3 and Tables S1 to S4) S3

1.4 Torsional potential and Lewis and delocalization energy contributions to the torsional potential (Figure 6 and Figure S6) S4

1.5 Hammett constants (Table 2) S4

1.6 Isomerization energies, proton affinities and deprotonation energies (Figure 8, Table 3 and text) S5

1.7 Gibbs energy profiles of reaction mechanisms (Figure 11, Figure 12, Figure S7, Figure S15, Figure S16 and Figure S21) S5

2 NBO analysis S6

2.1 Lone pair NBOs on nitrogen S6

2.2 $E^{(2)}$-Energies and lowest energy conformers (compounds 2aa–2ac, 2bc, 2cc and 2da–2dc) S7

3 IBO analysis—Calculation of orbital change in a hypothetical isomerization reaction S11

4 Torsional potential and Lewis and delocalization energy contributions to the potential (Figure S6)—Supplemental results S13

5 Mechanistic studies—Results S14

5.1 Addition of benzoic acid (**10**) to *N*-alkynylated sulfoximine **9** S14

5.1.1 Energies, thermostatistical corrections and Gibbs energies S14

5.1.2 Kinetic analysis (Figure 11, Figure S7) S16

5.1.3 Relaxed surface scan—Concerted formation of (*E*)-adduct **11** S17

5.1.4 Relaxed surface scan—Formation of alternative (*E*)-adduct **13** by consecutive proton transfer and C–O bond formation S18

5.1.5 Effect of different donors on the Gibbs energy barrier of the benzoic acid addition S21

5.2 Cyclization of *N*-alkynylated sulfoximine **9** with isobutyryl chloride (**16**) S23

5.2.1 Energies, thermostatistical corrections and Gibbs energies for the lowest Gibbs energy pathway S23

5.2.2 Kinetic analysis (Figure 12, Figure S16) S25

5.2.3 Relaxed surface scan—Concerted formation of cyclic product **17** (alternative mechanism 1) S28

5.2.4 Cyclization of *N*-alkynylated sulfoximine **9** with isobutyryl chloride (**16**)—Results for alternative mechanism 2 S29

5.2.5 Cyclization of *N*-alkynylated sulfoximine **9** with isobutyryl chloride (**16**)—Results for alternative mechanism 3 S31

6 References S34

7 Cartesian coordinates S35

7.1 Addition of benzoic acid (**10**) to *N*-alkynylated sulfoximine **9**—Cartesian coordinates S35

7.2 Cyclization of *N*-alkynylated sulfoximine **9** with isobutyryl chloride (**16**)—Cartesian coordinates S50

7.3 Cyclization of *N*-alkynylated sulfoximine **9** with isobutyryl chloride (**16**) (alternative mechanism 2)—Cartesian coordinates S58

1 Computational details

1.1 General information

Semi-empirical calculations were performed with the xTB 6.4.0 program.[S1–S4] The CREST 2.11.2 program was utilized in conjunction with xTB for the generation of conformers.[S5] If not noted otherwise, the default settings were utilized within the two programs. Unless otherwise indicated, DFT (density functional theory) calculations were performed within ORCA 5.0.3 employing the default settings within the program.[S6] The resolution of identity approximation was consistently employed for the Coulomb integrals throughout this work.[S7] Furthermore, the COSX approximation was employed for the Hartree Fock exchange in hybrid DFT calculations.[S8]

1.2 Conformational analyses

If not noted otherwise conformational analyses were conducted for potential energy surface (PES) minima. Conformer generation was performed with the CREST 2.11.2 program in combination with the xTB 6.4.0 program.[S1–S5] Within the programs, the GFN2-xTB semi-empirical method was employed to generate the conformer ensembles.[S1–S4] The solvent environment was taken into account with the ALPB implicit solvent model.[S3] Input structures for the CREST conformer search runs were pre-optimized at the same level of theory as utilized in the actual run. For the conformer generation of non-covalent complexes, the NCI mode within CREST was additionally employed.[S5] All conformers within 6.0 kcal/mol of the lowest conformer were retained after the CREST runs and were subsequently sorted by energy at higher DFT levels of theory.[S9,S10] In these higher level DFT sorting procedures, the solvent environment was taken into account with the solvation model based on density (SMD) from the Truhlar group (referred to as DFT+SMD level hereafter).[S11] Thermostatistical corrections were additionally computed during these sorting procedures at the GFN2-xTB level of theory in an ALPB continuum solvent environment (referred to as GFN2-xTB+ALPB level hereafter) within xTB 6.4.0.[S1–S4,S12]

A three-step procedure was utilized to sort the CREST-generated conformers of PES minima according to their energy and Gibbs energy. In the first step, single point energies were computed at the r2SCAN-3c/def2-mTZVPP+SMD level of theory for the CREST generated conformations.[S2a,S2b,S11,S13,S14] All conformers within 3.5 kcal/mol of the lowest structure were retained for evaluation in step two. In the second step, thermostatistical contributions were additionally computed at the GFN2-xTB+ALPB level and were added to the single point energies obtained in step one.[S1–S4,S12] The resulting Gibbs energies were utilized to sort the conformers and all conformations within a Gibbs energy of 3.5 kcal/mol of the lowest structure were subsequently evaluated in step three. In the third step, all remaining conformers were optimized at the r2SCAN-3c/def2-mTZVPP+SMD or the PBE0-D3/def2-SVP+SMD level of theory.[S2a,S2b,S11,S13–S18] The latter method was utilized for conformers subsequently employed in the evaluation of Gibbs energy profiles. After the optimization, thermostatistical corrections were calculated for the optimized geometries at the GFN2-xTB+ALPB level of theory by employing the single-point hessian approach from the Grimme group.[S1–S4,S12] The thermostatistical corrections were added to the DFT energies of the optimized structures and the resulting Gibbs energies were employed to identify the lowest conformers.

DFT optimized geometries of lowest conformers obtained after the conformational analyses were always confirmed as true minima on the potential energy surface (PES) by numerical frequency calculations.[S19] Structures evaluated during the conformational analyses were not subjected to this scrutiny.

1.3 NBO and IBO analyses (Table 1, Figure 3, Figure 4, Figure 9, Figure S2, Figure S3 and Tables S1 to S4)

The IBO (intrinsic bond orbital) analyses were conducted within the IboView program from Gerald Knizia, utilizing the orbital information from the r2SCAN-3c/def2-mTZVPP+SMD-optimized, lowest conformers.[S20] A DCM (IBO analyses in Table 1, Figure 3 and Figure 4) or a THF (IBO analysis in Figure 9) solvent environment was taken into account with the SMD model in the optimizations. An exponent of 2 was utilized in the localization constraint. Isosurfaces of IBOs in the manuscript encompass 67% (Figure 3 and Figure 4) or 73% (Figure 9) of the respective orbital's density. For the NBO (natural bond orbital) analyses, the lowest conformers from the conformer search procedure were re-optimized in DCM at the B3LYP-D3/6-311++G(d,p)+SMD level of theory within Gaussian 16.[S11,S16,S21–S24] After the optimization, the obtainment of minima on the PES was corroborated with frequency calculations. Subsequently, the NBO analyses were conducted for the optimized structures in DCM with the NBO 3.1 program within Gaussian 16 at the B3LYP-D3/6-311++G(d,p)+SMD level of theory.[S25,S26]

1.4 Torsional potential and Lewis and delocalization energy contributions to the torsional potential (Figure 6 and Figure S6)

The torsional potential in Figure 6 in the manuscript was obtained in the following way. Firstly, a relaxed surface scan was performed at the r2SCAN-3c/def2-mTZVPP+SMD(DCM) level within ORCA 5.0.3. Secondly, single point energy evaluations at the B3LYP-D3/6-311++G(d,p)+SMD(DCM) level of theory were performed within Gaussian 16 by utilizing the relaxed structures from the scan. The Lewis energy and delocalization energy contributions to the potential were assessed with the NBO 3.1 program within Gaussian 16 via the NBO deletion method at the B3LYP-D3/6-311++G(d,p)+SMD(DCM) level of theory.[S25f] The use of symmetry was actively disallowed during these assessments via the nosymm keyword.

1.5 Hammett constants (Table 2)

Hammett constants were computed at the r2SCAN-3c/def2-mTZVPP+SMD(water) level of theory from the Gibbs energies of the optimized geometries of *para*-substituted benzoic acids and their deprotonated congeners according to Equation S1. Hammett constants obtained in this way were subsequently corrected for systematic error with the help of Equation S2. Linear regression parameters in Equation S2 were determined by plotting 14 computed Hammett constants versus their experimentally determined counterparts (experimental Hammett constants were taken from reference S27) and performing a least-squares fit to the data. Prediction intervals for the computed Hammett constants were calculated according to Equation S3.

$$\sigma_{\mathrm{p},i} = \frac{\mathrm{DPG_{ref}} - \mathrm{DPG}_i}{\ln(10)\,RT}$$ **Equation S1**

$$\sigma_{\mathrm{p},i}^{\mathrm{scaled}} = 0.3769 \cdot \sigma_{\mathrm{p},i} - 0.0323$$ **Equation S2**

$$\sigma_{\mathrm{p},h}^{\mathrm{scaled}} \pm t_{\alpha/2,n-2}\sqrt{\frac{\mathrm{SSE}}{n-2}\cdot\left(1+\frac{1}{n}+\frac{\left(\sigma_{\mathrm{p},h}-\overline{\sigma_{\mathrm{p}}}\right)^2}{\sum_i\left(\sigma_{\mathrm{p},i}-\overline{\sigma_{\mathrm{p}}}\right)^2}\right)}$$ **Equation S3**

$\sigma_{\mathrm{p},i}$ = Computed Hammett constant for the *para*-position ().

$\mathrm{DPG_{ref}}$ = Gibbs energy of deprotonation of benzoic acid in water (J/mol).

DPG_i = Gibbs energy of deprotonation of a *para*-substituted benzoic acid derivative *i* (J/mol).

R = Molar gas constant ($\mathrm{J{\cdot}K^{-1}{\cdot}mol^{-1}}$)

T = Temperature (K)

$\sigma_{\mathrm{p},i}^{\mathrm{scaled}}$ = Scaled Hammett constant for the *para*-position ().

$t_{\alpha/2,n-2}$ = Critical value of Student's *t*-statistic ().

SSE = Error sum of squares ().

n = Degrees of freedom ().

$\overline{\sigma_{\mathrm{p}}}$ = Mean value of the computed Hammet constants in the data set ().

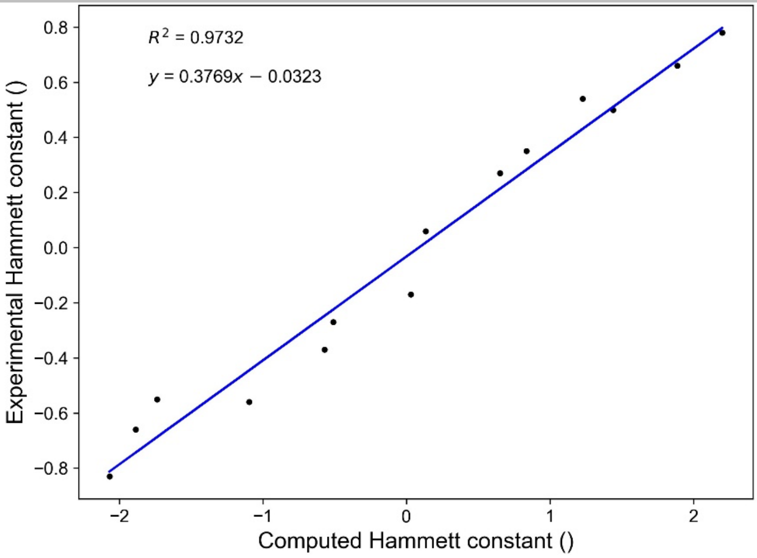

Figure S1. Plot of computed versus experimental Hammett constants for 14 *para*-substituted benzoic acids (black) and linear least-squares fit (blue). The computed Hammett constants were obtained through Equation S1 at the r2SCAN-3c/def2-mTZVPP+SMD(water) level of theory.

1.6 Isomerization energies, proton affinities and deprotonation energies (Figure 8, Table 3 and text)

If not noted otherwise, isomerization energies, proton affinities and deprotonation energies were computed at the r2SCAN-3c/def2-mTZVPP+SMD level of theory, either in a DCM or a THF continuum solvent environment.[S2a,S2b,S11,S13,S14] Proton affinities and deprotonation energies were computed by protonating or deprotonating the educts/products of isomerization and subsequently re-optimizing the resulting structures at the r2SCAN-3c/def2-mTZVPP+SMD level of theory.[S2a,S2b,S11,S13,S14] For the protonated/deprotonated structures no conformational analyses were conducted.

The proton affinity of *N*-alkynylated sulfoximine **9** in THF was evaluated at the ωB97M-V/def2-QZVP//PBE0-D3/def2-SVP+SMD(THF) level of theory and its assessment constitutes an exception to the scheme outlined above.[S11,S15–S18,S28–S30] Here, lowest conformers were identified by the routine outlined in section 1.2 for sulfoximine **9** and its protonated congener with one difference: the PBE0-D3/def2-SVP+SMD level of theory was employed in the DFT optimization step of the energetic sorting procedure. Both lowest free energy conformers were then re-optimized at the PBE0-D3/def2-SVP+SMD level of theory with enhanced numerical grid settings and SCF (self-consistent field) convergence thresholds corresponding to the DEFGRID3 and the VeryTightSCF setting, respectively.[S11,S15–S18] Proton affinities were subsequently computed from the ωB97M-V/def2-QZVP+SMD single point energies of the neutral and the protonated compound which were assessed at the PBE0-D3/def2-SVP+SMD optimized geometries with the same numerical grid settings and SCF convergence thresholds as in the optimization.[S11,S17,S18,S28–S30]

1.7 Gibbs energy profiles of reaction mechanisms (Figure 11, Figure 12, Figure S7, Figure S15, Figure S16 and Figure S21)

Geometry optimizations of PES minima and transition states were performed at the PBE0-D3/def2-SVP+SMD level of theory, with an SCF convergence threshold corresponding to the VeryTightSCF setting and the more accurate DEFGRID3 numerical grid within ORCA 5.0.3.[S11,S15–S18] In all cases, numerical frequency calculations were performed at the same level of theory to corroborate the expected nature of the stationary point (zero imaginary frequencies for minima, one imaginary frequency for transition states).[S19] Guess structures for transition state optimizations were usually generated with the nudged elastic band climbing image (NEB-CI) algorithm within ORCA 5.0.3.[S31] Intrinsic reaction coordinate (IRC) calculations were conducted for transition states at the same level of theory as in the optimization to confirm that the transition states were connected to the expected minima on the PES.[S32] Ultimately, single point energies were calculated at the ωB97M-V/def2-QZVP+SMD level of theory with the same SCF convergence thresholds and numerical grid setting as in the optimization utilizing the PBE0-D3/def2-SVP+SMD optimized geometries.[S11,S17,S18,S28–S30] Thermostatistical corrections obtained at the PBE0-D3/def2-SVP+SMD level of theory were added to the single point energies computed at the ωB97M-V/def2-QZVP+SMD level of theory to obtain Gibbs energies.

2 NBO analysis

2.1 Lone pair NBOs on nitrogen

The lone pair NBOs on the sulfoximidoyl nitrogen are shown for the exemplary acceptor substituted sulfoximine *N*-propionyl-*S*-methyl-*S*-phenyl sulfoximine (**2ac**) in Figure S2 and Figure S3. The orbitals are shown for an isovalue of 0.04. The images were made with the GaussView 6.1.1 program.[S33]

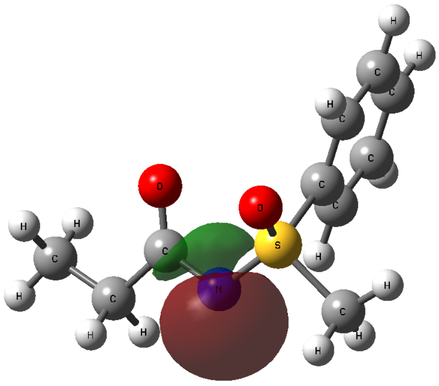

Figure S2. Lone pair NBO on nitrogen with approximately an $sp^{1.4}$-hybridization (41.34% s, 58.60% p, 0.06% d).

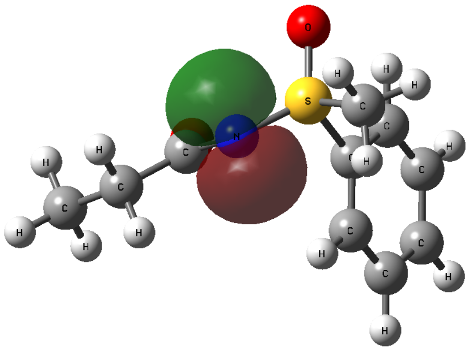

Figure S3. Lone pair NBO on nitrogen corresponding to a p-dominated orbital (0.04% s, 99.86% p, 0.10% d).

2.2 $E^{(2)}$-Energies and lowest energy conformers (compounds 2aa–2ac, 2bc, 2cc and 2da–2dc)

All significant second order energies $E^{(2)}$ of the interactions of the two lone pair NBOs on the sulfoximidoyl nitrogen with adjacent acceptor orbitals and the geometries of the lowest conformers are included in Tables S1–S4 for sulfoximines **2aa**–**2ac**, **2bc** and **2cc**. Additionally, all significant second order energies $E^{(2)}$ of the interactions of the lone pair NBO on the amino nitrogen with adjacent acceptors and the geometries of the lowest conformers are included in Tables S1–S3 for compounds **2da**–**2dc**.

Table S1**:** Second order energies of the interactions of the two nitrogen lone pair NBOs in sulfoximine **2aa** and the nitrogen lone pair NBO in reference compound **2da** with adjacent acceptor orbitals.

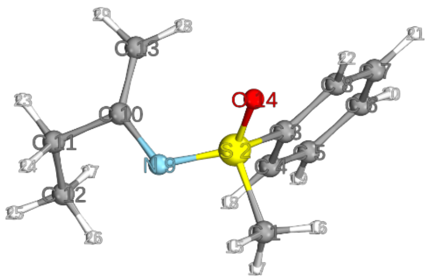			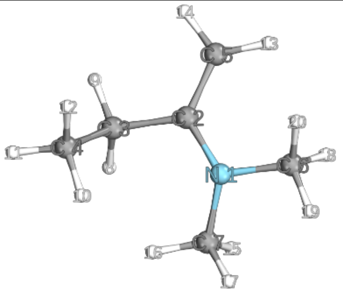		
Donor orbital	Acceptor orbital	$E^{(2)}$ (kJ/mol)	Donor orbital	Acceptor orbital	$E^{(2)}$ (kJ/mol)
LP (N9, sp^n-type)	Ry* (S2)	−23	LP (N1)	Ry* (C2)	−11
LP (N9, sp^n-type)	Ry* (C10)	−10	LP (N1)	Ry* (C6)	−4
LP (N9, sp^n-type)	Ry* (C10)	−8	LP (N1)	Ry* (C6)	−5
LP (N9, sp^n-type)	σ^* (C1–S2)	−16	LP (N1)	Ry* (C7)	−9
LP (N9, sp^n-type)	σ^* (C1–H16)	−3	LP (N1)	σ^* (C2–C5)	−12
LP (N9, sp^n-type)	σ^* (S2–C3)	−18	LP (N1)	π^* (C2–C5)	−118
LP (N9, sp^n-type)	σ^* (S2–O14)	−22	LP (N1)	σ^* (C6–H18)	−34
LP (N9, sp^n-type)	σ^* (C10–C11)	−4	LP (N1)	σ^* (C6–H19)	−5
LP (N9, sp^n-type)	σ^* (C10–C13)	−31	LP (N1)	σ^* (C6–H20)	−8
LP (N9, p-type)	Ry* (S2)	−30	LP (N1)	σ^* (C7–H15)	−33
LP (N9, p-type)	Ry* (S2)	−4	LP (N1)	σ^* (C7–H16)	−5
LP (N9, p-type)	Ry* (S2)	−2	LP (N1)	σ^* (C7–H17)	−7
LP (N9, p-type)	Ry* (S2)	−3			
LP (N9, p-type)	Ry* (C10)	−8			
LP (N9, p-type)	Ry* (C10)	−3			
LP (N9, p-type)	σ^* (S2–C3)	−54			
LP (N9, p-type)	σ^* (S2–O14)	−71			
LP (N9, p-type)	π^* (C10–C13)	−168			

Table S2: Second order energies of the interactions of the two nitrogen lone pair NBOs in sulfoximine **2ab** and the nitrogen lone pair NBO in reference compound **2db** with adjacent acceptor orbitals.

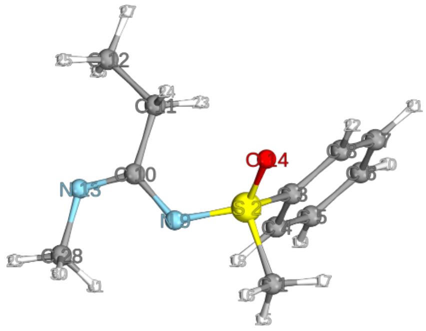

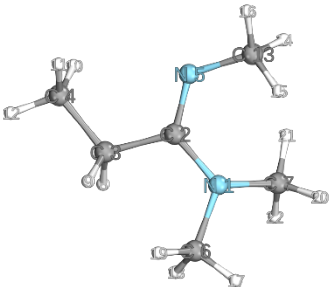

Donor orbital	Acceptor orbital	$E^{(2)}$ (kJ/mol)	Donor orbital	Acceptor orbital	$E^{(2)}$ (kJ/mol)
LP (N9, sp^n-type)	Ry^* (S2)	−23	LP (N1)	Ry^* (C2)	−4
LP (N9, sp^n-type)	Ry^* (S2)	−3	LP (N1)	Ry^* (C2)	−5
LP (N9, sp^n-type)	Ry^* (C10)	−12	LP (N1)	Ry^* (C6)	−4
LP (N9, sp^n-type)	σ^* (C1–S2)	−18	LP (N1)	Ry^* (C6)	−6
LP (N9, sp^n-type)	σ^* (C1–H17)	−3	LP (N1)	Ry^* (C7)	−9
LP (N9, sp^n-type)	σ^* (S2–C3)	−16	LP (N1)	σ^* (C2–C3)	−16
LP (N9, sp^n-type)	σ^* (S2–O14)	−26	LP (N1)	σ^* (C2–N5)	−8
LP (N9, sp^n-type)	σ^* (C10–C11)	−28	LP (N1)	π^* (C2–N5)	−114
LP (N9, sp^n-type)	σ^* (C10–N13)	−6	LP (N1)	σ^* (C6–H17)	−7
LP (N9, sp^n-type)	π^* (C10–N13)	−2	LP (N1)	σ^* (C6–H18)	−34
LP (N9, sp^n-type)	σ^* (N13–C28)	−2	LP (N1)	σ^* (C6–H19)	−5
LP (N9, sp^n-type)	σ^* (C28–H29)	−3	LP (N1)	σ^* (C7–H20)	−15
LP (N9, p-type)	Ry^* (S2)	−25	LP (N1)	σ^* (C7–H22)	−26
LP (N9, p-type)	Ry^* (S2)	−6			
LP (N9, p-type)	Ry^* (S2)	−4			
LP (N9, p-type)	Ry^* (C10)	−7			
LP (N9, p-type)	Ry^* (C10)	−3			
LP (N9, p-type)	σ^* (S2–C3)	−58			
LP (N9, p-type)	σ^* (S2–O14)	−65			
LP (N9, p-type)	π^* (C10–N13)	−193			

Table S3: Second order energies of the interactions of the two nitrogen lone pair NBOs in sulfoximine **2ac** and the nitrogen lone pair NBO in reference compound **2dc** with adjacent acceptor orbitals.

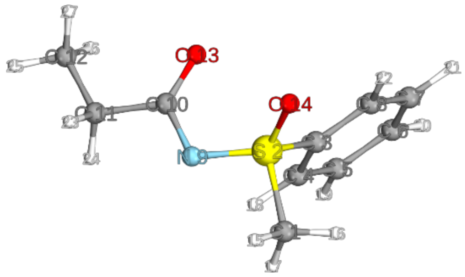

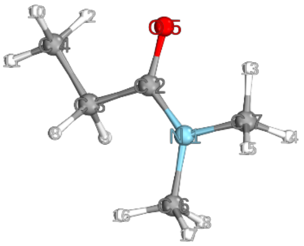

Donor orbital	Acceptor orbital	$E^{(2)}$ (kJ/mol)	Donor orbital	Acceptor orbital	$E^{(2)}$ (kJ/mol)
LP (N9, sp^n-type)	Ry^* (S2)	−20	LP (N1)	Ry^* (C2)	−4
LP (N9, sp^n-type)	Ry^* (C10)	−11	LP (N1)	Ry^* (C2)	−5
LP (N9, sp^n-type)	Ry^* (C10)	−7	LP (N1)	Ry^* (C2)	−3
LP (N9, sp^n-type)	σ^* (C1–S2)	−11	LP (N1)	Ry^* (C6)	−2
LP (N9, sp^n-type)	σ^* (C1–H16)	−3	LP (N1)	Ry^* (C6)	−7
LP (N9, sp^n-type)	σ^* (S2–C3)	−17	LP (N1)	Ry^* (C7)	−5
LP (N9, sp^n-type)	σ^* (S2–O14)	−14	LP (N1)	Ry^* (C7)	−3
LP (N9, sp^n-type)	σ^* (C10–O13)	−38	LP (N1)	σ^* (C2–O5)	−3
LP (N9, p-type)	Ry^* (S2)	−16	LP (N1)	π^* (C2–O5)	−267
LP (N9, p-type)	Ry^* (S2)	−12	LP (N1)	σ^* (C6–H16)	−5
LP (N9, p-type)	Ry^* (C10)	−9	LP (N1)	σ^* (C6–H17)	−9
LP (N9, p-type)	σ^* (S2–C3)	−42	LP (N1)	σ^* (C6–H18)	−30
LP (N9, p-type)	σ^* (S2–O14)	−63	LP (N1)	σ^* (C7–H14)	−26
LP (N9, p-type)	π^* (C10–O13)	−270	LP (N1)	σ^* (C7–H15)	−14

Table S4: Second order energies of the interactions of the two nitrogen lone pair NBOs in sulfoximines **2bc** and **2cc** with adjacent acceptor orbitals.

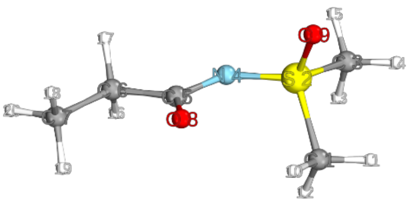

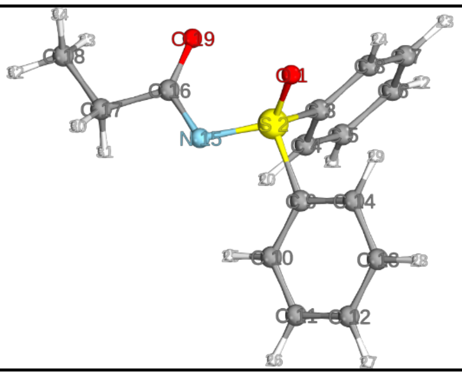

Donor orbital	Acceptor orbital	$E^{(2)}$ (kJ/mol)	Donor orbital	Acceptor orbital	$E^{(2)}$ (kJ/mol)
LP (N4, sp^n-type)	Ry^* (S2)	−21	LP (N15, sp^n-type)	Ry^* (S2)	−22
LP (N4, sp^n-type)	Ry^* (C5)	−11	LP (N15, sp^n-type)	Ry^* (S2)	−3
LP (N4, sp^n-type)	Ry^* (C5)	−2	LP (N15, sp^n-type)	Ry^* (C16)	−10
LP (N4, sp^n-type)	Ry^* (C5)	−6	LP (N15, sp^n-type)	Ry^* (C16)	−7

Table S4 **(continued):** Second order energies of the interactions of the two nitrogen lone pair NBOs in sulfoximines **2bc** and **2cc** with adjacent acceptor orbitals.

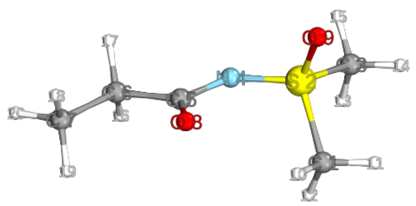

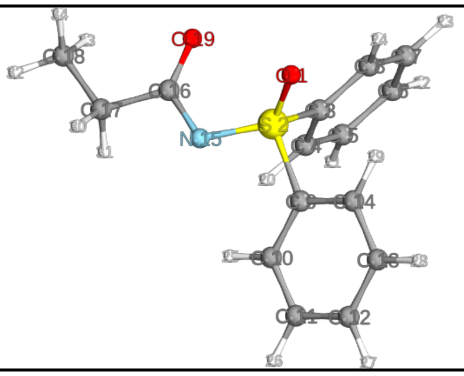

Donor orbital	Acceptor orbital	$E^{(2)}$ (kJ/mol)	Donor orbital	Acceptor orbital	$E^{(2)}$ (kJ/mol)
LP (N4, sp^n-type)	σ^* (C1–S2)	−20	LP (N15, sp^n-type)	σ^* (O1–S2)	−14
LP (N4, sp^n-type)	σ^* (S2–C3)	−11	LP (N15, sp^n-type)	σ^* (S2–C3)	−18
LP (N4, sp^n-type)	σ^* (S2–O9)	−12	LP (N15, sp^n-type)	σ^* (S2–C9)	−13
LP (N4, sp^n-type)	σ^* (C3–H14)	−3	LP (N15, sp^n-type)	σ^* (C16–C17)	−2
LP (N4, sp^n-type)	σ^* (C5–O8)	−39	LP (N15, sp^n-type)	σ^* (C16–O19)	−37
LP (N4, p-type)	Ry* (S2)	−16	LP (N15, p-type)	Ry* (S2)	−19
LP (N4, p-type)	Ry* (S2)	−13	LP (N15, p-type)	Ry* (S2)	−12
LP (N4, p-type)	Ry* (S2)	−2	LP (N15, p-type)	Ry* (S2)	−3
LP (N4, p-type)	Ry* (C5)	−10	LP (N15, p-type)	Ry* (C16)	−9
LP (N4, p-type)	σ^* (C1–S2)	−39	LP (N15, p-type)	σ^* (O1–S2)	−65
LP (N4, p-type)	σ^* (S2–O9)	−64	LP (N15, p-type)	σ^* (S2–C3)	−41
LP (N4, p-type)	π^* (C5–O8)	−281	LP (N15, p-type)	π^* (C16–O19)	−262

3 IBO analysis—Calculation of orbital change in a hypothetical isomerization reaction

For the hypothetical isomerization of *N*-2-oxopropyl-*S*-methyl-*S*-phenyl sulfoximine (**3a**) to *N*-propionyl-*S*-methyl-*S*-phenyl sulfoximine (**2ac**) shown in Scheme S1, the changes in all intrinsic bond orbitals were computed. This was done as described in reference S34 with some modifications. As described in reference S34, the IAO partial charges on each atom associated with each bond *i* are summarized in a vector $q^i = (q_{\text{atom1}}, q_{\text{atom2}}, q_{\text{atom3}}, \ldots, q_{\text{atom}N})^i$ (see Equation S4). The change in orbital *i* can then be computed by computing the latter vector for the educt and the product and by calculating the norm ("taxi-cab norm") of the difference of the two vectors $\lVert q^i(\text{product}) - q^i(\text{educt}) \rVert$.[S34] When attempting to perform this assessment for the reaction in Scheme S1 with the numbering of atoms shown in Figure S4 and Figure S5, it becomes evident, however, that some rather significant orbital changes are computed only because certain bonds change their position. For example, while the oxygen atom O13 is bound to the carbon atom C11 in the educt the same oxygen is bound to the carbon atom C10 in the product. The corresponding change in the atomic position from carbon C11 to carbon C10 results in a significant orbital change for the C–O π-bond which, however, does not reflect the relatively small change of the IAO partial charge on carbon. To account for the fact that in the latter cases the IAO partial charge on carbon C10 must be compared with the IAO partial charge on carbon C11, the slightly modified scheme described by Equation S5, Equation S6 and Equation S7 was employed to calculate the IBO change in the hypothetical isomerization shown in Scheme S1.

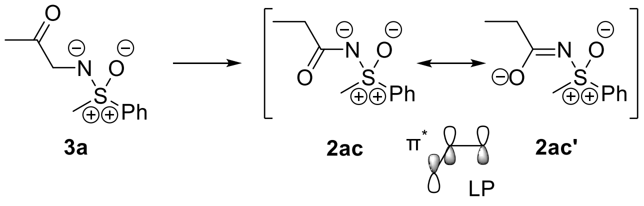

Scheme S1. Hypothetical isomerization of *N*-2-oxopropyl-*S*-methyl-*S*-phenyl sulfoximine (**3a**) to the *N*-propionyl congener **2ac**.

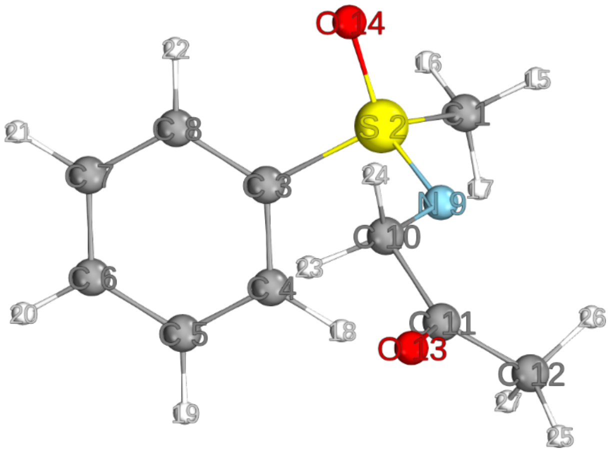

Figure S4. Labelling of atoms in *N*-2-oxopropyl-*S*-methyl-*S*-phenyl sulfoximine (**3a**).

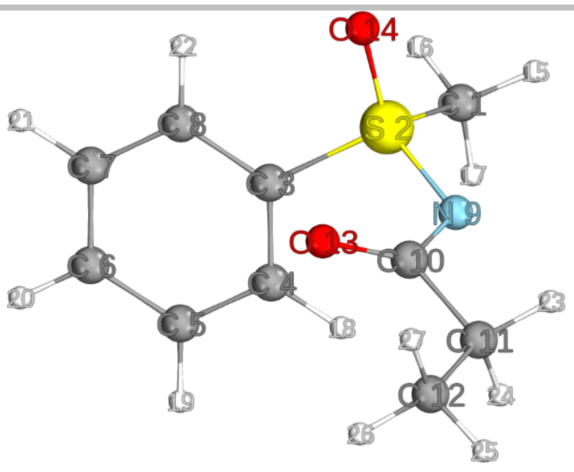

Figure S5. Labelling of atoms in *N*-propionyl-*S*-methyl-*S*-phenyl sulfoximine (**2ac**).

$$\vec{q}^{\,i} = \left(q^i_{\mathrm{atom1}}, q^i_{\mathrm{atom2}}, q^i_{\mathrm{atom3}}, \dots, q^i_{\mathrm{atomN}}\right)^T$$ **Equation S4**

$$B = \{\sigma_{\mathrm{C-H23}}, \sigma_{\mathrm{C-H24}}, \sigma_{\mathrm{C-O13}}, \pi_{\mathrm{C-O13}}, \mathrm{LP}_{\mathrm{O13,\,p\text{-}type}}\}$$ **Equation S5**

$$\Delta q^i = \begin{cases} \left\|\vec{q}^{\,i}(\mathrm{product}) - \vec{q}^{\,i}(\mathrm{educt})\right\| = \sum_j^{\mathrm{atoms}} \left|q^i_j(\mathrm{product}) - q^i_j(\mathrm{educt})\right| & (i \notin B), \\ \sum_j^{\mathrm{atoms}} \left|q^i_j(\mathrm{product}) - q^i_{f(j)}(\mathrm{educt})\right| & (i \in B) \end{cases}$$ **Equation S6**

$$f(j) = \begin{cases} \mathrm{C11} & (j = \mathrm{C10}), \\ \mathrm{C10} & (j = \mathrm{C11}), \\ j & (j \neq \mathrm{C10}, \mathrm{C11}) \end{cases}$$ **Equation S7**

q^i_j = IAO partial charge on atom *j* associated with bond *i* (C)

Δq^i = Orbital change associated with bond *i* in the hypothetical isomerization depicted in Scheme S1 (C)

4 Torsional potential and Lewis and delocalization energy contributions to the potential (Figure S6)—Supplemental results

Figure S6 includes supplemental delocalization energy contributions to the S–N–C–O torsional potential of *N*-propionyl-*S*-methyl-*S*-phenyl sulfoximine (**2ac**) which were not included in Figure 6 in the manuscript. Figures S6C to S6E show the torsional dependencies of the donor-acceptor interactions which delocalize electron density from the sulfoximidoyl fragment to the propionyl group and vice versa. These interactions are expected to vary significantly with changes in ϕ(S–N–C–O). Additionally, Figure S6F includes the delocalization energies for the interactions of the sulfoximidoyl nitrogen lone pairs with the S–CH_3 and S–O σ^*-orbitals since these were also found to vary significantly with ϕ(S–N–C–O). Interactions with Rydberg orbitals are not shown in Figures S6C to S6F since they showed negligible torsional variations.

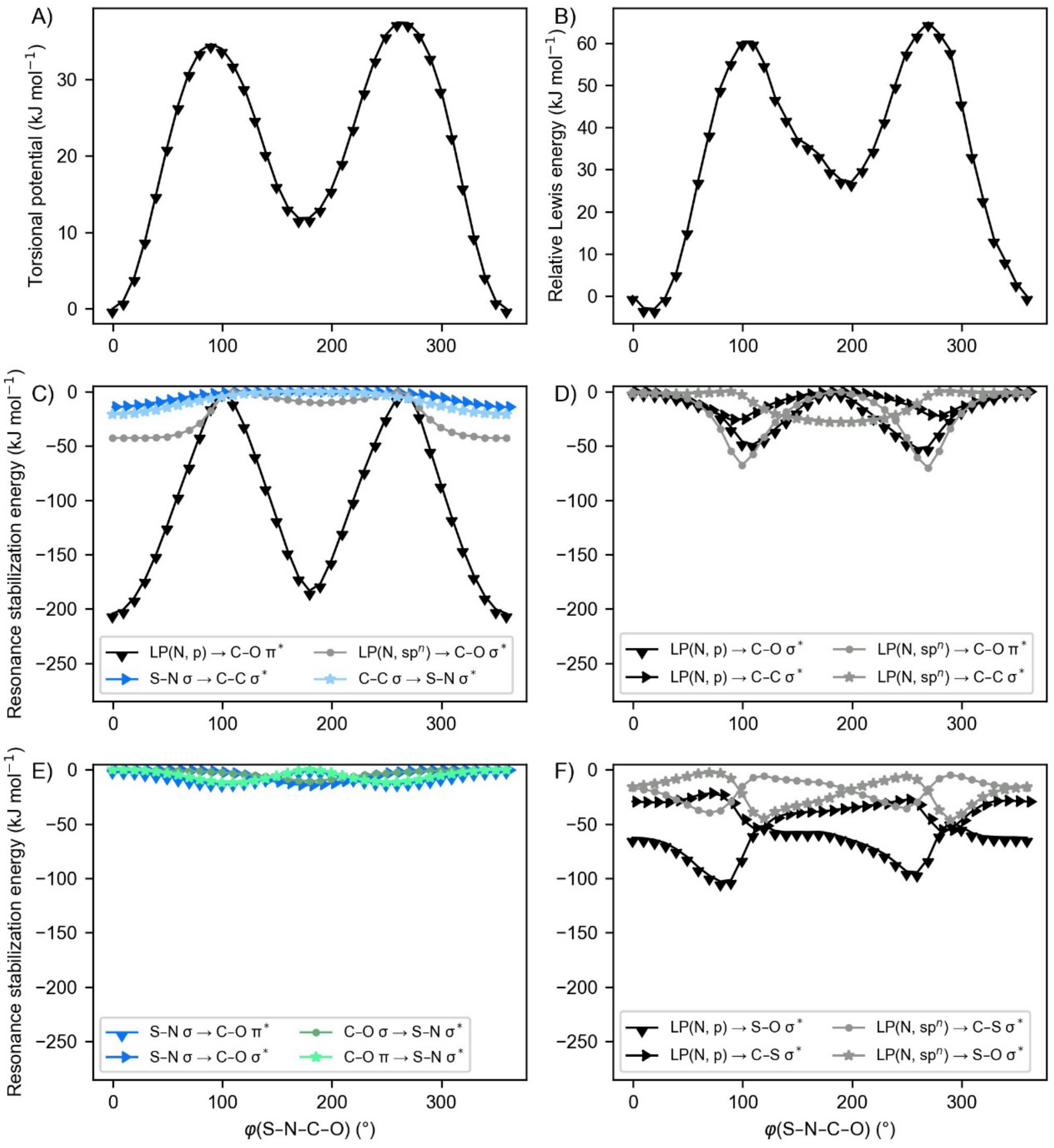

Figure S6. Torsional dependencies of the potential, relative Lewis energy contribution and resonance stabilization energies, computed at the B3LYP-D3/6-311++G(d,p)//r2SCAN-3c/def2-mTZVPP+SMD(DCM) level of theory. A) Torsional potential. B) Torsional dependence of the relative Lewis energy. C) Torsional dependencies of the delocalization energy contributions which exhibit global minima at ϕ(S–N–C–O) = 0°. D) Torsional dependencies of the delocalization energy contributions which show significant variations with ϕ(S–N–C–O) but do not exhibit global minima at ϕ(S–N–C–O) = 0°. E) Torsional dependencies of delocalization energy contributions which show little ϕ-dependent change and do not exhibit a global minimum at ϕ(S–N–C–O) = 0°. F) Torsional dependencies of the interactions of the nitrogen lone pairs with the S–CH_3 and S–O σ^*-bonds.

5 Mechanistic studies—Results

5.1 Addition of benzoic acid (10) to *N*-alkynylated sulfoximine 9

5.1.1 Energies, thermostatistical corrections and Gibbs energies

The Gibbs energy profiles for the DFT assessed transformations of *N*-alkynylated sulfoximine **9** with benzoic acid (**10**) are shown in Figure S7. The single point energies, thermostatistical corrections and Gibbs energies of all structures are summarized in Table S5.

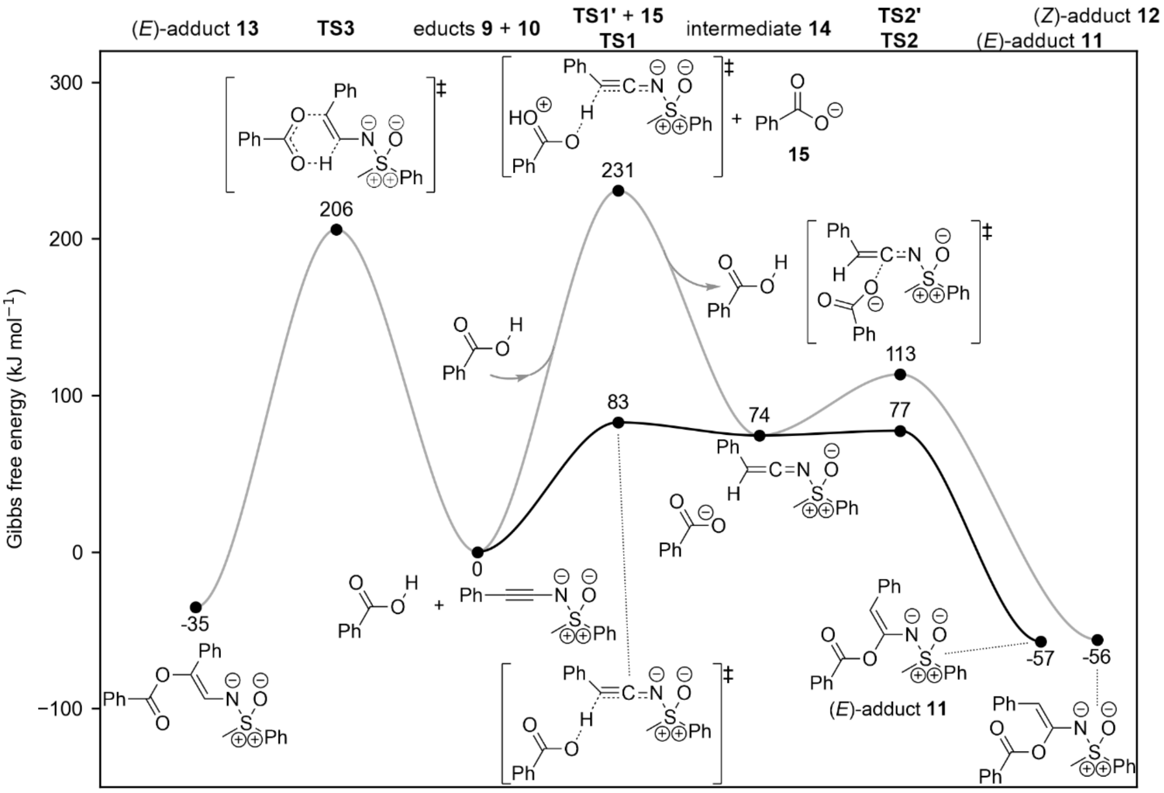

Figure S7. Gibbs energy profiles computed at the ωB97M-V/def2-QZVP//PBE0-D3/def2-SVP+SMD(THF) level of theory for the reactions of *N*-alkynylated sulfoximine **9** with benzoic acid (**10**), leading to the adducts **11** (experimentally observed), and **12** and **13** (not observed experimentally). Gibbs energies were computed at a temperature of 298.15 K and for a concentration of 1 mol/L in solution.

Table S5: Gibbs energies of all stationary points which were obtained for the reactions of *N*-alkynylated sulfoximine **9** with benzoic acid (**10**), leading to adducts **11** (experimentally observed), and **12** and **13** (not observed experimentally).

Structure	$E^{\omega B97M\text{-}V}$ (hartree)[a]	$G^{PBE0} - E^{PBE0}$ (hartree)[b]	$G = E^{\omega B97M\text{-}V} + (G^{PBE0} - E^{PBE0}) + k_B T \ln(V_m)$ (hartree)[c]	ΔG (kJ/mol)	imaginary frequencies
sulfoximine **9**	−1107.53691132	0.19898908	−1107.334903	-	0
benzoic acid **10**	−420.88366595	0.08509138	−420.795556	-	0
educts **9** + **10**	−1528.42057728	0.28408046	−1528.130459	0	-
**TS1**	−1528.40411068	0.30214108	−1528.098951	83	1
intermediate **14**	−1528.41163109	0.30647221	−1528.102140	74	0
**TS2**	−1528.41141877	0.30742780	−1528.100972	77	1

***Table S5* (continued):** Gibbs energies of all stationary points which were obtained for the reactions of *N*-alkynylated sulfoximine **9** with benzoic acid (**10**), leading to adducts **11** (experimentally observed), and **12** and **13** (not observed experimentally).

Structure	$E^{\omega B97M\text{-}V}$ (hartree)[a]	$G^{PBE0} - E^{PBE0}$ (hartree)[b]	$G = E^{\omega B97M\text{-}V} + (G^{PBE0} - E^{PBE0}) + k_B T \ln(V_m)$ (hartree)[c]	ΔG (kJ/mol)	imaginary frequencies
(*E*)-adduct **11**	−1528.46725408	0.31199078	−1528.152244	−57	0
**TS2'**	−1528.39758188	0.30730410	−1528.087259	113	1
(*Z*)-adduct **12**	−1528.46693332	0.31214906	−1528.151765	−56	0
**TS3**	−1528.35917485	0.30412474	−1528.052031	206	1
(*E*)-adduct **13**	−1528.45780942	0.31088760	−1528.143903	−35	0
benzoate anion **15**	−420.40028627	0.07173337	−420.325534	-	0
**TS1'**	−1528.83409956	0.31848766	−1528.512593	-	1
**TS1'** + **15**	−1949.23438583	0.39022103	−1948.838127	231[d]	-

[a] Single-point energy computed at the ωB97M-V/def2-QZVP//PBE0-D3/def2-SVP+SMD(THF) level of theory. The Gibbs energy of solvation and the dispersion correction are already included in the reported energies. [b] Thermostatistical contributions to the Gibbs energy computed at the PBE0-D3/def2-SVP+SMD(THF) level of theory. The zero-point contributions, the volume work term to the enthalpy and all contributions to the inner energy and the entropy due to the thermal population of translational, rotational and vibrational states are included in the thermostatistical corrections. [c] Gibbs energy at a concentration of 1 mol/L in solution and a temperature of 298.15 K. [d] Gibbs energy relative to the Gibbs energy of *N*-alkynylated sulfoximine **9** and two molecules of benzoic acid (**10**).

5.1.2 Kinetic analysis (Figure 11, Figure S7)

A kinetic analysis for the reaction of *N*-alkynylated sulfoximine **9** with benzoic acid (**10**) leading to (*E*)-adduct **11** was conducted by assuming bimolecular, irreversible, second order reaction kinetics (A + B → P). Considering the DFT computed Gibbs energy profile in Figure S7, this was considered a reasonable assumption. For a bimolecular, irreversible, second order reaction, the integrated rate law is given by Equation S8, wherein the rate constant can be quantified by the Eyring formula in Equation S9 (in the latter formula a transmission coefficient of unity was assumed).[S35,S36] Furthermore, the relationships in Equation S10 and Equation S11 apply. Combining Equations S8 to S11 and rearranging provides Equation S12. Employing the initial reactant concentrations and the reaction time reported in reference S37, and the DFT computed Gibbs energy barriers of the rate-limiting step at 273.15 K and 298.15 K ($[A]_0$ = 0.2 mol/L, $[B]_0$ = 0.3 mol/L, t = 1 h, $\Delta G^\ddagger$ = 78.2 kJ/mol (273.15 K), $\Delta G^\ddagger$ = 82.7 kJ/mol (298.15 K)), yields of 96% (273.15 K) and 100% (298.15 K) were calculated from Equation S12. The computed yields are in reasonable agreement with the experimentally determined yields of 83% (273.15 K) and 96% (298.15 K).[S37]

$$\ln\left(\frac{[A]_0[B]}{[A][B]_0}\right) = k([B]_0 - [A]_0)t$$ **Equation S8**

$$k = \frac{k_\mathrm{B}T}{h \cdot 1\frac{\mathrm{mol}}{\mathrm{L}}}\exp\left(-\frac{\Delta G^\ddagger}{RT}\right)$$ **Equation S9**

$$[A] = [A]_0 - Y[A]_0$$ **Equation S10**

$$[B] = [B]_0 - Y[A]_0$$ **Equation S11**

$$Y = \frac{[B]_0\left(1 - \exp\left(\frac{([B]_0 - [A]_0)tk_BT}{h \cdot 1\frac{\mathrm{mol}}{\mathrm{L}}}\exp\left(-\frac{\Delta G^\ddagger}{RT}\right)\right)\right)}{[A]_0 - [B]_0\exp\left(\frac{([B]_0 - [A]_0)tk_BT}{h \cdot 1\frac{\mathrm{mol}}{\mathrm{L}}}\exp\left(-\frac{\Delta G^\ddagger}{RT}\right)\right)}$$ **Equation S12**

$[A]$ = Concentration of the limiting reactant A ($\mathrm{mol \cdot L^{-1}}$)
$[A]_0$ = Initial concentration of the limiting reactant A ($\mathrm{mol \cdot L^{-1}}$)
$[B]$ = Concentration of reactant B ($\mathrm{mol \cdot L^{-1}}$)
$[B]_0$ = Initial concentration of reactant B ($\mathrm{mol \cdot L^{-1}}$)
k = Rate constant ($\mathrm{L \cdot mol^{-1} \cdot s^{-1}}$)
t = Time (s)
k_B = Boltzmann constant ($\mathrm{J \cdot K^{-1}}$)
T = Temperature (K)
h = Planck constant (J·s)
$\Delta G^\ddagger$ = Gibbs free energy barrier of the reaction ($\mathrm{J \cdot mol^{-1}}$)
R = Ideal gas constant ($\mathrm{J \cdot K^{-1} \cdot mol^{-1}}$)
Y = Yield ()

5.1.3 Relaxed surface scan—Concerted formation of (*E*)-adduct 11

To provide additional proof for the sequential nature of the addition of benzoic acid (**10**) to *N*-alkynylated sulfoximine **9** under formation of (*E*)-adduct **11**, a relaxed surface scan for a concerted process was performed at the PBE0-D3/def2-SVP+SMD(THF) level of theory (SCF convergence threshold = VeryTightSCF, numerical grid = DEFGRID3). Thereby, the distances *d*(C10–O11) and *d*(C20–H41) in Figure S8 were constrained in each step of the scan to identical values. The scan was performed up to a distance of 1.40 Å, which is very close to the C10–O11 bond length in product **11**. The results of the relaxed surface scan are summarized in Figure S9 and are compared to the relative energy of the first, rate-limiting transition state **TS1** in the sequential addition of benzoic acid (**10**) to *N*-alkynylated sulfoximine **9**. As can be observed from Figure S9, the concerted process proceeds via a significantly higher energy barrier than the sequential mechanism, indicating that the addition of benzoic acid occurs via a two-step pathway.

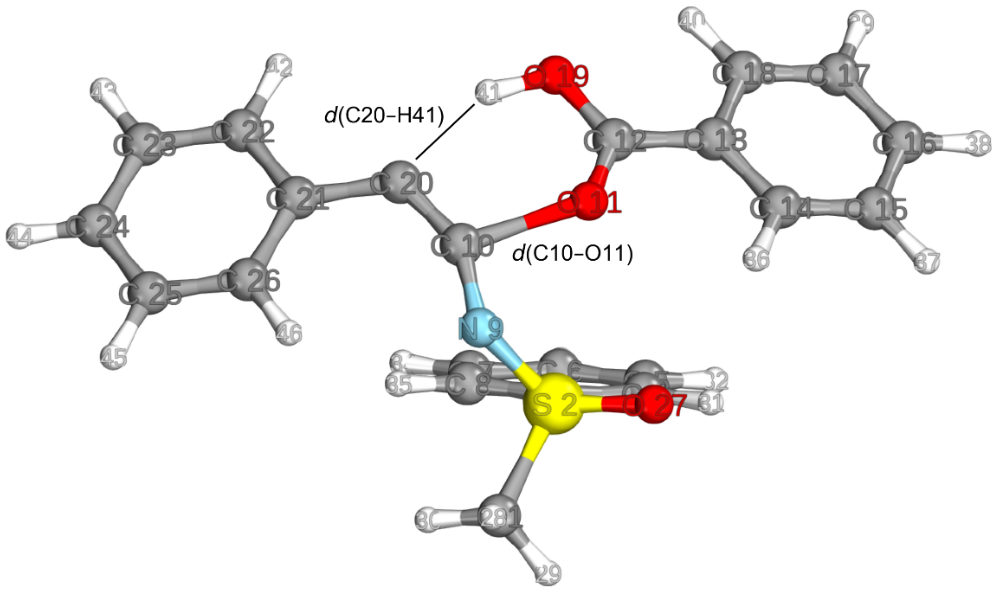

Figure S8. The scan coordinates *d*(C10–O11) and *d*(C20–H41) in the relaxed surface scan for the concerted addition of benzoic acid (**10**) to *N*-alkynylated sulfoximine **9**.

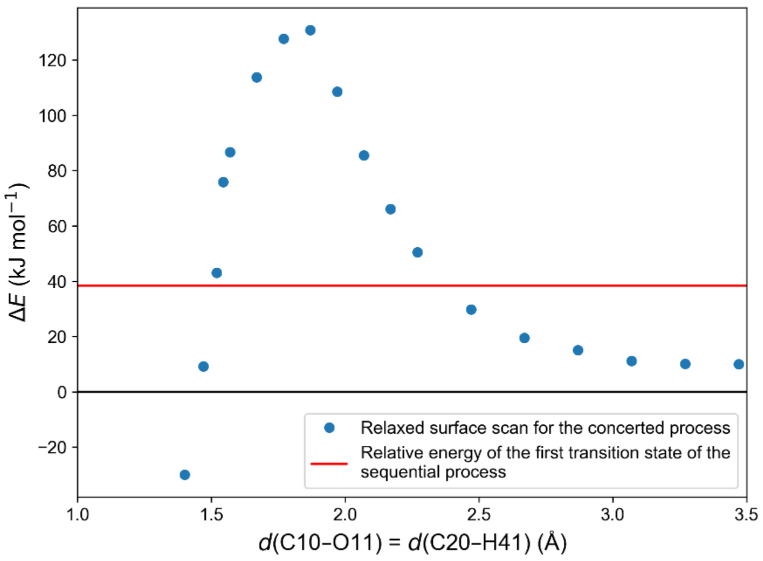

Figure S9. Relative energies from the relaxed surface scan for the concerted addition of benzoic acid (**10**) to *N*-alkynylated sulfoximine **9** (blue dots), which are compared to the relative energy of the first, rate-limiting transition state (**TS1**) of the sequential process (red line). All energies were obtained at the PBE0-D3/def2-SVP+SMD(THF) level of theory (SCF convergence threshold = VeryTightSCF, numerical grid = DEFGRID3). The scan coordinates are defined in Figure S8.

5.1.4 Relaxed surface scan—Formation of alternative (*E*)-adduct 13 by consecutive proton transfer and C–O bond formation

A process in which alternative (*E*)-adduct **13** is formed by a consecutive proton transfer and C–O bond formation from *N*-alkynylated sulfoximine **9** and benzoic acid (**10**) was investigated with relaxed surface scans at the PBE0-D3/def2-SVP+SMD(THF) level of theory (SCF convergence threshold = VeryTightSCF, numerical grid = DEFGRID3). In Figure S10, the results from the first scan are shown in which the initial proton transfer was evaluated. The proton transfer from benzoic acid (**10**) to the alkynic carbon atom in α-position to the sulfoximidoyl nitrogen of *N*-alkynylated sulfoximine **9** was evaluated at an angle θ(C20–C10–O19) = 90°, which was constrained during the scan (see Figure S11). To ensure a consecutive process, the dihedral angle φ(C20–C10–O19–C12) was additionally constrained at 90° (this prevents the carbonyl oxygen atom from stabilizing the hole which is formed in β-position to the sulfoximidoyl nitrogen). As can be observed from Figure S10, the energy increases monotonically upon proton transfer and no minimum is observed. The distance d(C10–H41) = 1.099 Å in Figure S10 at which the final energy evaluation was performed corresponds to the equilibrium C10–H41 distance in the optimized structure of (*E*)-adduct **13**. The structures from the relaxed surface scan before and after the proton transfer are shown in Figure S11.

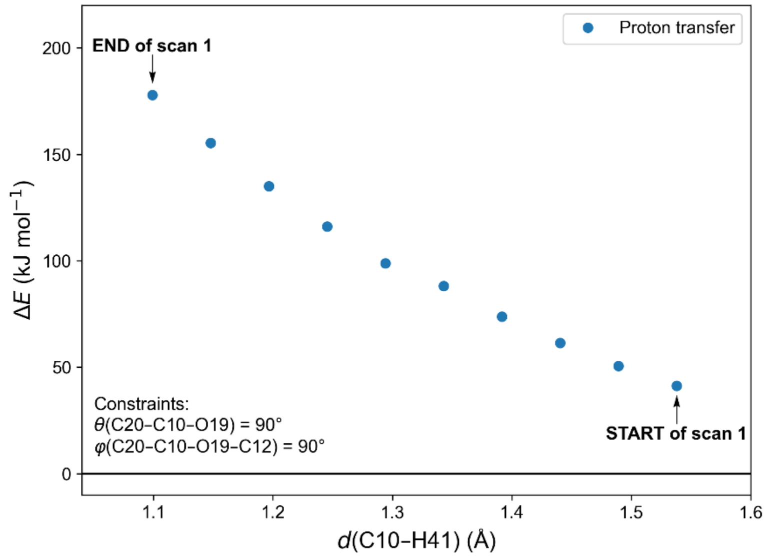

Figure S10. Results from the relaxed surface scan for the proton transfer from benzoic acid (**10**) to *N*-alkynylated sulfoximine **9**.

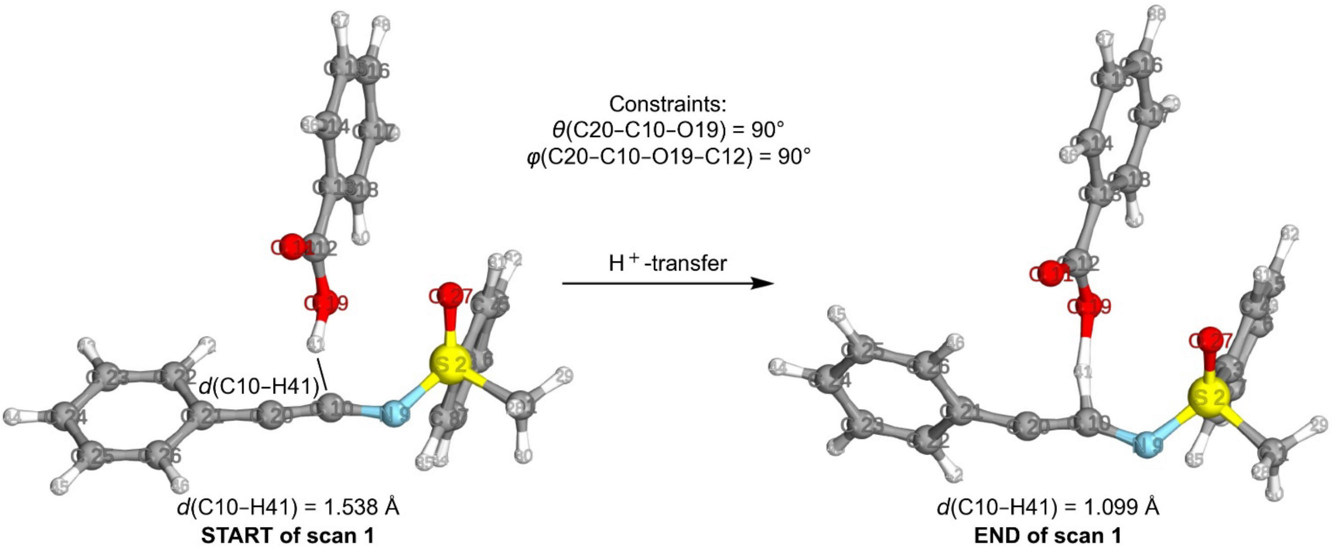

Figure S11. Structures from the relaxed surface scan before and after the proton transfer from benzoic acid (**10**) to *N*-alkynylated sulfoximine **9**.

Subsequently, the C–O bond formation was studied by adjusting the angle θ_1(C20–C10–O19), which was constrained at 90° in the first scan, in a stepwise manner from 90.0° to 33.1°. The angle of 33.1° corresponds to the equilibrium angle in the optimized structure of product **13**. The distance *d*(C10–H41), which was adjusted in the first scan, was constrained to the equilibrium C10–H41 bond length in (*E*)-adduct **13**, i.e. *d*(C10–H41) = 1.099 Å. The dihedral angle φ(C20–C10–O19–C12) maintained at 90° in the first scan was constrained to the same value in the second scan. The angle θ_2(C20–C10–H41) was also fixed but was considered an optimizable parameter. The optimization was conducted by performing four relaxed surface scans with different, fixed values of θ_2, starting with θ_2 = 84.1°, the value obtained at the end of scan one, and ending with θ_2 = 115.4°, the angle found in the optimized geometry of (*E*)-adduct **13**. Figure S12 contains the results from these scans. As can be observed from Figure S12, the angle θ_2(C20–C10–H41) rapidly relaxes upon C–O bond formation to an angle of 115.4°. The minimum energy path from Figure S12 is shown separately in Figure S13 and is compared to the energy of the optimized transition state **TS3** of the concerted, nonconsecutive process (see section 5.1.1). The structures before and after the C–O bond formation are shown in Figure S14. In contrast to the proton transfer in Figure S11 which is uphill in energy (see Figure S10), the C–O bond formation in Figure S14 eventually leads to an energy decrease (see Figure S13). Compared to the consecutive process (proton transfer first then C–O bond formation), transition state **TS3** of the nonconsecutive process is lower in energy (see Figure S13). Hence, the nonconsecutive process benefits from the energetically favorable C–O bond forming process, which occurs concurrently with the proton transfer.

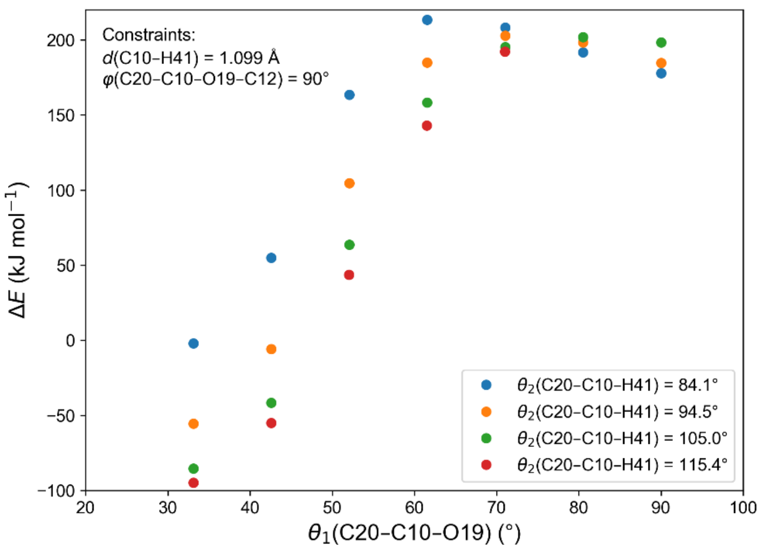

Figure S12. Optimization of the angle θ_2 relative to the angle θ_1.

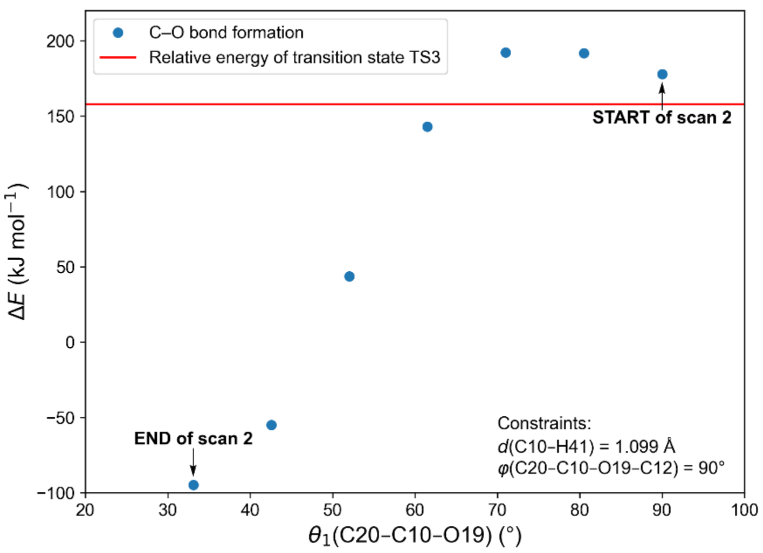

Figure S13. Comparision of the minimum energy pathway for C–O bond formation in the consecutive process with the energy of transition state **TS3**.

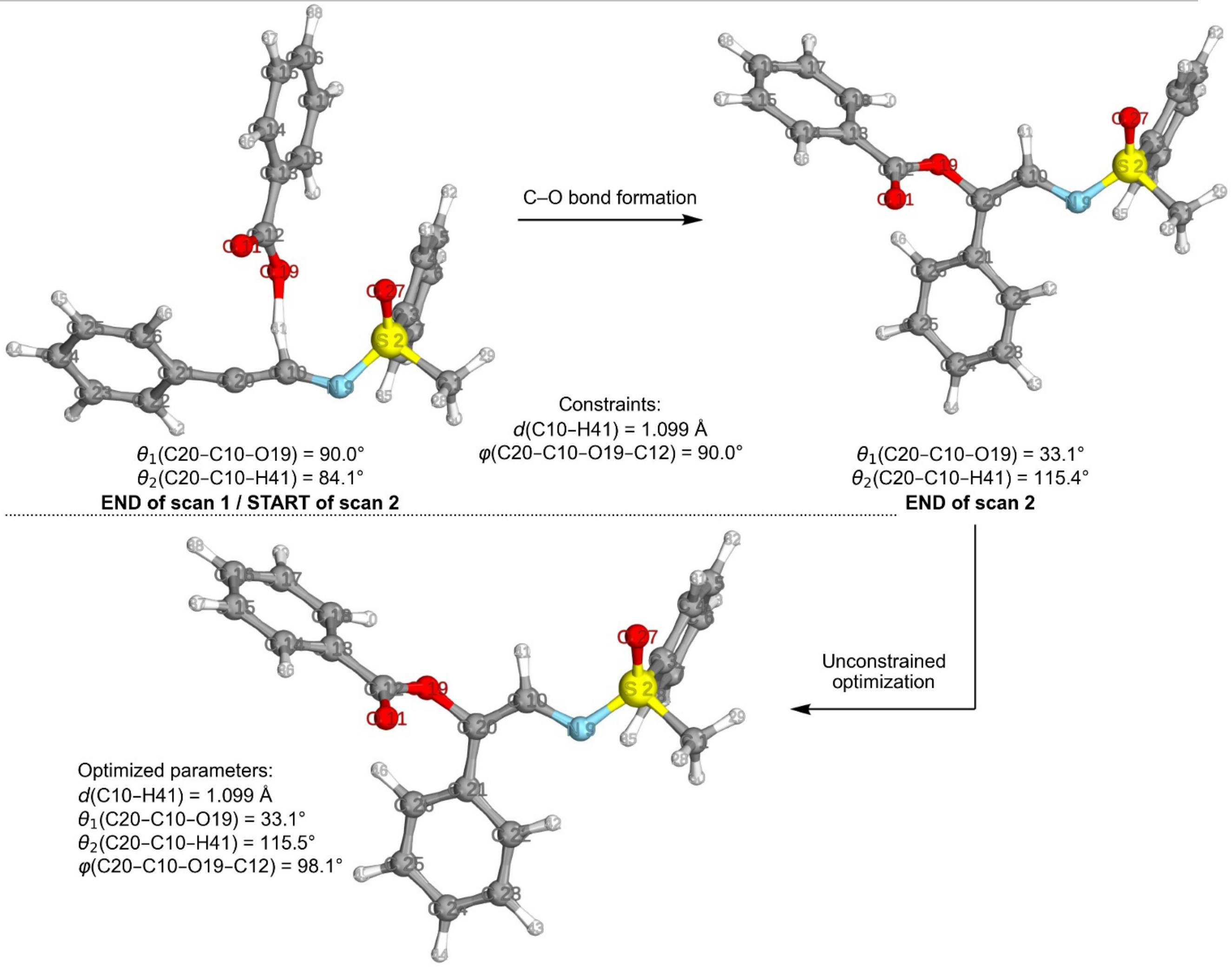

Figure S14. Structures from scan two, and optimized structure of (*E*)-adduct **13**.

5.1.5 Effect of different donors on the Gibbs energy barrier of the benzoic acid addition

The effect of different substituents on the reaction barrier of the benzoic acid addition was studied by replacing the sulfoximidoyl group in compound **9** by a hydrogen atom and two alternative donors (D = –OMe, –NMe_2). The results of these assessments are summarized in Figure S15 and Table S6. As can be observed from Figure S15, concerted reaction pathways were found for the additions to all three derivatives of compound **9**. Compared to phenylacetylene (**23**), the computed barriers are significantly reduced for alkynes **9**, **25** and **27**, which are substituted with mesomeric donors.

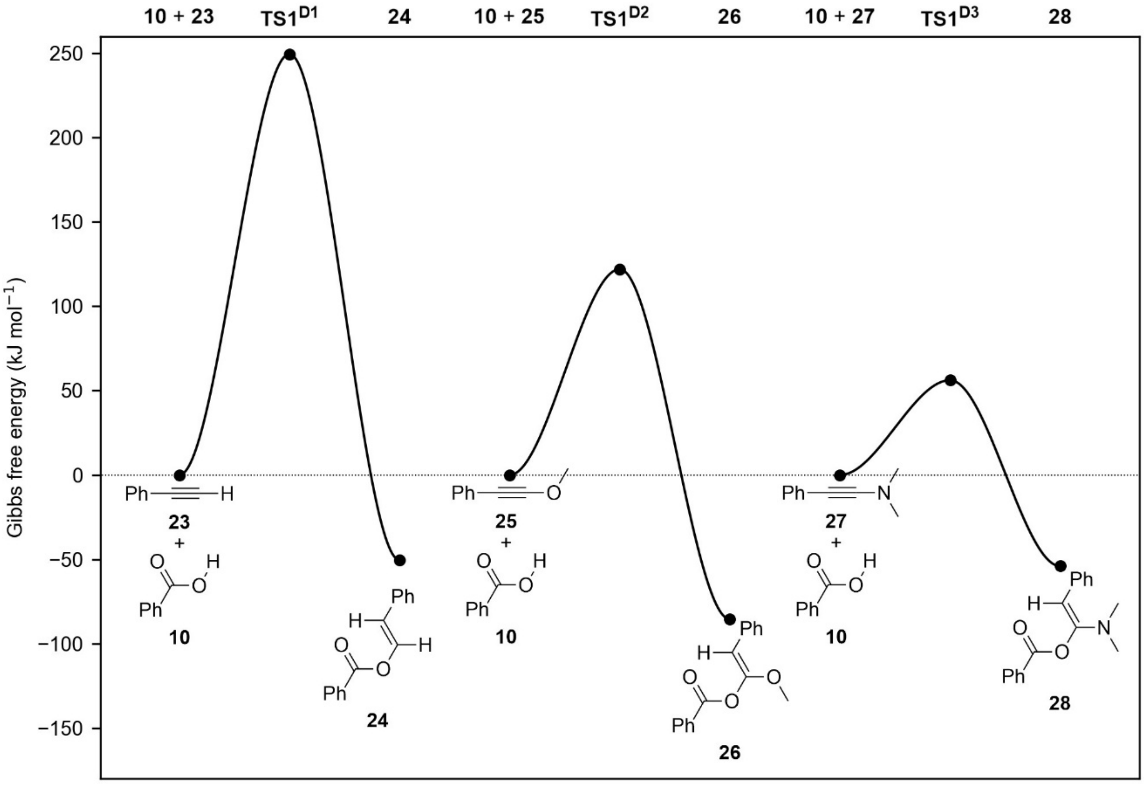

Figure S15. Gibbs energy profiles for the additions of benzoic acid (**10**) to acetylenes **23**, **25** and **27** with different, alkyne-bound groups (H, OMe, NMe_2) assessed at the ωB97M-V/def2-QZVP//PBE0-D3/def2-SVP+SMD(THF) level of theory. Gibbs energies were computed at a temperature of 298.15 K and for a concentration of 1 mol/L in solution.

Table S6: Gibbs energies for the additions of benzoic acid (**10**) to acetylenes **23**, **25** and **27** with different, alkyne-bound groups (H, OMe, NMe_2).

Structure	$E^{\omega B97M-V}$ (hartree)[a]	$G^{PBE0} - E^{PBE0}$ (hartree)[b]	$G = E^{\omega B97M-V} + (G^{PBE0} - E^{PBE0}) + k_B T \ln(V_m)$ (hartree)[c]	ΔG (kJ/mol)	imaginary frequencies
benzoic acid **10**	−420.88366595	0.08509138	−420.795556	-	0
alkyne **23**	−308.40623404	0.08008677	−308.323128	-	0
educts **10** + **23**	−729.28989999	0.16517815	−729.118684	0	-
**TS1^{D1}**	−729.20670444	0.18002831	−729.023657	249	1
product **24**	−729.33135959	0.19044925	−729.137892	−50	0
alkyne **25**	−422.93200030	0.10920945	−422.819772	-	0
educts **10** + **25**	−843.81566625	0.19430083	−843.615328	0	-
**TS1^{D2}**	−843.78373129	0.21184188	−843.568871	122	1
product **26**	−843.87129074	0.22038746	−843.647884	−85	0
alkyne **27**	−442.37011773	0.14737091	−442.219728	-	0

***Table S6* (continued):** Gibbs energies for the additions of benzoic acid (**10**) to acetylenes **23**, **25** and **27** with different, alkyne-bound groups (H, OMe, NMe_2).

Structure	$E^{\omega B97M\text{-}V}$ (hartree)[a]	$G^{PBE0} - E^{PBE0}$ (hartree)[b]	$G = E^{\omega B97M\text{-}V} + (G^{PBE0} - E^{PBE0}) + k_B T \ln(V_m)$ (hartree)[c]	ΔG (kJ/mol)	imaginary frequencies
educts **10** + **27**	−863.25378368	0.23246229	−863.015284	0	-
**TS1^{D3}**	−863.25062584	0.25371287	−862.993894	56	1
product **28**	−863.29793845	0.25905658	−863.035863	−54	0

[a] Single-point energy computed at the ωB97M-V/def2-QZVP//PBE0-D3/def2-SVP+SMD(THF) level of theory. The Gibbs energy of solvation and the dispersion correction are already included in the reported energies. [b] Thermostatistical contributions to the Gibbs energy computed at the PBE0-D3/def2-SVP+SMD(THF) level of theory. The zero-point contributions, the volume work term to the enthalpy and all contributions to the inner energy and the entropy due to the thermal population of translational, rotational and vibrational states are included in the thermostatistical corrections. [c] Gibbs energy at a concentration of 1 mol/L in solution and a temperature of 298.15 K.

5.2 Cyclization of *N*-alkynylated sulfoximine 9 with isobutyryl chloride (16)

5.2.1 Energies, thermostatistical corrections and Gibbs energies for the lowest Gibbs energy pathway

The Gibbs energy profile for the in-situ formation of ketene **21** from isobutyryl chloride (**16**) and subsequent cyclization with *N*-alkynylated sulfoximine **9** to product **17** is shown in Figure S16. The single point energies, thermostatistical corrections and Gibbs energies of all structures are summarized in Table S7.

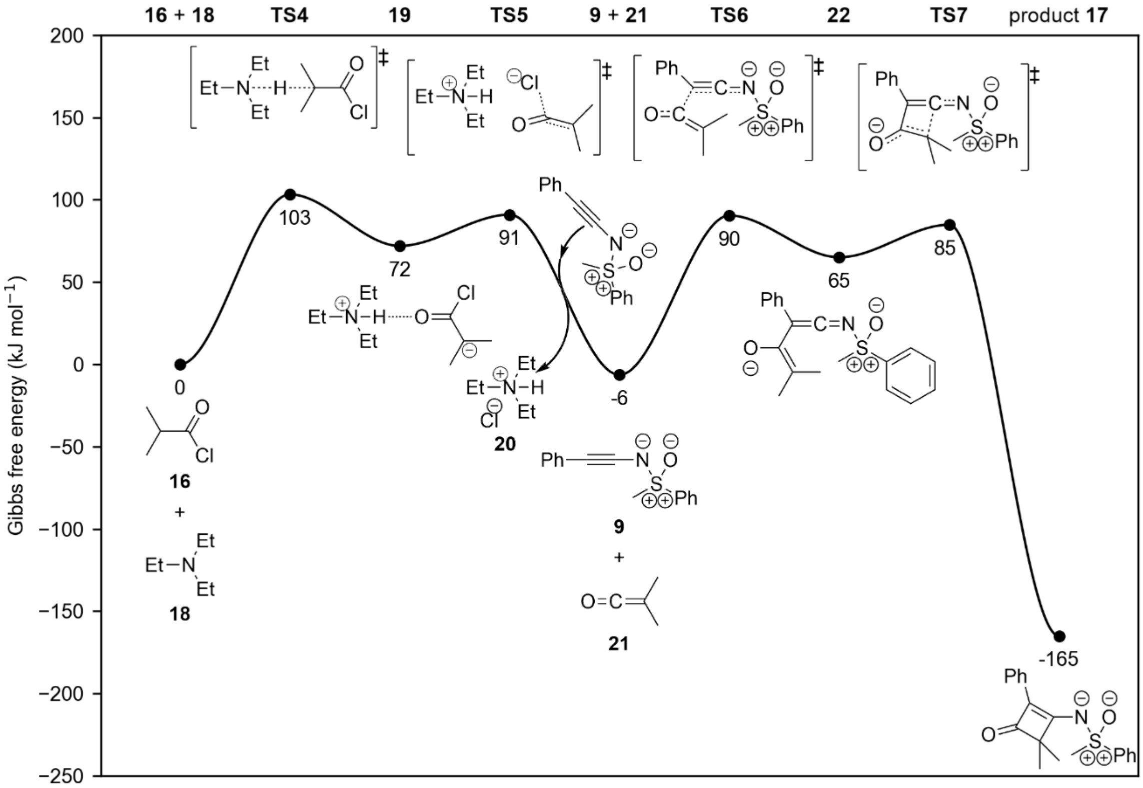

Figure S16. Gibbs energy profile for the in-situ formation of ketene **21** from isobutyryl chloride (**16**) and the subsequent cyclization with *N*-alkynylated sulfoximine **9** to product **17** assessed at the ωB97M-V/def2-QZVP//PBE0-D3/def2-SVP+SMD(DCM) level of theory. Gibbs energies were computed at a temperature of 313.15 K and for a concentration of 1 mol/L in solution.

***Table S7*:** Gibbs energies for the in-situ formation of ketene **21** from isobutyryl chloride (**16**) and the subsequent cyclization with *N*-alkynylated sulfoximine **9** to product **17**.

Structure	$E^{\omega B97M-V}$ (hartree)[a]	$G^{PBE0} - E^{PBE0}$ (hartree)[b]	$G = E^{\omega B97M-V} + (G^{PBE0} - E^{PBE0}) + k_B T \ln(V_m)$ (hartree)[c]	ΔG (kJ/mol)	imaginary frequencies
sulfoximine **9**	−1107.54065719	0.19617308	−1107.341265	-	0
**16**	−692.09418963	0.07104717	−692.019923	-	0
amine **18**	−292.37738381	0.16977769	−292.204387	-	0
**9** + **16** + **18**	−2092.01223064	0.43699794	−2091.565575	0	-
**TS4**	−984.45017893	0.26195433	−984.185005	-	1
**9** + **TS4**	−2091.99083612	0.45812741	−2091.526270	103	-
**19**	−984.46463448	0.26450337	−984.196912	-	0

***Table S7* (continued):** Gibbs energies for the in-situ formation of ketene **21** from isobutyryl chloride (**16**) and the subsequent cyclization with *N*-alkynylated sulfoximine **9** to product **17**.

Structure	$E^{\omega B97M\text{-}V}$ (hartree)[a]	$G^{PBE0} - E^{PBE0}$ (hartree)[b]	$G = E^{\omega B97M\text{-}V} + (G^{PBE0} - E^{PBE0}) + k_B T \ln(V_m)$ (hartree)[c]	ΔG (kJ/mol)	imaginary frequencies
**9 + 19**	−2092.00529167	0.46067645	−2091.538177	72	-
**TS5**	−984.45884570	0.26593909	−984.189687	-	1
**9** + **TS5**	−2091.99950289	0.46211217	−2091.530952	91	-
**20**	−753.23564795	0.18189916	−753.050529	-	0
ketene **21**	−231.23844340	0.05900687	−231.176217	-	0
**9** + **20** + **21**	−2092.01474854	0.43707911	−2091.568011	−6	-
**TS6**	−1338.76289288	0.27905294	−1338.480621	-	1
**20** + **TS6**	−2091.99854083	0.46095210	−2091.531150	90	-
**22**	−1338.77550120	0.28199428	−1338.490288	-	0
**20** + **22**	−2092.01114914	0.46389344	−2091.540817	65	-
**TS7**	−1338.76948179	0.28351250	−1338.482750	-	1
**20** + **TS7**	−2092.00512974	0.46541166	−2091.533279	85	-
product **17**	−1338.86766731	0.28653899	−1338.577909	-	0
**20** + **17**	−2092.10331526	0.46843815	−2091.628438	−165	-

[a] Single-point energy computed at the ωB97M-V/def2-QZVP//PBE0-D3/def2-SVP+SMD(DCM) level of theory. The Gibbs energy of solvation and the dispersion correction are already included in the reported energies. [b] Thermostatistical contributions to the Gibbs energy computed at the PBE0-D3/def2-SVP+SMD(DCM) level of theory. The zero-point contributions, the volume work term to the enthalpy and all contributions to the inner energy and the entropy due to the thermal population of translational, rotational and vibrational states are included in the thermostatistical corrections. [c] Gibbs energy at a concentration of 1 mol/L in solution and a temperature of 313.15 K.

5.2.2 Kinetic analysis (Figure 12, Figure S16)

For the in-situ formation of dimethyl ketene (**21**) from isobutyryl chloride (**16**) and subsequent cyclization with *N*-alkynylated sulfoximine **9** to product **17** (see Figure S16), a kinetic analysis was performed to assess whether the DFT computed Gibbs energy barriers are of a reasonable magnitude for the reaction to occur under the experimental reaction conditions. From the mechanism in Figure S16, it is evident that intermediates **19** and **22** are short-lived species. Hence, it was assumed that the steady state assumption can be applied to the concentration changes of compounds **19** and **22**. Application of the latter approximation reduces the kinetic analysis to the treatment of the four reactions in Scheme S2 and Scheme S3, wherein the forward reactions and reverse reactions are characterized by the effective rate constants $k_{1,\mathrm{eff}}$, $k_{2,\mathrm{eff}}$, $k_{3,\mathrm{eff}}$ and $k_{4,\mathrm{eff}}$. The effective rate constants are defined according to Equations S13 to S16. The rate constants k_1 to k_4 and k_{-1} to k_{-4} used in Equations S13 to S16 can be quantified by employing the Eyring equation and the DFT computed barriers in Table S7 (in the Eyring equation a transmission coefficient of unity was assumed).[S36] The concentration changes for compounds **16**, **18**, **20**, **21**, **9** and **17** per unit time are thus given by Equations S17 to S19.

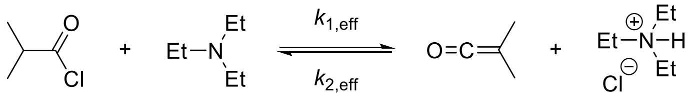

16 **18** **21** **20**

Scheme S2. Formation of dimethyl ketene (**21**) from isobutyryl chloride (**16**).

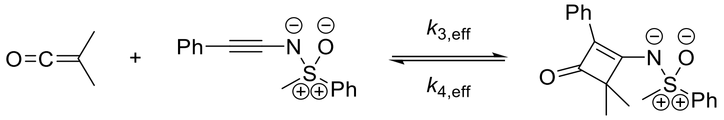

21 **9** **17**

Scheme S3. Formation of cyclic product **17** from *N*-alkynylated sulfoximine **9** and dimethyl ketene (**21**).

$$k_{1,\mathrm{eff}} = k_1 \frac{k_2}{k_{-1} + k_2}$$ **Equation S13**

$$k_{2,\mathrm{eff}} = k_{-2} \frac{k_{-1}}{k_{-1} + k_2}$$ **Equation S14**

$$k_{3,\mathrm{eff}} = k_3 \frac{k_4}{k_{-3} + k_4}$$ **Equation S15**

$$k_{4,\mathrm{eff}} = k_{-4} \frac{k_{-3}}{k_{-3} + k_4}$$ **Equation S16**

$k_{1,\mathrm{eff}}$ = Effective rate constant for the forward reaction in Scheme S2 ($L \cdot mol^{-1} \cdot s^{-1}$)
$k_{2,\mathrm{eff}}$ = Effective rate constant for the reverse reaction in Scheme S2 ($L \cdot mol^{-1} \cdot s^{-1}$)
$k_{3,\mathrm{eff}}$ = Effective rate constant for the forward reaction in Scheme S3 ($L \cdot mol^{-1} \cdot s^{-1}$)
$k_{4,\mathrm{eff}}$ = Effective rate constant for the reverse reaction in Scheme S3 ($L \cdot mol^{-1} \cdot s^{-1}$)
k_1 = Rate constant for the formation of intermediate **19** from isobutyryl chloride (**16**) and triethyl amine (**18**) ($L \cdot mol^{-1} \cdot s^{-1}$)
k_{-1} = Rate constant for the formation of isobutyryl chloride (**16**) and triethyl amine (**18**) from intermediate **19** (s^{-1})
k_2 = Rate constant for the formation of ketene **21** and triethylammonium chloride (**20**) from intermediate **19** (s^{-1})
k_{-2} = Rate constant for the formation of intermediate **19** from ketene **21** and triethylammonium chloride (**20**) ($L \cdot mol^{-1} \cdot s^{-1}$)
k_3 = Rate constant for the formation of intermediate **22** from *N*-alkynylated sulfoximine **9** and ketene **21** ($L \cdot mol^{-1} \cdot s^{-1}$)
k_{-3} = Rate constant for the formation of *N*-alkynylated sulfoximine **9** and ketene **21** from intermediate **22** (s^{-1})
k_4 = Rate constant for the formation of product **17** from intermediate **22** (s^{-1})
k_{-4} = Rate constant for the formation of intermediate **22** from product **17** (s^{-1})

$$\frac{d[\mathbf{16}]}{dt} = \frac{d[\mathbf{18}]}{dt} = -\frac{d[\mathbf{20}]}{dt} = -k_{1,\text{eff}}[\mathbf{16}][\mathbf{18}] + k_{2,\text{eff}}[\mathbf{20}][\mathbf{21}]$$ **Equation S17**

$$\frac{d[\mathbf{21}]}{dt} = k_{1,\text{eff}}[\mathbf{16}][\mathbf{18}] - k_{2,\text{eff}}[\mathbf{20}][\mathbf{21}] - k_{3,\text{eff}}[\mathbf{9}][\mathbf{21}] + k_{4,\text{eff}}[\mathbf{17}]$$ **Equation S18**

$$\frac{d[\mathbf{9}]}{dt} = -\frac{d[\mathbf{17}]}{dt} = -k_{3,\text{eff}}[\mathbf{9}][\mathbf{21}] + k_{4,\text{eff}}[\mathbf{17}]$$ **Equation S19**

$[\mathbf{16}]$ = Concentration of compound **16** (mol·L^{-1})
$[\mathbf{18}]$ = Concentration of compound **18** (mol·L^{-1})
$[\mathbf{20}]$ = Concentration of compound **20** (mol·L^{-1})
$[\mathbf{21}]$ = Concentration of compound **21** (mol·L^{-1})
$[\mathbf{17}]$ = Concentration of compound **17** (mol·L^{-1})
$[\mathbf{9}]$ = Concentration of compound **9** (mol·L^{-1})

With the computed effective rate constants, the differential equations in Equations S17 to S19 were solved numerically, whereby the required initial concentrations for compounds **16**, **18**, **20**, **21**, **9** and **17** were taken from the literature protocol ([**16**] = 0.230 mol·L^{-1}, [**18**] = 0.253 mol·L^{-1}, [**9**] = 0.115 mol·L^{-1}, [**20**] = [**21**] = [**17**] = 0 mol·L^{-1}).[S38] The numerically obtained evolution of the yield over time is shown in Figure S17. For the experimental reaction time of 16 h, a yield of 45% was calculated numerically, which is lower than the experimentally observed value of 98%. The latter finding indicates that the computed Gibbs free energy barriers are slightly too high. To demonstrate that the computed barriers, nonetheless, are of the correct magnitude, the experimental yield was reproduced by reducing the computed barriers and solving the differential equations in Equations S17 to S19 with the modified values. Reducing the Gibbs energy barriers for the reactions of compounds **16** and **18** to intermediate **19**, and *N*-alkynylated sulfoximine **9** and dimethyl ketene **21** to intermediate **22** each by 1 kcal/mol, allowed numerically computing a yield of 99% (see Figure S18). It is worth noting that DFT-computed barriers can be considered quite accurate within 2 kcal/mol of the experimental value.[S28,S39] Since only relatively small adjustments to the Gibbs energy profile sufficed to reproduce the experimental yield, the Gibbs energy barriers in Figure S16 appear to be of the correct magnitude.

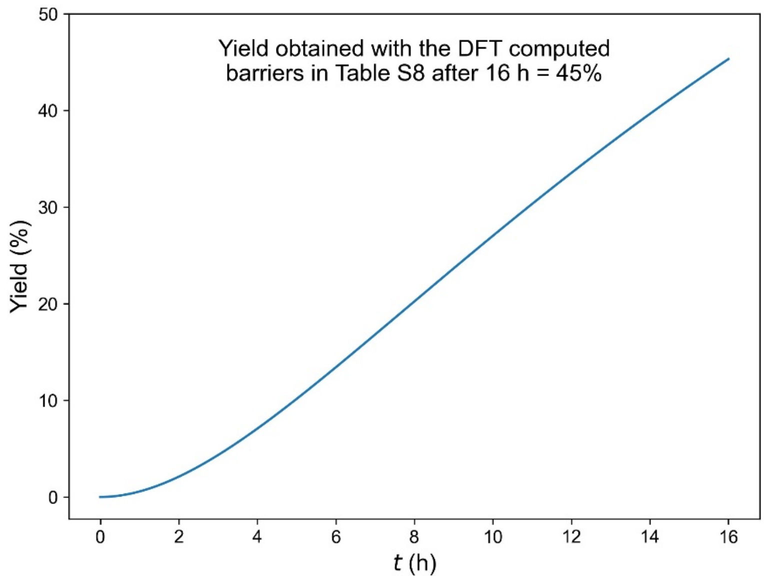

Figure S17. Computed yield of cyclic product **17** which was obtained by numerically solving the differential equations in Equations S17 to S19 and employing the DFT computed barriers in Table S7/Table S8.

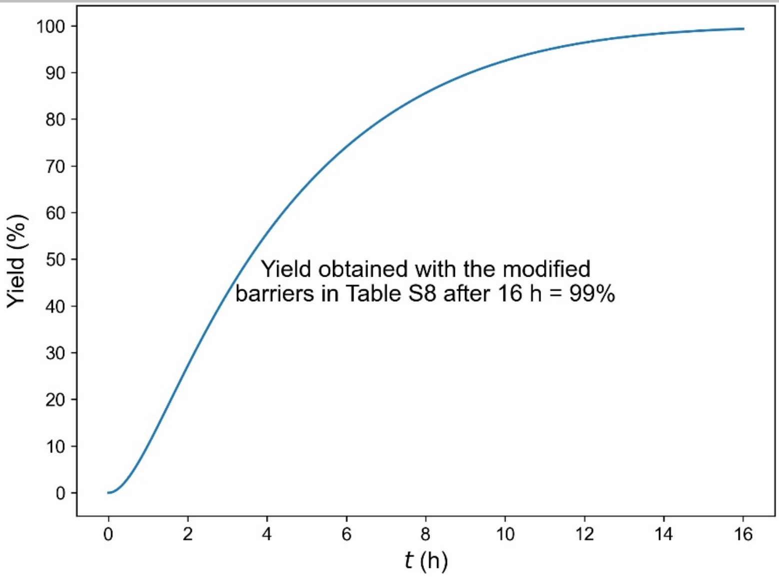

Figure S18. Computed yield of cyclic product **17** which was obtained by numerically solving the differential equations in Equations S17 to S19 and employing the modified barriers in Table S8.

Table S8: Gibbs energy barriers employed to compute the yield evolutions in Figures S17 and S18. The barriers ΔG_1 to ΔG_{-4} were employed to calculate the rate constants k_1 to k_{-4}, respectively, via the Eyring equation. The rate constants k_1 to k_{-4} are utilized in Equations S13 to S16 to compute the effective rate constants $k_{1,\text{eff}}$ to $k_{4,\text{eff}}$, which are in turn employed in Equations S17 to S19.

Gibbs free energy barriers associated with rate constants k_1 to k_{-4}	DFT computed barriers (kJ/mol)	modified barriers (kJ/mol)
ΔG_1	103.2	99.0
ΔG_{-1}	31.3	31.3
ΔG_2	19.0	19.0
ΔG_{-2}	97.3	97.3
ΔG_3	96.8	92.6
ΔG_{-3}	25.4	25.4
ΔG_4	19.8	19.8
ΔG_{-4}	249.8	249.8

5.2.3 Relaxed surface scan—Concerted formation of cyclic product 17 (alternative mechanism 1)

To provide additional proof for the proposed sequential mechanism in the cyclization of *N*-alkynylated sulfoximine **9** with dimethyl ketene (**21**) under formation of compound **17**, a relaxed surface scan corresponding to a concerted process was performed at the PBE0-D3/def2-SVP+SMD(DCM) level of theory (SCF convergence threshold = VeryTightSCF, numerical grid = DEFGRID3). Thereby, the distances *d*(C10–C34) and *d*(C11–C33) in Figure S19 were constrained in each step of the scan to identical values. The energies of the relaxed surface scan are summarized in Figure S20 and are compared to the energy of the first, rate limiting transition state in the sequential addition of dimethyl ketene (**21**) to *N*-alkynylated sulfoximine **9**. As can be observed from Figure S20, the energy of the strictly concerted process is significantly higher than the energy of transition state **TS6** of the sequential mechanism between approximately 2.0 and 2.6 Å. This indicates that a sequential mechanism is more likely than the concerted process.

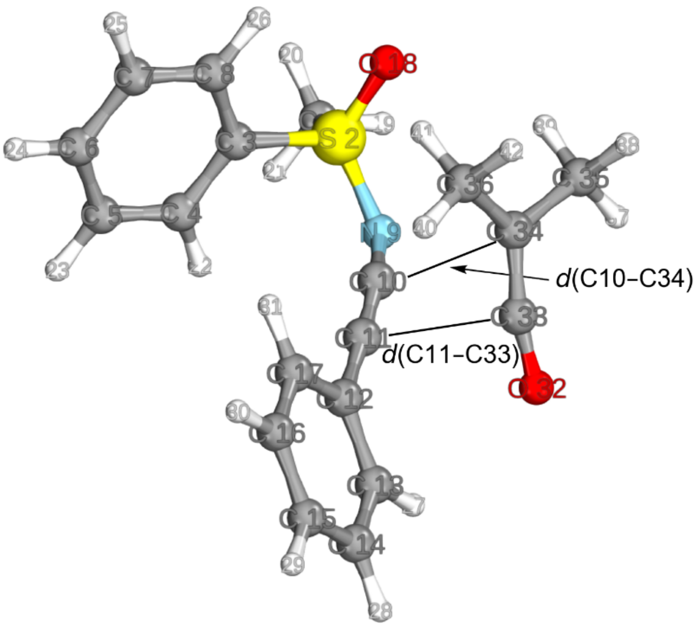

Figure S19. Scan coordinates *d*(C10–C34) and *d*(C11–C33) in the relaxed surface scan for the strictly concerted addition of dimethyl ketene (**21**) to *N*-alkynylated sulfoximine **9**.

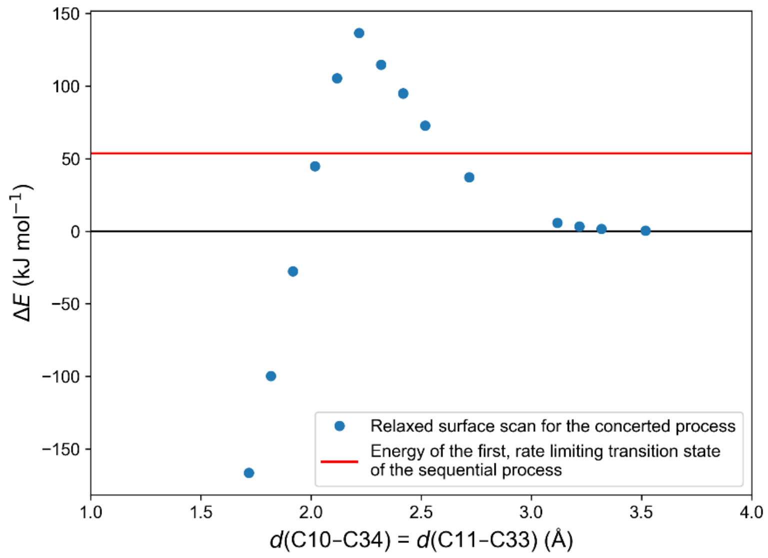

Figure S20. Relative energies from the relaxed surface scan for the concerted addition of dimethyl ketene (**21**) to *N*-alkynylated sulfoximine **9** (blue dots), which are compared to the relative energy of the first, rate limiting transition state (**TS6**) of the sequential process (red line). All energies were obtained at the PBE0-D3/def2-SVP+SMD(DCM) level of theory (SCF convergence threshold = VeryTightSCF, numerical grid = DEFGRID3).

5.2.4 Cyclization of *N*-alkynylated sulfoximine 9 with isobutyryl chloride (16)—Results for alternative mechanism 2

For the formation of cyclic product **17**, the mechanistic pathway in Figure S21 can also be envisioned. Therein, *N*-alkynylated sulfoximine **9** reacts directly with isobutyryl chloride (**16**) to give intermediate **29**. The latter is then deprotonated and subsequently cyclizes to product **17**. The Gibbs energy profile for this alternative mechanism was evaluated at the ωB97M-V/def2-QZVP//PBE0-D3/def2-SVP+SMD(DCM) level of theory and is included in Figure S21. The single point energies, thermostatistical corrections and Gibbs energies of all structures are summarized in Table S9. As can be observed from the Gibbs energy profile in Figure S21, significantly higher barriers were computed for this alternative mechanism compared to the mechanism in Figure S16, making this alternative pathway less likely.

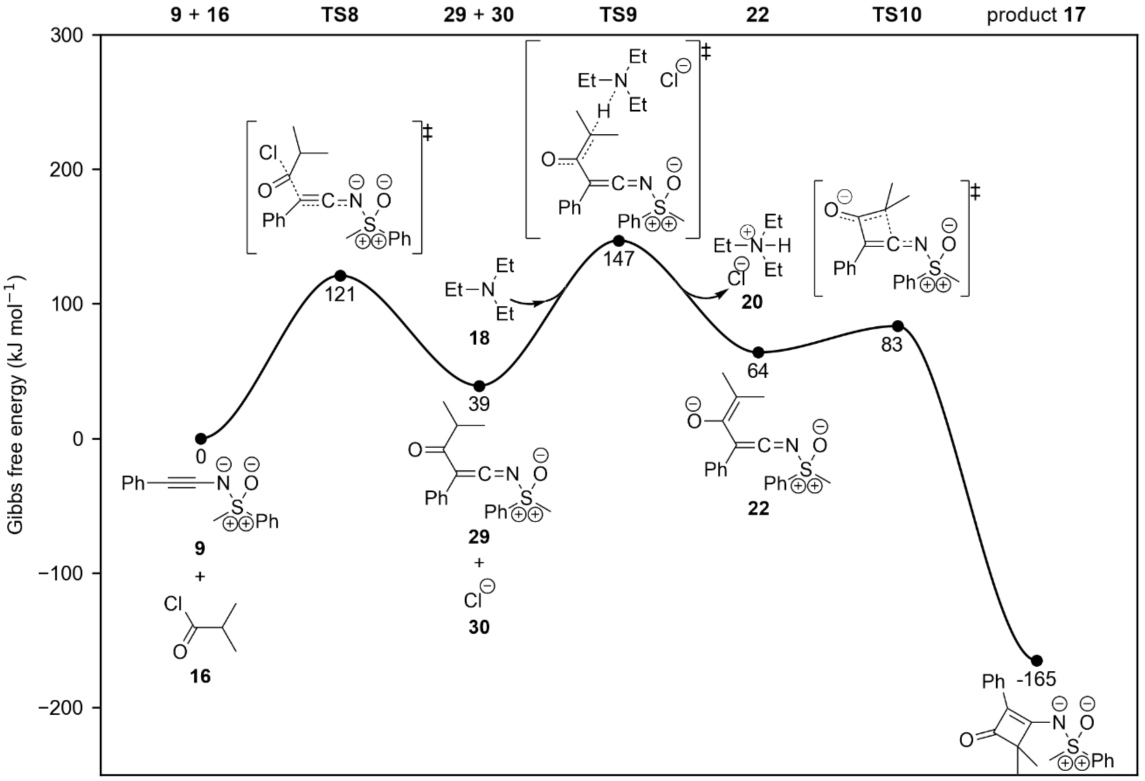

Figure S21. Gibbs energy barriers for alternative mechanism 2 leading to cyclization product **17** assessed at the ωB97M-V/def2-QZVP//PBE0-D3/def2-SVP level of theory in an SMD DCM solvent environment. Gibbs energies were computed at a temperature of 313.15 K and for a concentration of 1 mol/L in solution.

Table S9: Gibbs energies of all stationary points in the alternative mechanism 2 in Figure S21.

Structure	$E^{\omega B97M\text{-}V}$ (hartree)[a]	$G^{PBE0} - E^{PBE0}$ (hartree)[b]	$G = E^{\omega B97M\text{-}V} + (G^{PBE0} - E^{PBE0}) + k_B T \ln(V_m)$ (hartree)[c]	ΔG (kJ/mol)	imaginary frequencies
sulfoximine **9**	−1107.54065719	0.19617308	−1107.341265	-	0
**16**	−692.09418963	0.07104717	−692.019923	-	0
amine **18**	−292.37738381	0.16977769	−292.204387	-	0
**18** + **9** + **16**	−2092.01223064	0.43699794	−2091.565575	0	-
**TS8**	−1799.61116454	0.29284071	−1799.315104	-	1
**18** + **TS8**	−2091.98854835	0.46261840	−2091.519491	121	-
**29**	−1339.26511290	0.29458266	−1338.967311	-	0
chloride **30**	−460.36628394	−0.01592061	−460.378985	-	-

***Table S9* (continued):** Gibbs energies of all stationary points in the alternative mechanism 2 in Figure S21.

Structure	$E^{\omega B97M\text{-}V}$ (hartree)[a]	$G^{PBE0} - E^{PBE0}$ (hartree)[b]	$G = E^{\omega B97M\text{-}V} + (G^{PBE0} - E^{PBE0}) + k_B T\ln(V_m)$ (hartree)[c]	ΔG (kJ/mol)	imaginary frequencies
**18** + **29** + **30**	−2092.00878065	0.44843974	−2091.550683	39	-
**TS9**	−2091.99825346	0.48546905	−2091.509565	-	1
**20**	−753.23564795	0.18189916	−753.050529	-	0
**22**[d]	−1338.77566574	0.28172369	−1338.490723	-	0
**20** + **22**	−2092.01131369	0.46362285	−2091.541252	64	-
**TS10**	−1338.76812081	0.28163785	−1338.483264	-	1
**20** + **TS10**	−2092.00376875	0.46353701	−2091.533793	83	-
product **17**	−1338.86766731	0.28653899	−1338.577909	-	0
**20** + **17**	−2092.10331526	0.46843815	−2091.628438	−165	-

[a] Single-point energy computed at the ωB97M-V/def2-QZVP//PBE0-D3/def2-SVP+SMD(DCM) level of theory. The Gibbs energy of solvation and the dispersion correction are already included in the reported energies. [b] Thermostatistical contributions to the Gibbs energy computed at the PBE0-D3/def2-SVP+SMD(DCM) level of theory. The zero-point contributions, the volume work term to the enthalpy and all contributions to the inner energy and the entropy due to the thermal population of translational, rotational and vibrational states are included in the thermostatistical corrections. [c] Gibbs energy at a concentration of 1 mol/L in solution and a temperature of 313.15 K. [d] The conformer search for structure **22** was conducted as described in section 1.2 with one modification: All conformers within 8 kcal/mol of the lowest conformer were retained after the CREST run and were subsequently subjected to energetic sorting at higher DFT levels.

5.2.5 Cyclization of *N*-alkynylated sulfoximine 9 with isobutyryl chloride (16)—Results for alternative mechanism 3

Besides the alternative mechanism discussed in section 5.2.4, the alternative pathways shown in Scheme S4 constitute additional possibilities. Therein, the formation of the common intermediate **31** is assumed which in a subsequent addition reaction gives compound **32**. Intermediate **32** is then deprotonated and subsequently cyclizes to the experimentally observed product **17**.

In the first pathway, formation of the common intermediate **31** occurs through the deprotonation of isobutyryl chloride by *N*-alkynylated sulfoximine **9**. However, when assessing the deprotonation of isobutyryl chloride (**16**) by *N*-alkynylated sulfoximine **9** via a relaxed surface scan at the PBE0-D3/def2-SVP+SMD(DCM) level of theory (SCF convergence threshold = VeryTightSCF, numerical grid = DEFGRID3), no minimum on the PES could be identified for structure **31**. The results from the relaxed surface scan are summarized in Figure S22. The utilized scan coordinate is defined in Figure S23. Besides the absence of an energy minimum, a rather significant energy increase of 138.7 kJ/mol was observed. In comparison, transition state **TS4** in the mechanism in Figure S16 was 25.9 kJ/mol higher in energy than the reactants at the same level of theory. Thus, the 138.7 kJ/mol energy increase computed through the relaxed surface scan appears to be significantly too high. Based on the absent PES minimum and the unfavorable nature of the process, the deprotonation of isobutyryl chloride (**16**) by *N*-alkynylated sulfoximine **9** was considered unlikely.

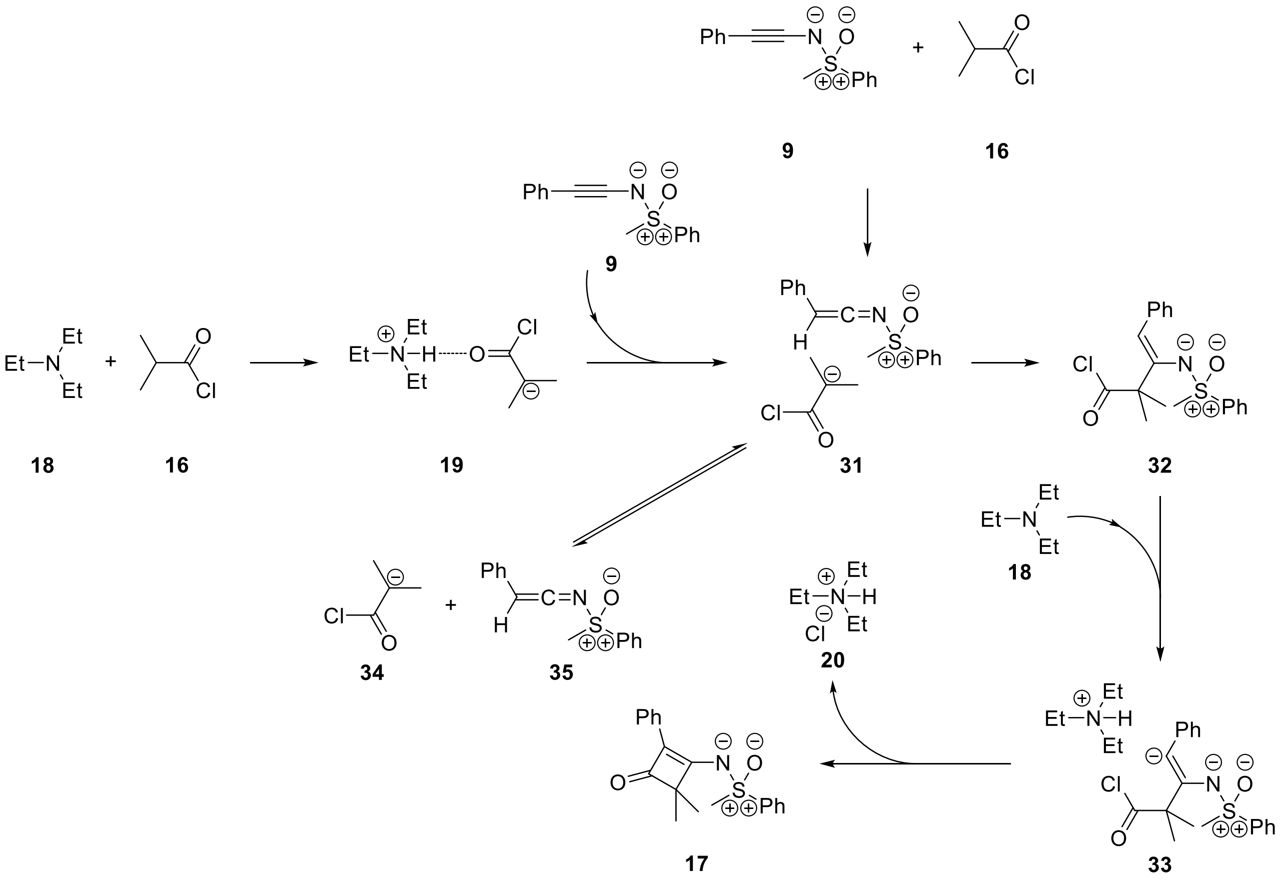

Scheme S4. Another mechanism for the formation of cyclic product **17**.

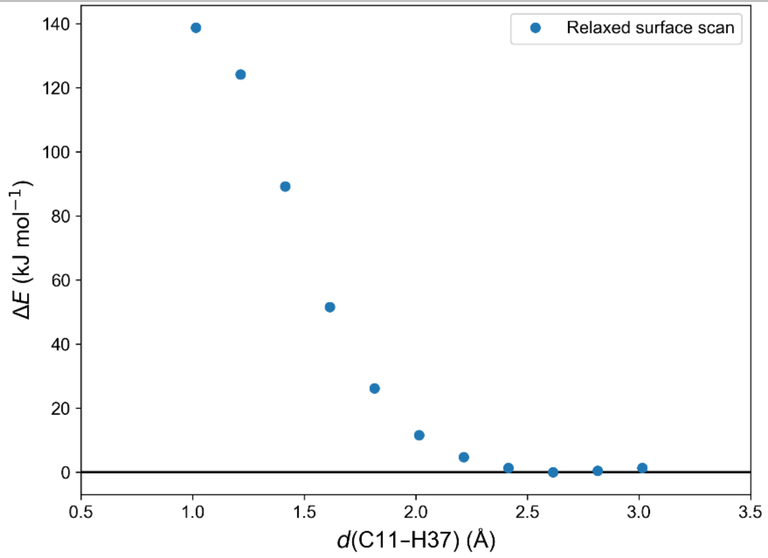

Figure S22. Relative energies from the relaxed surface scan for the deprotonation of isobutyryl chloride (**16**) by *N*-alkynylated sulfoximine **9** plotted versus the scan coordinate. The scan coordinate corresponds to the distance between the alkynic carbon in *β*-position to the sulfoximidoyl nitrogen in *N*-alkynylated sulfoximine **9** and the hydrogen of the CH-group in isobutyryl chloride (**16**) (see Figure S23).

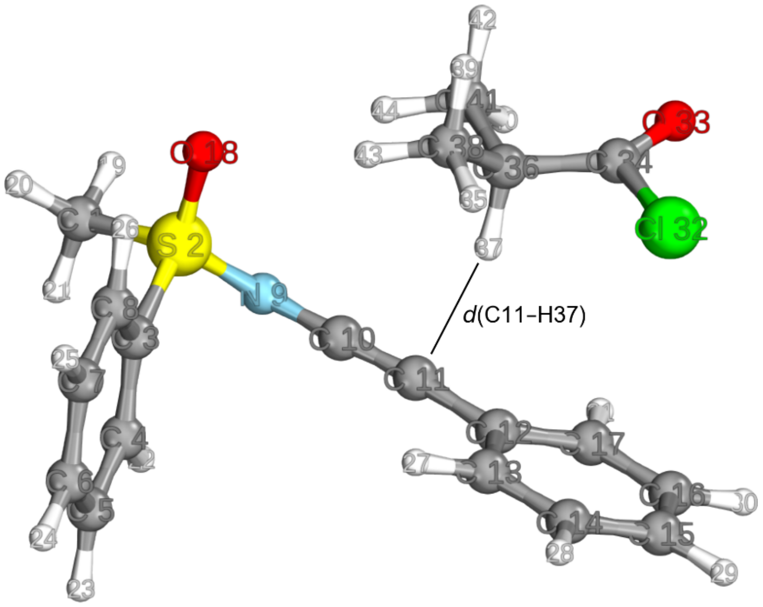

Figure S23. Scan coordinate *d*(C11–H37) for the relaxed surface scan, which is discussed in the current section.

In the second pathway, the formation of the key ion pair intermediate **31** occurs through the protonation of *N*-alkynylated sulfoximine **9** by the triethylammonium cation, which can be formed under the experimental conditions from isobutyryl chloride (**16**) and triethylamine (**18**). To assess the likelihood of this alternative process, the Gibbs energy of the ion pair intermediate **31** and the Gibbs energies of the two ions **34** and **35** were evaluated at the ωB97M-V/def2-QZVP//PBE0-D3/def2-SVP+SMD(DCM) level of theory (SCF convergence = VeryTightSCF, numerical grid = DEFGRID3). The computed values are compared in Figure S24 with the Gibbs energy profile of the minimum Gibbs energy pathway previously shown in Figure S16. As can be observed from Figure S24, the relative Gibbs energy associated with intermediate **31** and the relative Gibbs energy associated with the dissociated ions **34** and **35** both lie significantly above the Gibbs energy profile of the preferred pathway. Based on the insufficient stability of intermediate **31**, the second pathway via protonation of *N*-alkynylated sulfoximine **9** by the ion pair **19** can be ruled out.

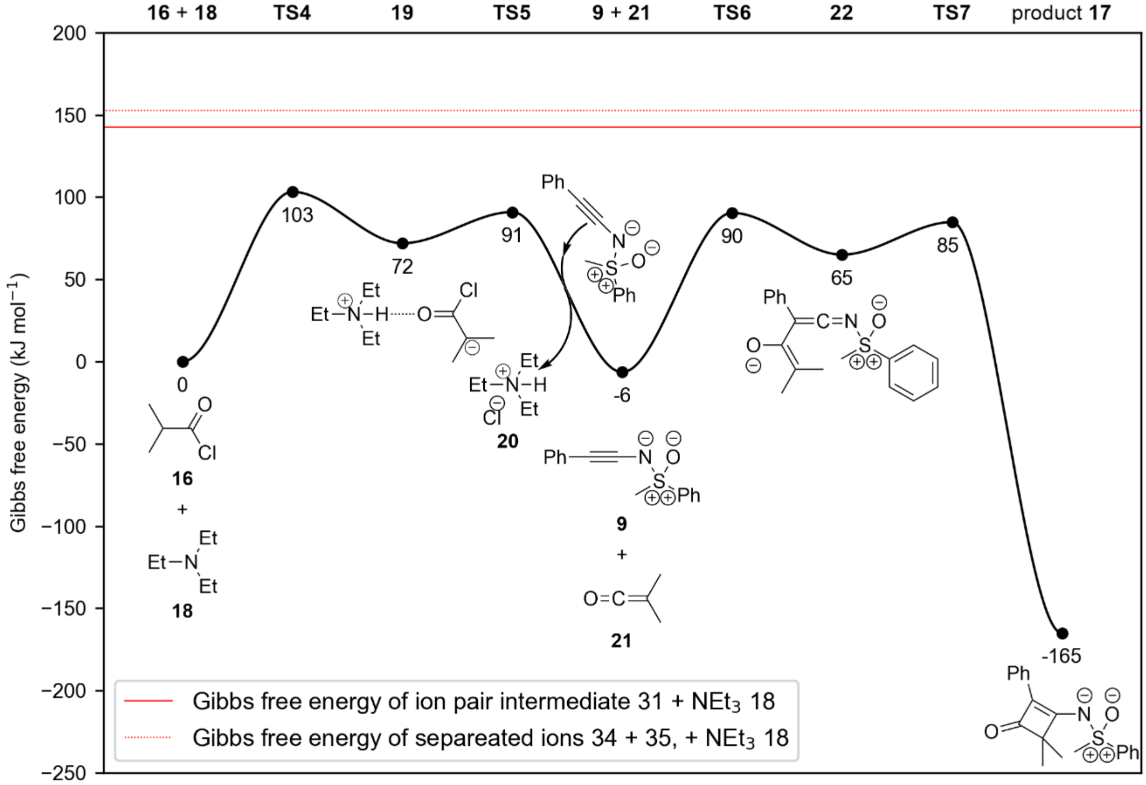

Figure S24. The Gibbs energy profile of the minimum Gibbs energy pathway (black), which is compared to the relative Gibbs energy of intermediate **31** which appears in the alternative mechanisms shown in Scheme S4 (red horizontal line).

6 References

[S1] C. Bannwarth, S. Ehlert, S. Grimme, *J. Chem. Theory Comput.* **2019**, *15*, 1652–1671.

[S2] a) E. Caldeweyher, C. Bannwarth, S. Grimme, *J. Chem. Phys.* **2017**, *147*, 034112; b) E. Caldeweyher, S. Ehlert, A. Hansen, H. Neugebauer, S. Spicher, C. Bannwarth, S. Grimme, *J. Chem. Phys.* **2019**, *150*, 154122; c) E. Caldeweyher, J.-M. Mewes, S. Ehlert and S. Grimme, *Phys. Chem. Chem. Phys.* **2020**, *22*, 8499.

[S3] S. Ehlert, M. Stahn, S. Spicher, S. Grimme, *J. Chem. Theory Comput.* **2021**, *17*, 4250–4261.

[S4] C. Bannwarth, E. Caldeweyher, S. Ehlert, A. Hansen, P. Pracht, J. Seibert, S. Spicher, S. Grimme, *WIREs Comput. Mol. Sci.* **2021**, *11*, e1493.

[S5] a) S. Grimme, *J. Chem. Theory Comput.* **2019**, *15*, 2847–2862; b) P. Pracht, F. Bohle, S. Grimme, *Phys. Chem. Chem. Phys.* **2020**, *22*, 7169–7192.

[S6] a) F. Neese, *WIREs Comput. Mol. Sci.* **2012**, *2*, 73–78; b) F. Neese, *WIREs Comput. Mol. Sci.* **2018**, *8*, e1327; c) F. Neese, F. Wennmohs, U. Becker, C. Riplinger, *J. Chem. Phys.* **2020**, *152*, 224108; d) F. Neese, *WIREs Comput. Mol. Sci.* **2022**, *12*, e1606.

[S7] F. Neese, *J. Comput. Chem.* **2003**, *24*, 1740–1747.

[S8] F. Neese, F. Wennmohs, A. Hansen, U. Becker, *Chem. Phys.* **2009**, *356*, 98–109.

[S9] T. A. Young, J. J. Silcock, A. J. Sterling, F. Duarte, *Angew. Chem. Int. Ed.* **2021**, *60*, 4266–4274.

[S10] S. Grimme, F. Bohle, A. Hansen, P. Pracht, S. Spicher, M. Stahn, *J. Phys. Chem. A* **2021**, *125*, 4039–4054.

[S11] A. V. Marenich, C. J. Cramer, D. G. Truhlar, *J. Phys. Chem. B* **2009**, *113*, 6378–6396.

[S12] S. Spicher, S. Grimme, *J. Chem. Theory Comput.* **2021**, *17*, 1701–1714.

[S13] S. Grimme, A. Hansen, S. Ehlert, J.-M. Mewes, *J. Chem. Phys.* **2021**, *154*, 064103.

[S14] H. Kruse, S. Grimme, *J. Chem. Phys.* **2012**, *136*, 154101.

[S15] a) J. P. Perdew, M. Ernzerhof, K. Burke, *J. Chem. Phys.* **1996**, *105*, 9982–9985; b) C. Adamo, V. Barone, *J. Chem. Phys.* **1999**, *110*, 6158–6170.

[S16] a) S. Grimme, J. Antony, S. Ehrlich, H. Krieg, *J. Chem. Phys.* **2010**, *132*, 154104; b) S. Grimme, S. Ehrlich, L. Goerigk, *J. Comput. Chem.* **2011**, *32*, 1456–1465.

[S17] F. Weigend, R. Ahlrichs, *Phys. Chem. Chem. Phys.* **2005**, *7*, 3297–3305.

[S18] F. Weigend, *Phys. Chem. Chem. Phys.* **2006**, *8*, 1057–1065.

[S19] a) D. C. Young, *Computational Chemistry: A Practical Guide for Applying Techniques to Real-World Problems*, John Wiley & Sons, Inc., New York, U.S.A., **2001**; b) F. Jensen, *Introduction to Computational Chemistry*, 2nd edition, John Wiley & Sons, Ltd, Chichester, England, **2007**.

[S20] G. Knizia, IboView [v20150427], A program for chemical analysis, Pennsylvania State University, Pennsylvania (U.S.A.), **2015**.

[S21] A. D. Becke, *J. Chem. Phys.* **1993**, *98*, 5648–5652.

[S22] a) A. D. McLean, G. S. Chandler, *J. Chem. Phys.* **1980**, *72*, 5639–5648; b) R. Krishnan, J. S. Binkley, R. Seeger, J. A. Pople, *J. Chem. Phys.* **1980**, *72*, 650–654; c) J.-P. Blaudeau, M. P. McGrath, L. A. Curtiss, L. Radom, *J. Chem. Phys.* **1997**, *107*, 5016–5021; d) A. J. H. Wachters, *J. Chem. Phys.* **1970**, *52*, 1033–1036; e) P. J. Hay, *J. Chem. Phys.* **1977**, *66*, 4377–4384; f) K. Raghavachari, G. W. Trucks, *J. Chem. Phys.* **1989**, *91*, 1062–1065; g) R. C. Binning, L. A. Curtiss, *J. Comput. Chem.* **1990**, *11*, 1206–1216; h) M. P. McGrath, L. Radom, *J. Chem. Phys.* **1991**, *94*, 511–516; i) L. A. Curtiss, M. P. McGrath, J.-P. Blaudeau, N. E. Davis, R. C. Binning, L. Radom, *J. Chem. Phys.* **1995**, *103*, 6104–6113.

[S23] T. Clark, J. Chandrasekhar, G. W. Spitznagel, P. von Ragué Schleyer, *J. Comput. Chem.* **1983**, *4*, 294–301.

[S24] Gaussian 16, Revision C.01, M. J. Frisch, G. W. Trucks, H. B. Schlegel, G. E. Scuseria, M. A. Robb, J. R. Cheeseman, G. Scalmani, V. Barone, G. A. Petersson, H. Nakatsuji, X. Li, M. Caricato, A. V. Marenich, J. Bloino. B. G. Janesko, R. Gomperts, B. Mennucci, H. P. Hratchian, J. V. Ortiz, A. F. Izmaylov, J. L. Sonnenberg, D. Williams-Young, F. Ding, F. Lipparini, F. Egidi, J. Goings, B. Peng, A. Petrone, T. Henderson, D. Ranasinghe, V. G. Zakrzewski, J. Gao, N. Rega, G. Zheng, W. Liang, M. Hada, M. Ehara, K. Toyota, R. Fukuda, J. Hasegawa, M. Ishida, T. Nakajima, Y. Honda, O. Kitao, H. Nakai, T. Vreven, K. Throssell, J. A. Montgomery, Jr., J. E. Peralta, F. Ogliaro, M. J. Bearpark, J. J. Heyd, E. N. Brothers, K. N. Kudin, V. N. Staroverov, T. A. Keith, R. Kobayashi, J. Normand, K. Raghavachari, A. P. Rendell, J. C. Burant, S. S. Iyengar, J. Tomasi, M. Cossi, J. M. Millam, M. Klene, C. Adamo, R. Cammi, J. W. Ochterski, R. L. Martin, K. Morokuma, O. Farkas, J. B. Foresman, and D. J. Fox, Gaussian, Inc., Wallingford CT, 2019.

[S25] a) J. P. Foster, F. Weinhold, *J. Am. Chem. Soc.* **1980**, *102*, 7211–7218; b) A. E. Reed, F. Weinhold, *J. Chem. Phys.* **1983**, *78*, 4066–4073; c) A. E. Reed, R. B. Weinstock, F. Weinhold, *J. Chem. Phys.* **1985**, *83*, 735–746; d) A. E. Reed, F. Weinhold, *J. Chem. Phys.* **1985**, *83*, 1736–1740; e) J. E. Carpenter, PhD thesis, University of Wisconsin (U.S.A), **1987**; f) J. E. Carpenter, F. Weinhold, *J. Mol. Struct.: THEOCHEM* **1988**, *169*, 41–62; g) A. E. Reed, L. A. Curtiss, F. Weinhold, *Chem. Rev.* **1988**, *88*, 899–926; h) F. Weinhold, J. E. Carpenter in *The Structure of Small Molecules and Ions* (Eds.: R. Naaman, Z. Vager), Plenum Press, New York, **1988**, pp. 227–236.

[S26] E. D. Glendening, A. E. Reed, J. E. Carpenter, F. Weinhold, NBO Version 3.1.

[S27] C. Hansch, A. Leo, R. W. Taft, *Chem. Rev.* **1991**, *91*, 165–195.

[S28] N. Mardirossian, M. Head-Gordon, *J. Chem. Phys.* **2016**, *144*, 214110.

[S29] S. Lehtola, C. Steigemann, M. J. T. Oliveira, M. A. L. Marques, *SoftwareX* **2018**, *7*, 1–5.

[S30] a) O. A. Vydrov, T. Van Voorhis, *J. Chem. Phys.* **2010**, *133*, 244103; b) W. Hujo, S. Grimme, *J. Chem. Theory Comput.* **2011**, *7*, 3866–3871.

[S31] V. Ásgeirsson, B. O. Birgisson, R. Bjornsson, U. Becker, F. Neese, C. Riplinger, H. Jónsson, *J. Chem. Theory Comput.* **2021**, *17*, 4929–4945.

[S32] K. Ishida, K. Morokuma, A. Komornicki, *J. Chem. Phys.* **1977**, *66*, 2153–2156.

[S33] GaussView 6.1.1, R. Dennington, T. A. Keith, J. M. Millam, Semichem Inc., Shawnee Mission, KS, 2016.

[S34] G. Knizia, J. E. M. N. Klein, *Angew. Chem. Int. Ed.* **2015**, *54*, 5518–5522.

[S35] R. G. Mortimer, *Physical Chemistry*, 2nd edition, Academic Press, San Diego, **2000**, pp. 412–414.

[S36] H. Eyring, *Chem. Rev.* **1935**, *17*, 65–77.

[S37] R. Pirwerdjan, P. Becker, C. Bolm, *Org. Lett.* **2015**, *17*, 5008–5011.

[S38] R. Pirwerdjan, D. L. Priebbenow, P. Becker, P. Lamers, C. Bolm, *Org. Lett.* **2013**, *15*, 5397–5399.

[S39] M. Bursch, J.-M. Mewes, A. Hansen, S. Grimme, *Angew. Chem. Int. Ed.* **2022**, *61*, e202205735.

7 Cartesian coordinates

7.1 Addition of benzoic acid (10) to *N*-alkynylated sulfoximine 9—Cartesian coordinates

Cartesian coordinates of the PBE0-D3/def2-SVP+SMD(THF) optimized geometry of sulfoximine **9**
$E^{\omega B97M\text{-}V}$ = −1107.53691132 hartree
Neutral, Singlet

C	3.13875334099020	-1.96326690854545	-2.04235546061150
S	1.86598387336996	-1.60843545042868	-0.87553758781953
C	2.30207468694710	-0.05688076597708	-0.11180028680966
C	2.16378734476202	1.12683695970036	-0.83775276543727
C	2.49776216786239	2.32744174368700	-0.21777612060908
C	2.95102787114394	2.33158286770852	1.10319917335014
C	3.07349251589307	1.13813182336398	1.81472532962297
C	2.74729483694119	-0.07334760849130	1.20779002110136
N	0.61038537132636	-1.39161390741713	-1.81057863577602
C	-0.51291668334910	-0.98567200112742	-1.22713062557037
C	-1.57941962376985	-0.60965919915979	-0.75943394395395
C	-2.80989562456073	-0.17054879656751	-0.19068086383120
C	-2.89971423004455	0.12889595603383	1.18429686094894
C	-4.10204072278535	0.55615060568948	1.73915784405745
C	-5.23998786968040	0.69576912038784	0.94249984508857
C	-5.16337724447626	0.40335328483904	-0.42030969267364
C	-3.96562643524738	-0.02502392304440	-0.98472736349173
O	1.83270804002974	-2.63548321763327	0.17384407470603
H	2.83800183181482	-2.89433880011214	-2.54346177253931
H	4.06921892790026	-2.10348302698346	-1.47709373248556
H	3.22196463647413	-1.13870371970896	-2.76135791481628
H	1.79257344855006	1.11012869883398	-1.86515261614490
H	2.39596193917100	3.26619554661983	-0.76693612497081
H	3.20687503615997	3.27864456519120	1.58461017684068
H	3.42228822831845	1.14795643439715	2.84991847955389
H	2.82664769374857	-1.02117477074325	1.74357254432641
H	-2.01171349573252	0.02133001997346	1.81152233405928
H	-4.15097633755487	0.78268102303433	2.80759219165943
H	-6.18260062804225	1.03110697964446	1.38212967813028
H	-6.04842880223845	0.50961253240221	-1.05325392817031
H	-3.91156525262153	-0.25323758326682	-2.05167124023434

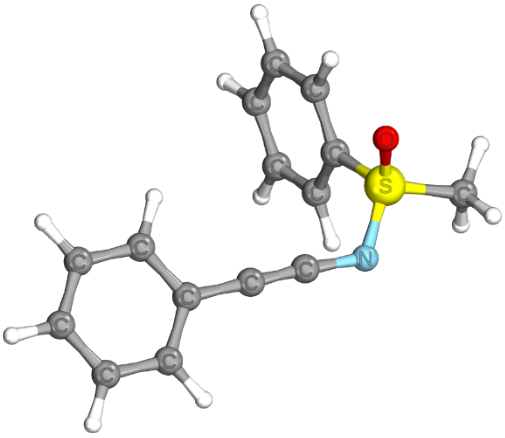

Cartesian coordinates of the PBE0-D3/def2-SVP+SMD(THF) optimized geometry of benzoic acid (**10**)
$E^{\omega B97M\text{-}V}$ = −420.88366595 hartree
Neutral, Singlet

O	2.30113895506710	-1.11721800340446	-0.00162081495035
C	1.67032610919311	-0.08682891997155	-0.00073715246737
O	2.25638025595175	1.11299568320492	-0.00016432648526
C	0.18580844252736	-0.01246438250393	-0.00036112706898
C	-0.49658241000207	1.21047434180246	0.00093517617081
C	-1.88907093140087	1.22714769674575	0.00127769462641
C	-2.60306298417487	0.02804675681960	0.00030722730150
C	-1.92489548337737	-1.19248355149274	-0.00099693081372
C	-0.53389667769881	-1.21336463937254	-0.00132163445029
H	3.21650054764514	0.96261433055043	-0.00047258856348
H	0.06599739559998	2.14547619402597	0.00168305348415
H	-2.42118567227886	2.18149248846114	0.00231060801985
H	-3.69599966822984	0.04462898049173	0.00056931760501
H	-2.48482224441062	-2.13077988088014	-0.00176505756436
H	0.01576740438889	-2.15698777197663	-0.00232330734393

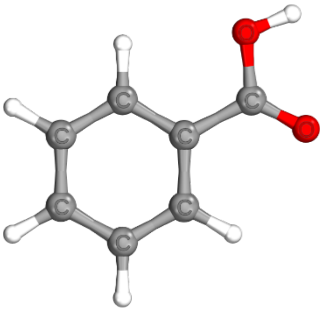

Cartesian coordinates of the PBE0-D3/def2-SVP+SMD(THF) optimized geometry of **TS1**

$E^{\omega B97M\text{-}V}$ = −1528.40411068 hartree

Neutral, Singlet

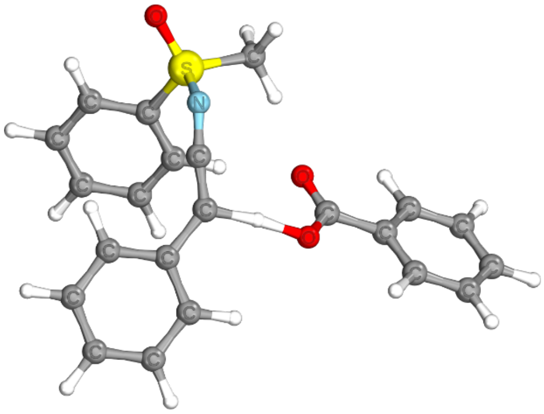

C	2.54187905982322	-1.60356674786009	1.71408505144600
S	2.58608854859591	0.12579437293524	2.01452042860753
C	2.73072782672281	0.96756491568490	0.47389234260017
C	1.98631679943188	0.55872129980126	-0.63612183946172
C	2.05193942530290	1.34692867178940	-1.78323742495145
C	2.83005996882384	2.50449525609036	-1.80329778626021
C	3.56195837286959	2.89079514020796	-0.67866330885101
C	3.51374917781710	2.12369028340706	0.48051673866652
N	1.10177193599563	0.47242379885481	2.68444962517655
C	0.16792398205660	0.83096322555485	1.91173659543802
C	-0.80444385473857	1.03394810752624	1.10525715943871
C	-1.33833913092738	2.21304375620539	0.43669109790652
C	-2.46966629472364	2.08592008940369	-0.38560734449384
C	-2.97980069889101	3.19194936071114	-1.06210501673689
C	-2.36671076542802	4.43812043161867	-0.93378165530989
C	-1.23563108463069	4.57378513806343	-0.12430532641822
C	-0.72411041001200	3.47373279902424	0.55509800759748
O	3.64688536345100	0.50296473821215	2.93969381206792
H	2.40562095268261	-2.06124945267736	2.70503354931179
H	3.51068651876118	-1.88598562731098	1.27786292008837
H	1.68905241566309	-1.83425480666381	1.04993868336414
H	1.35836112466702	-0.33882050427389	-0.60953333474901
H	1.48040728036705	1.05287825299194	-2.66630192918057
H	2.86800011905899	3.11507234284936	-2.70862954327139
H	4.17366954182043	3.79521217419770	-0.70241865947715
H	4.07400984401172	2.40722760111143	1.37379993127799
H	-2.94044161752496	1.10576095782921	-0.49181669567318
H	-3.86192007129793	3.07814370781300	-1.69741414113878
H	-2.76635881078262	5.30460362833670	-1.46673105579933
H	-0.74757962459996	5.54676989951289	-0.02401061766631
H	0.16596968529446	3.57996566093980	1.18116757520674
O	-1.93304155010283	-1.19218552135079	0.34803314649937
C	-1.12580867002172	-2.05419060296431	-0.13655574922893
C	-1.76630517196645	-3.29832207476072	-0.69516488031787
C	-3.15344901246231	-3.47710111745764	-0.65105280683058
C	-3.72830659857506	-4.63516029549883	-1.17079561066100
C	-2.92041615621937	-5.62174974406637	-1.73910406103167
C	-1.53573389300471	-5.44811643783070	-1.78643027293508
C	-0.96198304547336	-4.29060598340274	-1.26566911055717
O	0.10801944001363	-1.94410431264501	-0.17474336660187
H	-1.32225936655224	-0.11444429356062	0.77844945470107
H	-3.77020525652964	-2.69545910878344	-0.20309891064502
H	-4.81240994974085	-4.77064252098327	-1.13335159953592
H	-3.37192721620387	-6.53001603311774	-2.14704973777579
H	-0.90239564283694	-6.21993661966181	-2.23166261944621
H	0.11832888881682	-4.13256111050283	-1.29127713008998

Cartesian coordinates of the PBE0-D3/def2-SVP+SMD(THF) optimized geometry of intermediate **14**
$E^{\omega B97M-V}$ = −1528.41163109 hartree
Neutral, Singlet

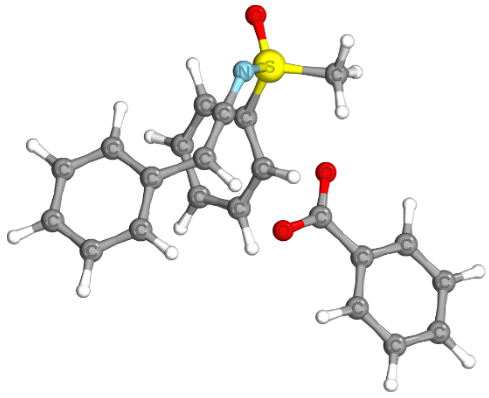

C	2.39965955597618	-0.52402557729029	-2.41130267132234
S	2.03155336824019	-1.85703929401284	-1.33805037307903
C	1.99297336339054	-1.29405875080626	0.32474198290409
C	2.43312721886479	-2.20153467219931	1.29158171442008
C	2.30995024042898	-1.84048941924046	2.62855132498459
C	1.74861410421746	-0.60858370372964	2.97248554443930
C	1.31093292396384	0.27650982888221	1.98711234778554
C	1.42291185333828	-0.05795801056599	0.63988983562015
N	0.45434377881829	-2.29479796540273	-1.80556006189115
C	-0.49505468819716	-1.89498601827085	-1.11132970336574
C	-1.48849193015977	-1.43051045328884	-0.40673903071976
C	-2.08086851662903	-2.00369719522611	0.79946400981930
C	-3.08325146770951	-1.27408359152269	1.45747610838972
C	-3.65196838220630	-1.76099322048931	2.63214291897444
C	-3.22803425792637	-2.97849407122446	3.16432745851654
C	-2.22831802621105	-3.70913401159470	2.51615177285322
C	-1.65738225190362	-3.22859271818975	1.34320360795335
O	2.90944144534467	-3.00596197274111	-1.51090012844888
H	2.37218702279210	-0.95809030326348	-3.42175227482740
H	3.41028659162440	-0.17431469274352	-2.15615322725666
H	1.61669214070429	0.24923744198526	-2.28648585591496
H	2.86594526672383	-3.16015949757972	0.99870560791893
H	2.65361196600138	-2.52625299481994	3.40584816687559
H	1.65023823481097	-0.33613299392616	4.02600955248163
H	0.86800623517818	1.23573820100011	2.26366413155005
H	1.04872791645035	0.60946636397043	-0.14450312697883
H	-1.84309244649710	-0.41683401925698	-0.77350410307191
H	-3.40208025615959	-0.31563588609935	1.04058139540230
H	-4.43070757169601	-1.18308347665188	3.13618427363045
H	-3.67421436644881	-3.35982018395248	4.08621456181402
H	-1.89110617272057	-4.66244547954388	2.93089869649088
H	-0.87307884993046	-3.80339495227008	0.84287891388577
O	-2.17524887085625	1.26373004677755	-1.10903212370611
C	-1.05398452248993	1.71041501517517	-1.44035180285387
C	-0.85651986749102	3.21255036572859	-1.34500495322663
C	-1.88630827268685	4.02813315477802	-0.86296552457172
C	-1.71103161328583	5.40732614342460	-0.76635325929104
C	-0.50088237932488	5.98591439261348	-1.15498463936582
C	0.53075137667715	5.17974925950555	-1.63981798007118
C	0.35163590297133	3.80033489707229	-1.73404162441808
O	-0.07462982625857	1.02268454191920	-1.82457641908045
H	-2.82389443560559	3.55192960129973	-0.56782254037443
H	-2.52063459209724	6.03677044892963	-0.38680379617602
H	-0.36172690269846	7.06785449069655	-1.08036077124862
H	1.47829821973029	5.63114809334534	-1.94653937585339
H	1.14620340434778	3.15370548367672	-2.11306654231599

Cartesian coordinates of the PBE0-D3/def2-SVP+SMD(THF) optimized geometry of **TS2**
$E^{\omega B97M\text{-}V}$ = −1528.41141877 hartree
Neutral, Singlet

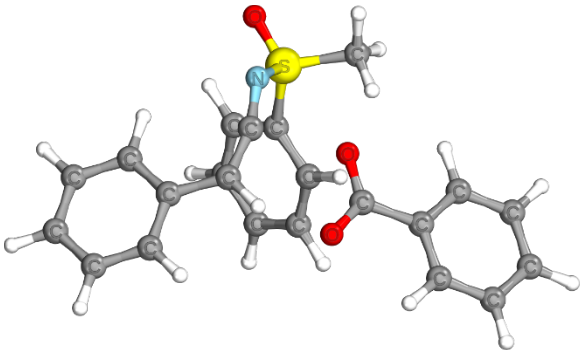

C	2.18663689764026	-0.33600464730903	-2.32016767895830
S	1.74691645419699	-1.60334256032247	-1.19059523920005
C	1.83446634971408	-0.96679524822264	0.44926637024320
C	2.34467471234097	-1.82753502982896	1.42279546114968
C	2.36365226169921	-1.38506630465841	2.74151017601243
C	1.87571587453234	-0.11690427014229	3.06221672699030
C	1.36489046901636	0.72230333190145	2.07133260281297
C	1.33352878194036	0.30324944178811	0.74434965260481
N	0.15650983044279	-1.92319345559141	-1.59968834397062
C	-0.77147874366969	-1.39384286036836	-0.95001524542865
C	-1.80416057577402	-1.18724225165642	-0.16375110576628
C	-2.23542606080621	-2.12859597322611	0.87417538178320
C	-3.34120293107555	-1.78252150296810	1.66677383375158
C	-3.78060692369019	-2.63243686265827	2.67928685347538
C	-3.12376898144377	-3.83944637776087	2.91626540186663
C	-2.02264690213686	-4.19271867219317	2.13176819944154
C	-1.58017525140569	-3.34804298676443	1.12004139202481
O	2.54199746242322	-2.81526273116104	-1.33883731564166
H	2.16386047887595	-0.81567697485372	-3.30970560183964
H	3.19695053238267	0.01085483716766	-2.06285802105381
H	1.40592353035511	0.44095053818876	-2.24258228170538
H	2.72319811893318	-2.81401887515865	1.14790857938070
H	2.76355348664240	-2.03559714871589	3.52222770756698
H	1.89311840436253	0.22157469911290	4.10092083467754
H	0.98013026518680	1.71137255716401	2.32877691272847
H	0.90863572354643	0.93384516351233	-0.03999015559699
H	-2.33086571142192	-0.22508851550733	-0.31507872762911
H	-3.85372646144680	-0.83497459581353	1.48192651871572
H	-4.64283544298326	-2.34794775677757	3.28777115470920
H	-3.46747677028982	-4.50584964524986	3.71127615116473
H	-1.50298220801930	-5.13727624408184	2.31177415305875
H	-0.71705335884791	-3.63541959993380	0.51406940665639
O	-2.48191890366532	1.57524137891487	-0.93247431583658
C	-1.32469008890103	1.73419560793827	-1.35353679466250
C	-0.78422164767596	3.15354297979181	-1.40155010696755
C	-1.59330552047545	4.20969031958904	-0.96722735556265
C	-1.12150843041241	5.52041724497800	-0.98750220988401
C	0.16987616673544	5.79022370382599	-1.44526116735603
C	0.98362489979077	4.74396330545425	-1.88256387524434
C	0.50715977683354	3.43348130028167	-1.86153534400243
O	-0.53735013517792	0.81310614085982	-1.72708887032830
H	-2.59879694090609	3.97159517705629	-0.61311339342679
H	-1.76175937752662	6.33773646223916	-0.64445844371582
H	0.54247918658750	6.81799854581666	-1.46191989566133
H	1.99448858641336	4.95153990359309	-2.24372730034049
H	1.13960130905993	2.61266906544993	-2.20615422603608

Cartesian coordinates of the PBE0-D3/def2-SVP+SMD(THF) optimized geometry of (*E*)-adduct **11**

$E^{\omega B97M\text{-}V}$ = −1528.46725408 hartree

Neutral, Singlet

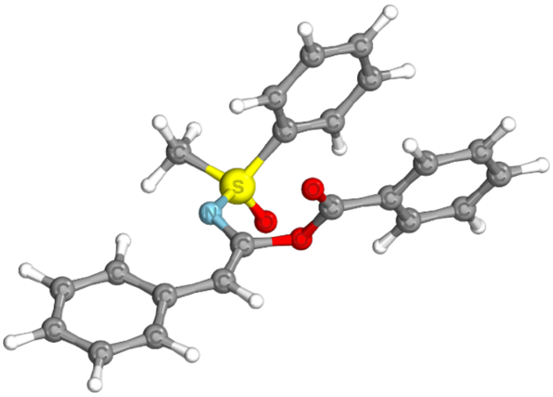

C	-1.14459662747216	-2.74422051408481	2.06377663238236
S	-0.30491674570053	-1.27050385260643	1.57122932394122
C	1.00188893752534	-1.83508290380138	0.48756308227968
C	0.69782615027278	-2.20139452521431	-0.82305957654982
C	1.73533047467434	-2.61119421969892	-1.65660932773138
C	3.04590724523429	-2.65083758999488	-1.17685882341171
C	3.32899819071285	-2.28774728228535	0.13997358643248
C	2.30193785446274	-1.87442240620213	0.98601387021887
N	-1.41739035145492	-0.52121720081288	0.76063573468820
C	-1.16880252401425	0.70257164414868	0.20085867948568
O	0.17875565605877	1.04805369607727	0.11441375748339
C	0.83109837328940	0.97916905214732	-1.07342940423189
C	2.29427710135500	1.13291557898400	-0.88889044797191
C	2.86446612053923	1.34545627629606	0.37252981626717
C	4.24733699495162	1.44304399179985	0.49797577689878
C	5.06247878233164	1.32094327861464	-0.62859258532645
C	4.49563373486231	1.10446757383717	-1.88618101119614
C	3.11356939698383	1.01241310595947	-2.01737163192769
O	0.27894167769456	0.79880264799157	-2.12503141058983
C	-2.09202592578146	1.58938340031647	-0.23400996249324
C	-3.54405799630770	1.48223771751759	-0.19462208562636
C	-4.24845268681177	0.36102044880623	0.29103138789242
C	-5.64078803032278	0.34056098639173	0.29450108201769
C	-6.37374779621581	1.42832304265130	-0.18312920281283
C	-5.69234366805826	2.54558133498687	-0.66925674468124
C	-4.30136397518656	2.57043976449730	-0.67522527345052
O	0.32653091293615	-0.64178674995187	2.74251355055276
H	-0.41775676794248	-3.36786994423214	2.60034986567453
H	-1.53993221844063	-3.25732504719926	1.17840352209763
H	-1.95711610808185	-2.42371031301607	2.73071369950454
H	-0.32870148968989	-2.14385499962183	-1.19194691721597
H	1.51924501020819	-2.88999793623256	-2.69036992594271
H	3.85630075547607	-2.96392414360855	-1.83955640092232
H	4.35674547570785	-2.31306374348957	0.50915900734570
H	2.49996881774233	-1.56674625596415	2.01421367801872
H	2.22073986785765	1.41208488481740	1.25068796617188
H	4.69311091727222	1.60751333377935	1.48188987078464
H	6.14834772683624	1.39260308382656	-0.52557978829868
H	5.13519391930253	1.00572850246459	-2.76649337359387
H	2.64981735586563	0.83892241870007	-2.99068244687930
H	-1.68948076648034	2.50205205362123	-0.67821142545461
H	-3.68555259760427	-0.49427891025804	0.66601165554578
H	-6.16212340644484	-0.54184173629812	0.67619802493409
H	-7.46646913302451	1.40518057694224	-0.17797839059365
H	-6.25003085534862	3.40634289789162	-1.04831991997454
H	-3.77708458746991	3.45025992210664	-1.05932678304155

Cartesian coordinates of the PBE0-D3/def2-SVP+SMD(THF) optimized geometry of **TS2'**
$E^{\omega B97M\text{-}V}$ = −1528.39758188 hartree
Neutral, Singlet

C	1.20443319209084	-4.14409692778371	-0.42949873746055
S	1.11560967287611	-2.49468578563918	0.18013635828331
C	2.37189601311192	-1.55671292306779	-0.62547144306975
C	2.61574702502658	-1.74546301570545	-1.98847976835264
C	3.61141401736124	-0.97964171105791	-2.58771189615394
C	4.32989800886230	-0.05288885222553	-1.83082407463654
C	4.05103739069242	0.12802861775753	-0.47571700485055
C	3.05359967656115	-0.61658528575692	0.14840727155037
N	-0.42269716312863	-2.07818952245196	-0.34536919507781
C	-0.59961098854976	-1.00759569599598	-0.96066296300837
O	1.48744927386514	0.51876688398431	2.64903743242208
C	0.44519452471769	0.38163287334121	1.99322632432176
C	-0.86516060244300	0.30390873441514	2.76298601388595
C	-2.09088992607621	0.11483365308548	2.11570769726740
C	-3.27665946796958	0.03958350849599	2.84643030107833
C	-3.24938048411751	0.15468900045950	4.23699345879955
C	-2.03058199442007	0.34335718509323	4.89265445601240
C	-0.84897498594360	0.41531238963428	4.15804173871196
O	0.37766206523105	0.30900660626983	0.72818415405366
C	-0.95247075450784	-0.18933692025766	-1.92927840717130
C	-1.35194117437864	1.21361079918342	-2.01613410187722
C	-1.25715111633834	2.12161310068613	-0.94889157360644
C	-1.67824525834064	3.43746731819459	-1.11456152371686
C	-2.19620962582754	3.87203732768088	-2.33625629740594
C	-2.28388055798637	2.97851853811861	-3.40427687124830
C	-1.86346804059881	1.66115874073316	-3.24719930855289
O	1.22399864636699	-2.48306361925553	1.62997045828873
H	2.18475269856346	-4.54429739898588	-0.13709398127235
H	1.06177260813992	-4.16458981630962	-1.51722539683872
H	0.38991593766774	-4.67919134193531	0.07898618034438
H	2.05259220467432	-2.46792438179203	-2.58221987925734
H	3.82443023509860	-1.11009030395504	-3.65078085644354
H	5.11259760473248	0.54248461804253	-2.30737579011410
H	4.60515415403800	0.86975703142426	0.10404468979988
H	2.77303900679069	-0.43366679072546	1.19267946017091
H	-2.10927300602210	0.02903180864766	1.02794621973694
H	-4.22813149973896	-0.10921335521643	2.32844721270717
H	-4.17833611140224	0.09655908842485	4.81076837188558
H	-2.00459162446988	0.43333011247343	5.98224263745670
H	0.11865207503628	0.55863028921536	4.64425599857122
H	-0.97063501143485	-0.75187199919536	-2.87501423044868
H	-0.81386876320689	1.77209274839964	-0.01446793068720
H	-1.59696074798481	4.13540777316578	-0.27707363060019
H	-2.52603557125239	4.90704074920637	-2.45744611539048
H	-2.68188858907354	3.30904610553430	-4.36701496968603
H	-1.93380296629272	0.96220004564529	-4.08510048842051

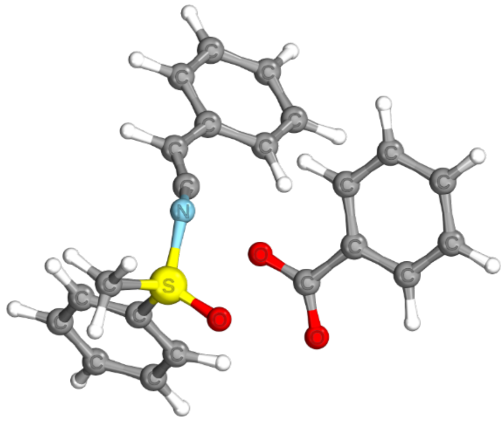

Cartesian coordinates of the PBE0-D3/def2-SVP+SMD(THF) optimized geometry of (*Z*)-adduct **12**

$E^{\omega B97M\text{-}V}$ = −1528.46693332 hartree

Neutral, Singlet

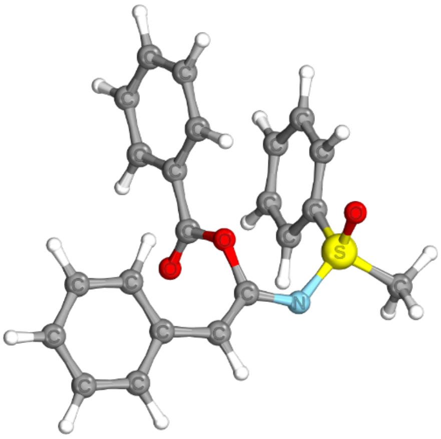

C	2.65965766677419	-2.93971410038919	1.95673365484083
S	1.59072141058941	-1.56626034903825	1.65356734032471
C	2.46497253853146	-0.58580123160782	0.43646232972297
C	2.54417303276360	-1.03827140773951	-0.88036588300684
C	3.21262547863589	-0.25226706128553	-1.81526055617496
C	3.78385468957100	0.96219287646632	-1.42972079482186
C	3.69739410850578	1.39625118749249	-0.10693506221981
C	3.03409636314107	0.61903423332876	0.84089924733003
N	0.33713728137470	-2.26287983488396	1.02739192990195
C	-0.76782379447842	-1.52331492774013	0.68383727882617
O	-0.53310300463257	-0.17681180917244	0.42653979748783
C	-0.34954446921023	0.22871575834383	-0.85753179741172
C	0.06562546264265	1.64941049453231	-0.92146702545382
C	0.34304442490373	2.19445861615717	-2.18074325927301
C	0.77318289583570	3.51291415038796	-2.28812582974103
C	0.92495186268835	4.29131866788375	-1.13879984069175
C	0.64715287366762	3.75099403745588	0.11773944542066
C	0.22113130844701	2.43049314736015	0.23058886855771
O	-0.49932827526512	-0.49680714344645	-1.80296639929354
C	-2.00827376306537	-2.05099393338597	0.60043659892491
C	-3.26244437556368	-1.43195891418232	0.18485915421897
C	-4.33980619888016	-2.28470086010605	-0.13098315871898
C	-5.57190651986392	-1.78201632526832	-0.53777825883957
C	-5.77217838782085	-0.40444131209690	-0.63454664496869
C	-4.72403690541947	0.45712491791968	-0.30783245496465
C	-3.48950711004292	-0.04275676242480	0.09741053122438
O	1.46611438146290	-0.74743009921430	2.87100393365723
H	3.60080468117908	-2.53681219283134	2.35297859854015
H	2.82073628017533	-3.49697497082510	1.02560153225706
H	2.15002108344463	-3.56201810512651	2.70534699904440
H	2.06869399530489	-1.97651608857752	-1.17465630062784
H	3.27606318295701	-0.58468933567047	-2.85381017724548
H	4.29716359127502	1.57930795652970	-2.17108809242168
H	4.13926090330991	2.35045814070303	0.18895542054977
H	2.93923358113884	0.94192109170777	1.87917718708639
H	0.22184262586586	1.56716276633783	-3.06613468361977
H	0.99260550083389	3.93707850442107	-3.27081943382699
H	1.26387909268768	5.32706813984502	-1.22331984451898
H	0.76983970515897	4.36065194818544	1.01600899279559
H	0.02089730235859	1.99463967082612	1.21022564374614
H	-2.05606521557275	-3.11438659243152	0.84705886301156
H	-4.19450211119539	-3.36618273142398	-0.05777855865318
H	-6.38352098473450	-2.47312756249618	-0.78089514592981
H	-6.73820066039664	-0.00535659361368	-0.95370683761894
H	-4.86913844846473	1.53949492346959	-0.36300025391471
H	-2.70293746241800	0.65982763692436	0.37142933288821

Cartesian coordinates of the PBE0-D3/def2-SVP+SMD(THF) optimized geometry of **TS3**

$E^{\omega B97M\text{-}V}$ = −1528.35917485 hartree

Neutral, Singlet

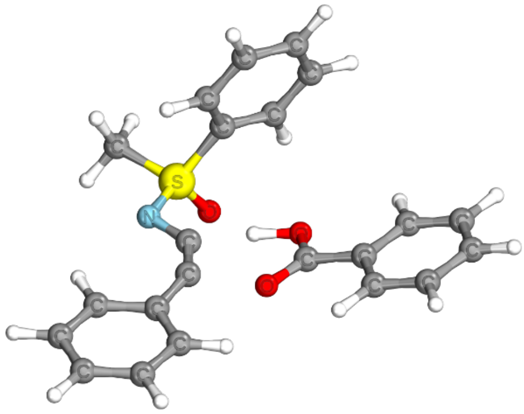

C	0.55615559817660	-4.26912501299822	2.48002946628699
S	0.79459766006895	-2.56392844368652	2.09699217467255
C	1.80125256136711	-2.51451178782098	0.62133005492710
C	3.12656017471901	-2.10423649816095	0.73919145707908
C	3.90243787064222	-2.01423718319987	-0.41505084216715
C	3.34745016797870	-2.32772814652090	-1.65593742445288
C	2.01476731522308	-2.73340867635098	-1.75489602596439
C	1.22636782819469	-2.82625095069232	-0.61137200035451
N	-0.68253514564274	-2.09118551103032	1.75256020891203
C	-0.74023501882289	-0.82639252049918	1.22794747708282
O	-0.83830384159803	1.60242255999758	0.06676752462637
C	0.40076897687237	1.64525297166859	-0.04155751645976
C	1.04202555118826	2.68970065811202	-0.87842785157746
C	0.25222678512809	3.66538699242626	-1.49811405515777
C	0.85138839277793	4.64328010593860	-2.28603411767723
C	2.23725895494092	4.64814542957737	-2.45769768200626
C	3.02584552084375	3.67521779990151	-1.84077726887498
C	2.43135108396167	2.69605614946866	-1.04994814619813
O	1.17815782128519	0.80552512066843	0.54602509209982
C	-1.64713392993438	-0.01244386904987	0.84476954284251
C	-3.03694313839022	0.31879415530795	0.69062860410029
C	-3.58836414770152	1.52882148795507	0.23851742136465
C	-4.97053853014215	1.68778416207553	0.16765282091147
C	-5.83109090346016	0.65598933730086	0.53960112011007
C	-5.29206622049745	-0.55120485706002	0.99084855918427
C	-3.91503277390978	-0.72261157495268	1.06771151210026
O	1.55296925038187	-1.90224358295568	3.17118836188845
H	-0.13705727908818	-4.29289349472283	3.33255270256049
H	1.53338858710634	-4.68545600653457	2.75618945741732
H	0.12730040350330	-4.78618270716566	1.61263826953662
H	3.53000141063993	-1.84923583814681	1.72097038621706
H	4.94396117275777	-1.69216797680582	-0.34396643484027
H	3.95848395192778	-2.25068402272600	-2.55861190164353
H	1.58345420590146	-2.97022692461574	-2.73021132694433
H	0.17722049438715	-3.12389223287250	-0.67559759373360
H	-0.83050970044440	3.64814866837859	-1.35328347814269
H	0.23665719790313	5.40624732322273	-2.76955312777210
H	2.70626395698977	5.41660980376726	-3.07760641065863
H	4.10984005156839	3.68062144627625	-1.97817385027445
H	3.03507271589560	1.92705550826279	-0.56395748256079
H	0.49513370116778	0.03661463217761	0.92012981055905
H	-2.92743837874561	2.34258207421252	-0.05195083825906
H	-5.37803182433964	2.63953562892073	-0.18361128415303
H	-6.91400146224393	0.78976863979700	0.48029314981968
H	-5.95127913097912	-1.37126735756238	1.28780302699883
H	-3.49779793755864	-1.66804547928314	1.42399845857519

Cartesian coordinates of the PBE0-D3/def2-SVP+SMD(THF) optimized geometry of (*E*)-adduct **13**
$E^{\omega B97M\text{-}V}$ = −1528.45780942 hartree
Neutral, Singlet

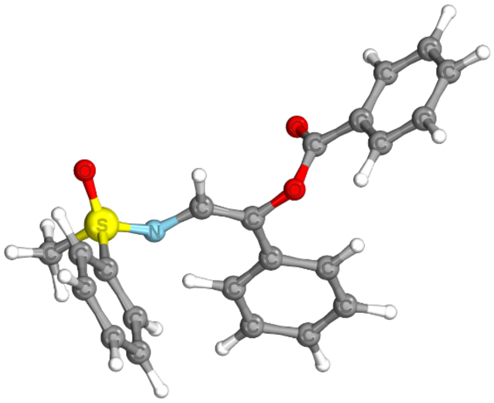

C	4.11604180850953	0.40649590382999	-2.33894392644325
S	2.88984584259234	-0.57776733193122	-1.53880324154048
C	3.58798752418282	-0.98936270916241	0.05711284931834
C	3.53814292504037	-0.04791648383129	1.08520857986929
C	4.07758750942417	-0.38546678932654	2.32394381232928
C	4.64827879014342	-1.64461841265614	2.51939734984708
C	4.68216337923542	-2.57602999659495	1.48094339862467
C	4.14789148057616	-2.25283038372005	0.23510596108176
N	1.70646968603473	0.42869303185138	-1.35008746265325
C	0.52174598818208	-0.07929864202390	-0.86663341701931
O	-1.59594153882668	-0.07555966076876	0.08345980714417
C	-2.66082642525275	-0.17834265091654	-0.73720933354138
C	-3.78072397332330	-0.91785093393112	-0.09805571017956
C	-4.95177839957743	-1.10520556455561	-0.84227489438918
C	-6.02850436846181	-1.78981173111309	-0.28788601728271
C	-5.94060257156568	-2.29000898521659	1.01279195502140
C	-4.77515680786021	-2.10468951637108	1.75791471765767
C	-3.69493916763183	-1.42012720756336	1.20664823732780
O	-2.68963105179662	0.27903407275501	-1.85006396716396
C	-0.49373358616325	0.66055457662400	-0.35984480513029
C	-0.58229704407795	2.10361967225993	-0.14394247418853
C	-1.67771291953212	2.64295581175902	0.55861286610922
C	-1.78163501550059	4.01237037742137	0.78429618717753
C	-0.79690431618775	4.88344856071849	0.31780038715195
C	0.29474927637619	4.36316429560139	-0.37890130749835
C	0.40618398966369	2.99485665728413	-0.60915213767164
O	2.69180718536068	-1.84423629257814	-2.26423520759748
H	4.32067357621702	1.29937228986354	-1.73518540617100
H	3.69568797913560	0.67891280543972	-3.31728943168838
H	5.01266720414278	-0.21614316426593	-2.45486595500033
H	3.07484794589133	0.92774209155349	0.91917697229047
H	4.04633210099980	0.33707672916326	3.14274012851362
H	5.06706221222201	-1.90427269355517	3.49478373840602
H	5.12340122744713	-3.56255850619345	1.64144877858098
H	4.15150270622633	-2.96815425345192	-0.58975657557379
H	-5.00035256023453	-0.70572283910194	-1.85742640020296
H	-6.94126758641595	-1.93494279164375	-0.87077959639530
H	-6.78656326039133	-2.82791018523847	1.44853342185398
H	-4.70779187501966	-2.49628856968698	2.77581179531624
H	-2.78112870101719	-1.27121023852841	1.78390469253246
H	0.34256768317437	-1.16317302566929	-0.90522056304039
H	-2.45493301573450	1.97978587483820	0.94236031391270
H	-2.64310868897392	4.40170128335762	1.33342610217505
H	-0.88004718269866	5.95873434679755	0.49467250119779
H	1.07302120679400	5.03333990197582	-0.75412656188212
H	1.26022544087173	2.59740370540215	-1.15744027698586

Cartesian coordinates of the PBE0-D3/def2-SVP+SMD(THF) optimized geometry of benzoate anion (**15**)
$E^{\omega B97M\text{-}V}$ = −420.40028627 hartree
Monoanion, Singlet

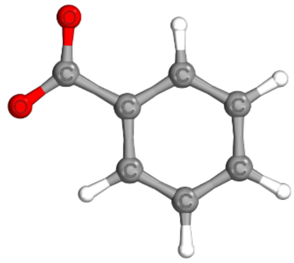

O	-2.29847909754814	1.12480085221677	0.00943786387925
C	-1.75961470777456	0.00000269523032	0.00016164345424
O	-2.29847797203063	-1.12478921454865	-0.00976279547851
C	-0.21623792851073	0.00000128706114	0.00009064207345
C	0.49761483192643	-1.20269821664107	-0.00606450133543
C	1.89262074741049	-1.20774682143473	-0.00601115766961
C	2.59486756985462	-0.00000094760951	-0.00010778688942
C	1.89262130010090	1.20774492819742	0.00589421187019
C	0.49761560533113	1.20269863082580	0.00614949035207
H	-0.08356667464695	-2.12862482101028	-0.01071684276644
H	2.43906911334186	-2.15584090636251	-0.01071000214859
H	3.68866375846535	-0.00000052097972	-0.00018669273100
H	2.43907125542957	2.15583830139167	0.01050638867481
H	-0.08356007124936	2.12862804966336	0.01086947021498

Cartesian coordinates of the PBE0-D3/def2-SVP+SMD(THF) optimized geometry of **TS1'**
$E^{\omega B97M\text{-}V}$ = −1528.83409956 hartree
Monocation, Singlet

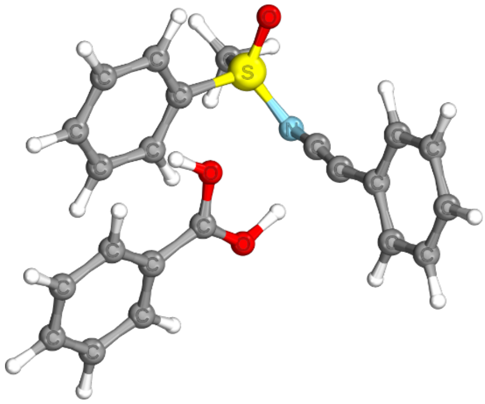

C	3.20136867855130	-0.02320767703656	3.87774280873101
S	2.40824584211724	0.76497368876515	2.52075078885954
C	2.47589078528570	-0.37612066664303	1.15565070933332
C	1.79451226973357	-1.59281197378062	1.23708668521568
C	1.85328407356668	-2.45601966386039	0.14676941712867
C	2.56904423504668	-2.09351400952160	-0.99824051719617
C	3.23272135094777	-0.86843083970876	-1.06137526887516
C	3.19550750952415	0.00441917588372	0.02652195980541
N	0.89389215857250	0.85014055338544	3.02696840734904
C	0.04800456774231	1.49758966695628	2.26356701535825
C	-0.83358094752800	2.01022225674754	1.56686674248739
C	-1.70624689036879	2.91587566857255	0.87296771829490
C	-3.07134909450747	2.61787328070492	0.71074519496464
C	-3.90581381834421	3.49722901452524	0.02647647012689
C	-3.39427501839603	4.67755237029411	-0.51334053745897
C	-2.03919682042001	4.97774460785899	-0.36235263553101
C	-1.19795531238589	4.10817988914322	0.32440960685105
O	3.09581363887594	1.99936492860670	2.13650839452661
H	3.11553818484483	0.67501500392897	4.72272990756832
H	4.25076938897929	-0.17644320285133	3.59380460095818
H	2.69672939318722	-0.97248917004372	4.09808612530705
H	1.21944053136716	-1.85817135085255	2.12762407216604
H	1.33084418096850	-3.41415960968091	0.18679364735063
H	2.60446248942834	-2.77534631090120	-1.85113553862097
H	3.78880811603319	-0.58851318088776	-1.95898430507665
H	3.70851387129596	0.96786094144433	0.00295113981480
H	-3.47655070570016	1.69533593286194	1.13329543200978
H	-4.96596610954841	3.25698126437673	-0.08657036797719
H	-4.05165096571213	5.36467626355668	-1.05175134353565
H	-1.63284360239000	5.90134872181292	-0.78232158764381
H	-0.13783457286688	4.34207936555742	0.44407879587800
O	-1.32922289520992	-0.39755256597702	0.39322157639347
C	-0.75156968342247	-0.58673807620613	-0.71467483933506
C	-1.02727881935833	-1.79411258437752	-1.47161697747467
C	-1.73259434322919	-2.83423599558678	-0.84222534036652
C	-1.97635762176518	-4.01233787395815	-1.53354992279811
C	-1.52922154802107	-4.15395525716525	-2.84897210564076
C	-0.84015297388655	-3.11682642024412	-3.48181472265832
C	-0.58524644680422	-1.93656225094783	-2.79795228770552

O	0.06780323152456	0.32844600541291	-1.11684395727595
H	-1.03678877335530	0.48429537333434	0.89344070865958
H	-2.07012189570078	-2.71564429611333	0.18862853949542
H	-2.51612752197381	-4.82664355989087	-1.04615320612157
H	-1.72307663918244	-5.08300686664537	-3.39060039170210
H	-0.50464742861536	-3.22812715512370	-4.51460776757866
H	-0.06332505329740	-1.13308262920104	-3.32545627338023
H	0.54780100439713	0.07684921347538	-1.92714657068060

Cartesian coordinates of the PBE0-D3/def2-SVP+SMD(THF) optimized geometry of phenylacetylene **23**
$E^{\omega B97M\text{-}V}$ = −308.40623404 hartree
Neutral, Singlet

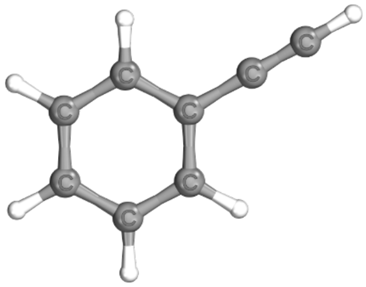

C	3.21455954284638	0.00023098545308	0.00009775072880
C	1.99983879040461	-0.00025087847007	0.00001270164183
C	0.56917765448760	-0.00013575472516	-0.00000688068958
C	-0.14151089567518	-1.21367626592098	-0.00005279439687
C	-1.53299337263805	-1.20806598760447	-0.00006830525814
C	-2.23165836289865	0.00010172805885	-0.00003804490402
C	-1.53279384656787	1.20814660965354	0.00000797748021
C	-0.14130675975292	1.21351693645466	0.00002256794185
H	4.29234412736523	-0.00051703721282	-0.00003840974487
H	0.40826621912346	-2.15737283112227	-0.00007067620617
H	-2.07769777865316	-2.15545283697827	-0.00010403574547
H	-3.32451937907132	0.00017341933011	-0.00004910792311
H	-2.07730751057589	2.15564326807488	0.00003430625206
H	0.40861772050576	2.15712755350891	0.00006020782349

Cartesian coordinates of the PBE0-D3/def2-SVP+SMD(THF) optimized geometry of **TS1**[D1]
$E^{\omega B97M\text{-}V}$ = −729.20670444 hartree
Neutral, Singlet

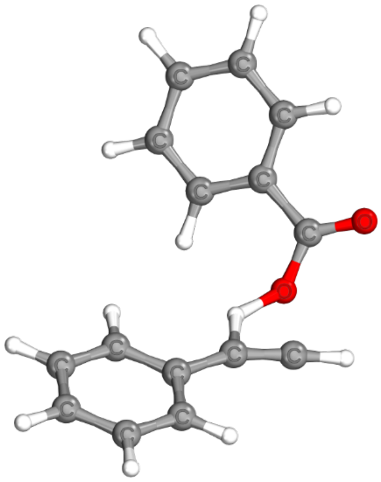

C	0.84581265257003	-2.58526028332828	-0.33745078629198
C	-0.25293548829617	-1.97894751560669	-0.41818431443940
C	-1.69518794146233	-1.92335597489551	-0.35373918340063
C	-2.36240969200530	-0.70949800138511	-0.57744500321036
C	-3.75158629762659	-0.65753030152534	-0.51172709777712
C	-4.48143538871255	-1.81101932314828	-0.22358206013540
C	-3.82138439070234	-3.02148321846319	0.00042807797247
C	-2.43397342062934	-3.08299253981314	-0.06292565593225
H	-1.78346701155023	0.18980117169631	-0.79980654072623
H	-4.26776265058268	0.28941407882762	-0.68616418409371
H	-5.57226434393545	-1.76787971011175	-0.17265211214049
H	-4.39377478804368	-3.92435568161054	0.22644252054927
H	-1.91054468132674	-4.02576901521158	0.11104278196792
C	3.03584650988212	3.26063009056440	1.42476892879712
C	2.15521273601068	3.97851293506413	0.61272399355470
C	3.21416026909933	1.89502085137565	1.22011729292216
C	1.45557411310053	3.32620033791907	-0.40335667658131
C	2.50817114557318	1.23217834853245	0.20977991691657
C	1.63059144403845	1.95775950423588	-0.60399161626532
C	2.72249626926343	-0.24904891545287	0.03158913654648
O	1.93812425732604	-0.82371607144648	-0.83255283438658
O	3.57324659055653	-0.84137846874399	0.67645163727591
H	0.53148324896685	-1.02509384334466	-0.67938851044480
H	3.58571627778222	3.77038582083382	2.22023289210291
H	2.01570645766029	5.05115790956233	0.77135421218803
H	3.90012966061887	1.31312328194309	1.83960345344701
H	0.77103909836174	3.88773495086537	-1.04443578249279
H	1.09239252377266	1.44404264439301	-1.40330796558827
H	1.75102284029046	-3.16863306172572	-0.23382452033391

Cartesian coordinates of the PBE0-D3/def2-SVP+SMD(THF) optimized geometry of adduct **24**
$E^{\omega B97M\text{-}V}$ = −729.33135959 hartree
Neutral, Singlet

H	-0.82211220889404	-1.38056181799714	-0.00120167557706
C	-0.68585012682125	-0.29720521942513	-0.00082766687005
O	0.61402343904822	0.11667913214161	-0.00079981062345
C	1.59583397621137	-0.81461299581118	0.00046309779386
C	2.94251509290958	-0.19779013604196	0.00032509273535
C	3.13058641509229	1.19074427597198	-0.00206914673101
C	4.42067183156418	1.71381865877952	-0.00212930357643
C	5.52360122193132	0.85845289257477	0.00024586922536
C	5.33854918798844	-0.52548191983478	0.00265733989815
C	4.05188538813377	-1.05339228009996	0.00266431398334
O	1.37663331362544	-1.99925798588766	0.00152109742599
C	-1.66431592632608	0.61661792281514	-0.00037035980883
H	-1.37501265490760	1.67248459816295	0.00038448127963
C	-3.09999465710454	0.33456660474789	-0.00040068439170
C	-3.99802947782451	1.41547180742098	0.00325593213094
C	-5.37564941125468	1.20694228145933	0.00368184288487
C	-5.88751569892576	-0.08966337348350	0.00040485725892
C	-5.00718308243411	-1.17505504383061	-0.00339263961451
C	-3.63213514593034	-0.96866638022035	-0.00385554309389
H	2.26603309681484	1.85606568643316	-0.00387663145184
H	4.56720661896599	2.79655242043069	-0.00402964882424
H	6.53485892700219	1.27329554838600	0.00022012859494
H	6.20222557128361	-1.19465043011419	0.00453098846598
H	3.88673736754176	-2.13279197567460	0.00449227089963
H	-3.60176275077825	2.43468817853726	0.00587136717894
H	-6.05354831290330	2.06446356687617	0.00658826090147
H	-6.96751327857727	-0.25689407803040	0.00070792432956
H	-5.39851717918114	-2.19575859211363	-0.00613888301389
H	-2.96415666325012	-1.83307539277236	-0.00708067851004

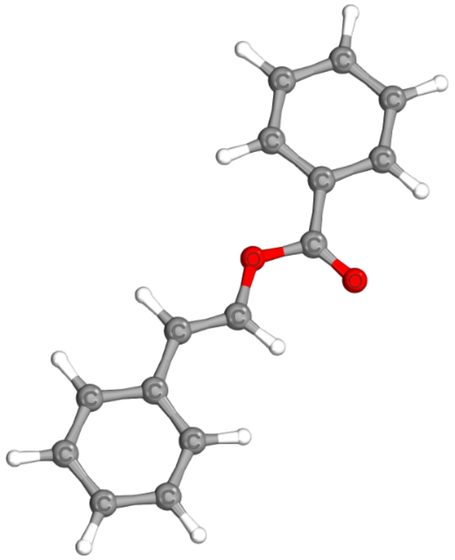

Cartesian coordinates of the PBE0-D3/def2-SVP+SMD(THF) optimized geometry of alkyne **25**
$E^{\omega B97M\text{-}V}$ = −422.93200030 hartree
Neutral, Singlet

C	-4.16845462730310	0.35510997110150	-0.48157972352285
O	-3.33010673300662	-0.79357625490019	-0.29394915756997
C	-2.06351439362743	-0.57055449471016	-0.24158171705285
C	-0.86006642787785	-0.40157686770353	-0.19132887090155
C	0.55331910537829	-0.20662673995973	-0.11909954958682
C	1.42160835372248	-1.30263868009537	0.04612905214969
C	2.79778353893312	-1.10756270127961	0.11762410930991
C	3.33583184476126	0.17691979191413	0.02585467537266
C	2.48351537173316	1.26979276586368	-0.13852349510503
C	1.10594550666114	1.08515790340512	-0.21093323042887
H	-4.03920379540727	1.06103938417996	0.35264169319433
H	-5.19978891432292	-0.01565068329110	-0.50254744215717
H	-3.92718040151235	0.85028251782774	-1.43416679388916
H	1.00428197350031	-2.30952042315284	0.11856449710959
H	3.45747076383014	-1.96959735591774	0.24651567086176
H	4.41699292020246	0.32583069347236	0.08219974804375
H	2.89574774956109	2.27957688762101	-0.21173916322046
H	0.44215172357409	1.94312797312477	-0.33954715470696

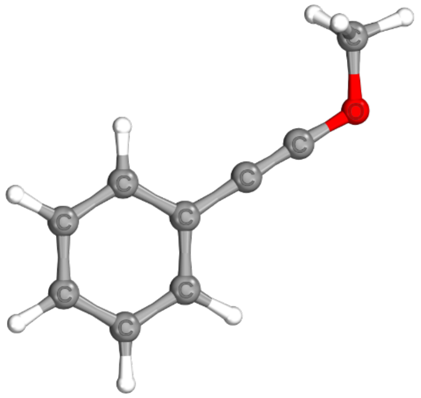

Cartesian coordinates of the PBE0-D3/def2-SVP+SMD(THF) optimized geometry of alkyne **TS1**^{D2}

$E^{\omega B97M-V}$ = −843.78373129 hartree

Neutral, Singlet

C	0.03177091655231	-1.91912617388701	0.24542018003764
C	-1.04047155395517	-1.24299438000867	0.12091578361594
C	-2.40207872883546	-1.56640514413627	-0.30273632334509
C	-3.34415840046436	-0.53256383087001	-0.41430158369270
C	-4.65012033630632	-0.80721073299882	-0.81459057668852
C	-5.03298414099166	-2.11565567856473	-1.10768940753675
C	-4.10214430334385	-3.15160496496284	-0.99885835894794
C	-2.79715170563573	-2.88209663663562	-0.60045745237766
H	-3.04425432809511	0.49285022075582	-0.18574622870476
H	-5.37356198614421	0.00778216536568	-0.89725149434631
H	-6.05767300358913	-2.33063661708671	-1.42110913411552
H	-4.39687912092967	-4.17919192336565	-1.22652980193916
H	-2.07299341362221	-3.69653147106793	-0.51498557471369
C	4.24638977713607	2.94814914126994	0.84852122376305
C	3.74307753267676	4.24954045921324	0.79574472441736
C	3.37438218505083	1.86267383824024	0.80801756074930
C	2.36649656510538	4.46317665340919	0.70350314756997
C	1.99444684158079	2.07183278231519	0.71540899891787
C	1.49433034274327	3.37736799219219	0.66463447127597
C	1.05986618146969	0.89649621272168	0.67181258395976
O	-0.18555998523059	1.15296508263133	0.63097325403844
O	1.53474278837988	-0.25622454280740	0.67700806614183
H	-0.72528384955880	-0.04279579180336	0.40875972802405
H	5.32418871732836	2.78125310776439	0.92200436959157
H	4.42750362323746	5.10147936816721	0.82697102410924
H	3.74782298106616	0.83719682842685	0.84972773176761
H	1.97256106269154	5.48197481694068	0.66188351057692
H	0.41460402716189	3.52445850402322	0.59366317240439
O	0.92258082827113	-2.77478425627586	0.40811241691076
C	1.96268429313338	-2.91044779707866	-0.60130628468508
H	2.51136408946892	-3.82086949468149	-0.33978058118995
H	2.60074851017980	-2.02215046309530	-0.53676799660804
H	1.49575359346864	-2.99790727411052	-1.59097114898050

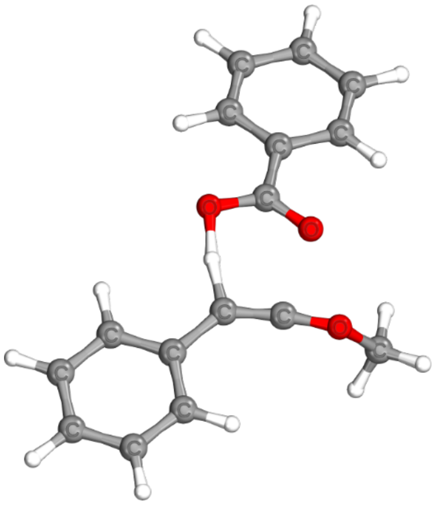

Cartesian coordinates of the PBE0-D3/def2-SVP+SMD(THF) optimized geometry of adduct **26**

$E^{\omega B97M-V}$ = −843.87129074 hartree

Neutral, Singlet

C	0.60901024519941	-0.41716397192272	-0.02617236141442
C	1.51587521752729	0.46857872146048	-0.47849670063491
C	2.96796997116419	0.43798461794854	-0.34303945042113
C	3.68385357164956	-0.56082974472152	0.34703918884480
C	5.07368958905523	-0.51639165399714	0.42257055487664
C	5.78943458081648	0.51948427171932	-0.17942752705315
C	5.09520701840684	1.51855924223932	-0.86312461656590
C	3.70664872773881	1.47707479479866	-0.94304533280379
H	3.14234451485330	-1.37446942679296	0.82885717392499
H	5.60534566191589	-1.30383487128197	0.96380457494679
H	6.88002042943126	0.54893612565739	-0.11429880200382
H	5.63998116120346	2.33839592727954	-1.33897023191209
H	3.17118936361601	2.26425916124459	-1.48130848955199
C	-4.68194832258768	-0.12835789629946	-1.48844045637581
C	-5.63985091331394	0.35623034257976	-0.59651103060411
C	-3.34243793036050	-0.18148532607627	-1.11334170922883
C	-5.26096583554708	0.78971347518254	0.67564124542087
C	-2.96072265819634	0.25308113257654	0.16263891163029
C	-3.92448982107368	0.73831296388028	1.05606388292812
C	-1.55436938111917	0.21557827652664	0.62075865690616

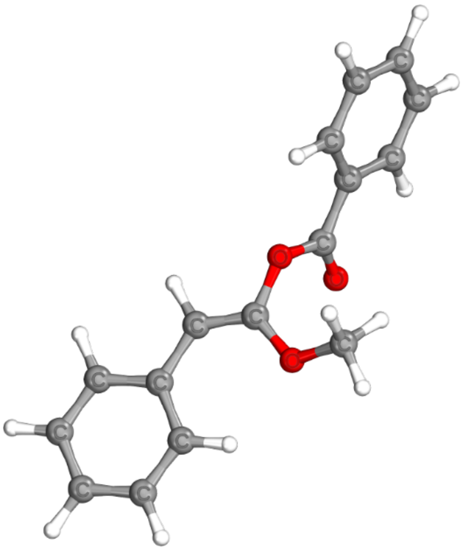

O	-1.15987085013771	0.54656339377237	1.70541509917177
O	-0.71626703792244	-0.25173179874098	-0.34857648577770
H	1.09276743300624	1.32050515201798	-1.01425385170132
H	-4.98114275065932	-0.46718176853991	-2.48307007237241
H	-6.69051685344131	0.39652091837486	-0.89509537327870
H	-2.58991193505883	-0.56024079790514	-1.80659381140574
H	-6.01221386179553	1.16863158310609	1.37255991650936
H	-3.60842458083799	1.07150804458692	2.04675487000050
O	0.89414570014452	-1.49530819780891	0.69765870232082
C	-0.03038675791849	-2.57022584340809	0.74805237935011
H	-0.31111413595951	-2.91028324254933	-0.26062130063370
H	-0.93539748998591	-2.30357734425839	1.31595917837289
H	0.48327770338694	-3.38438984664905	1.27357881453541

Cartesian coordinates of the PBE0-D3/def2-SVP+SMD(THF) optimized geometry of alkyne **27**
$E^{\omega B97M\text{-}V}$ = −442.37011773 hartree
Neutral, Singlet

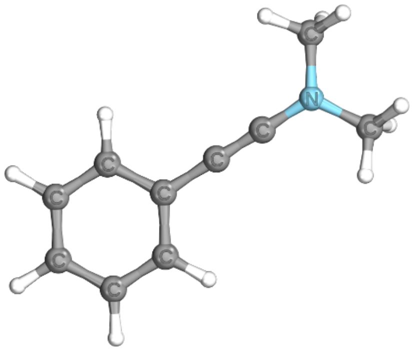

C	-3.75825554716450	1.23462499808029	-0.10696889905765
N	-3.03675078109689	0.00042414629126	0.12797058598859
C	-3.75685278865871	-1.23520381305026	-0.10379245469424
C	-1.71248310788528	0.00100314675860	0.07622269106217
C	-0.48825687306708	-0.00117427150247	0.06298998018700
C	0.93338127857135	-0.00090549078704	0.02845405015163
C	1.65674176584487	-1.21200299758861	0.01152117780103
C	3.04751820688660	-1.20506564369783	-0.02517820151942
C	3.75253757017247	-0.00021699387997	-0.04501177601772
C	3.04685139948644	1.20428744554290	-0.02769132616298
C	1.65607807541426	1.21055846198913	0.00896809912718
H	-3.16220368440230	2.08743180042593	0.24369009167008
H	-4.70676675701273	1.21743575263558	0.45147487833938
H	-3.98809092517299	1.38060955652181	-1.17895578583601
H	-4.70582647500932	-1.21713812265304	0.45382761122736
H	-3.16027395243393	-2.08634526412506	0.25000279143054
H	-3.98565259606934	-1.38467465051212	-1.17552015481521
H	1.11285041346538	-2.15928240798679	0.02727425318363
H	3.58823359241955	-2.15527432326698	-0.03809320743344
H	4.84487142889653	0.00005772553730	-0.07346571687261
H	3.58704061760605	2.15476711454500	-0.04260258650774
H	1.11166802870955	2.15756844612240	0.02270220004843

Cartesian coordinates of the PBE0-D3/def2-SVP+SMD(THF) optimized geometry of alkyne **TS1^{D3}**

$E^{\omega B97M\text{-}V}$ = −863.25062584 hartree

Neutral, Singlet

C	-0.29048968803896	-1.70690336428118	0.17631074312558
C	-1.22574807675532	-0.90752168562607	-0.22558247962170
C	-2.54339157073577	-1.23549111643341	-0.77339391401085
C	-3.38201199166378	-0.18224214243278	-1.16661413775815
C	-4.64257939080352	-0.44676402341512	-1.69790499914658
C	-5.07874492483707	-1.76333099935447	-1.84149477462672
C	-4.24914989427666	-2.81841931180192	-1.45177861859110
C	-2.99051623159029	-2.55905194493924	-0.92151425996102
H	-3.03478080343580	0.84759483710346	-1.05330510251795
H	-5.28742441729887	0.38146206492993	-2.00183635212252
H	-6.06747708763940	-1.97073479862440	-2.25844528858655
H	-4.58829463476777	-3.85132742954048	-1.56340594114823
H	-2.34373738067907	-3.38689594296709	-0.61810813235112
C	2.42753130961620	4.93135683587504	0.29292150644684
C	3.74671024739600	4.70480203718105	0.69105135971535
C	1.53360641097235	3.86515787778575	0.20312887958603
C	4.16697680757302	3.40891453206563	0.99851381122318
C	1.94674493893609	2.56444076024485	0.50979892292501
C	3.26970866355512	2.34600948683559	0.90730784817213
C	0.97701729930957	1.39735030271301	0.41122827599995
O	1.41121622879271	0.26220149173290	0.69018245681628
O	-0.19729447564579	1.67881936524601	0.04879494455102
H	-0.89496498432092	0.19317422742418	-0.11538182086320
H	2.09655636572620	5.94535489873304	0.05158484023531
H	4.44910756456081	5.53986759400792	0.76165586158276
H	0.49699667496034	4.01601599897788	-0.10669774716671
H	5.19976687717582	3.22950633705382	1.31032950463924
H	3.57229197420457	1.32254029646504	1.14069443712542
N	0.62750477902145	-2.47578721978828	0.56897654281720
C	1.72186306761737	-2.86200553927130	-0.31822610292153
C	0.76794132087719	-2.83623082613791	1.97754584160371
H	-0.13165319219525	-2.53368459319114	2.52455984566299
H	1.64522103545182	-2.31337112092200	2.38433635827621
H	0.91229669052026	-3.92255016386904	2.05705844795120
H	1.47360641011893	-2.58155597959241	-1.34752139163876
H	1.87511612478652	-3.94768343372125	-0.24673528879658
H	2.63047795351188	-2.33301730846563	0.00196592337385

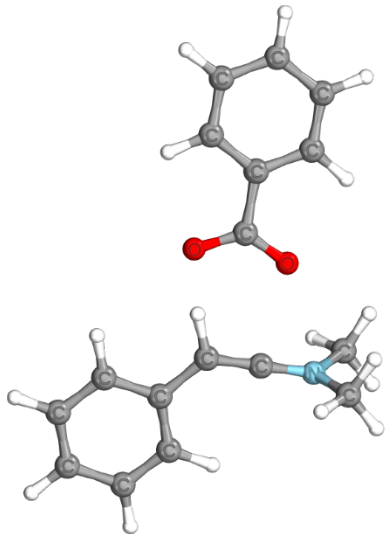

Cartesian coordinates of the PBE0-D3/def2-SVP+SMD(THF) optimized geometry of adduct **28**

$E^{\omega B97M\text{-}V}$ = −863.29793845 hartree

Neutral, Singlet

C	0.55203033235577	0.13615951766006	-0.11210503994783
C	1.48405818997608	-0.81672203615828	0.13001259295204
C	2.93644056784703	-0.70480363175015	0.05004647566756
C	3.72818302897157	-1.69034021432401	0.67250175611029
C	5.11773831757798	-1.65832958018368	0.59612069491760
C	5.76123315349073	-0.64330526053577	-0.11345539421547
C	4.99260540074067	0.33199463858917	-0.75183738049436
C	3.60332713940850	0.30646203417304	-0.67156058830689
H	3.23407589825673	-2.49348080931454	1.22666691937541
H	5.70409559443430	-2.43491573080718	1.09483005717398
H	6.85209002456529	-0.61548417509677	-0.17405785518115
H	5.48203154176687	1.12427753846181	-1.32502131356699
H	3.02031564711870	1.06666989352455	-1.19436842540749
C	-5.41937529575409	-0.16523378127533	0.73711152412882
C	-5.69857796791004	-0.78175118167099	-0.48437006896954
C	-4.10309347975735	0.11646744334034	1.08692989603743

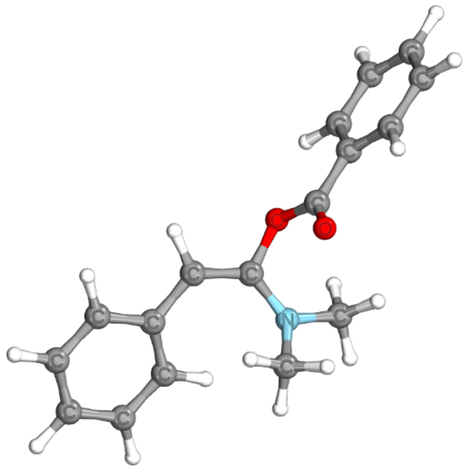

C	-4.66066813206281	-1.11627495626066	-1.35537652475596
C	-3.05912761997153	-0.21755912428838	0.21503299098584
C	-3.34132798136247	-0.83635752144219	-1.00961942085807
C	-1.67521365122601	0.11100977335301	0.63272117581515
O	-0.75824282983578	-0.29819932559688	-0.27742429453528
O	-1.37510176347834	0.66495123528848	1.65804191410066
H	1.08646358990121	-1.79546602753020	0.40881389073295
H	-6.23286726544164	0.09656447176724	1.41789385647913
H	-6.73310795115334	-1.00286434264929	-0.75937931563917
H	-3.86356641285300	0.59912651457362	2.03675196121297
H	-4.88113500176749	-1.59858825490235	-2.31068179231368
H	-2.52578698046844	-1.09608365653181	-1.68618033987374
N	0.72230550004272	1.48393362555721	-0.24895455435542
C	-0.19252920643794	2.26128623097588	-1.04967559499042
C	1.51809806622363	2.22611034345861	0.69871070477670
H	1.98569867108459	1.54345235048101	1.41795698435512
H	0.87662635254953	2.92901125944428	1.25983045584016
H	2.31401574018669	2.80814903488826	0.20397430050540
H	-0.60682900507429	1.65564890312142	-1.86588057454168
H	0.34856396435364	3.10891743207846	-1.49877424332431
H	-1.02997754699768	2.67475678798198	-0.45556794508977

7.2 Cyclization of *N*-alkynylated sulfoximine 9 with isobutyryl chloride (16)—Cartesian coordinates

Cartesian coordinates of the PBE0-D3/def2-SVP+SMD(DCM) optimized geometry of *N*-alkynylated sulfoximine **9**
$E^{\omega B97M\text{-}V}$ = −1107.54065719 hartree
Neutral, Singlet

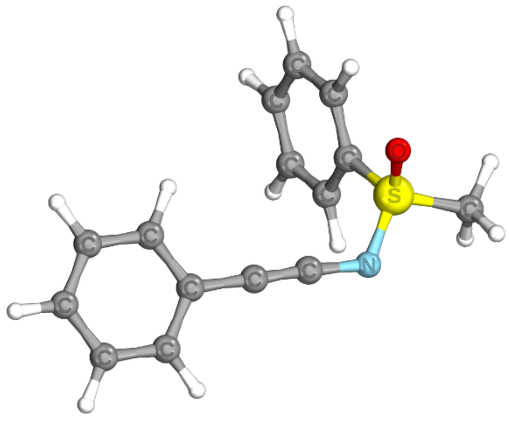

C	3.18199714191236	-2.33519154074571	-0.68046382705494
S	1.88195953572751	-1.43674764434207	0.09898694453025
C	2.20843548511690	0.29050351385532	-0.20050655116290
C	1.99772828409103	0.81655426033333	-1.47576100473063
C	2.24611401425537	2.17037827076022	-1.68340384801001
C	2.68726013458178	2.97186508531033	-0.62802986262222
C	2.88274424008717	2.42843659540741	0.64181967836090
C	2.64269366546738	1.07395551620106	0.86619661530034
N	0.61802323184183	-1.88033189640151	-0.73992721221968
C	-0.53345504836320	-1.28063161785042	-0.45323919848857
C	-1.62205505862450	-0.75758336356573	-0.25526207626517
C	-2.87836624632913	-0.13392099764380	-0.00226338554780
C	-2.99073900990826	0.89111270125578	0.95977369971585
C	-4.21736613789282	1.50055414624004	1.20581401491146
C	-5.35772503219963	1.10647457829251	0.50312236566556
C	-5.25910764928441	0.09306060052890	-0.45201089050013
C	-4.03692795484175	-0.52287616582523	-0.70521636991972
O	1.91043066763142	-1.65464571606712	1.55180002424665
H	2.94707145323533	-3.39806333339760	-0.52734849204104
H	4.11516558264510	-2.06098621845422	-0.17138181820289
H	3.21492203234965	-2.08763272326769	-1.74886939258495
H	1.63753008282754	0.18086571278523	-2.28813198704737
H	2.08712306493069	2.60360961660084	-2.67349063720761
H	2.87594844925926	4.03472974147535	-0.79789299622778
H	3.22166883247289	3.06138383059320	1.46516922535060
H	2.78048748923787	0.62453464404761	1.85150444648545
H	-2.10082646218323	1.20290230098116	1.51154305545331
H	-4.28327457949069	2.29315460489003	1.95598288904648
H	-6.31934817391937	1.58731615694555	0.69904753508020
H	-6.14594708828696	-0.22290599619229	-1.00787368346056

H	-3.96586863954714	-1.31540080085052	-1.45372996665310

Cartesian coordinates of the PBE0-D3/def2-SVP+SMD(DCM) optimized geometry of isobutyryl chloride (**16**)
$E^{\omega B97M\text{-}V}$ = −692.09418963 hartree
Neutral, Singlet

O	0.14849011629620	1.63616482007754	0.43062183656724
C	0.16254534974943	0.51718498467898	0.04665500344228
Cl	1.75081157765652	-0.34154657711117	-0.10836317502177
C	-1.00294424620366	-0.34854872517777	-0.35190293598952
C	-1.16475170615514	-1.50666796212689	0.63519335758401
C	-2.26289667243034	0.49422840531821	-0.47243778056860
H	-0.73469711114536	-0.77026443683887	-1.33579260916105
H	-1.38637275109972	-1.13111044267854	1.64641539251062
H	-0.26093877461345	-2.12987319675372	0.68379986308655
H	-2.00299670021543	-2.14230508921773	0.31452303796790
H	-3.09566456482027	-0.13354126875542	-0.82169386742100
H	-2.13143687328032	1.31850436316133	-1.18863012413994
H	-2.54461633093844	0.92685312842405	0.49955201714328

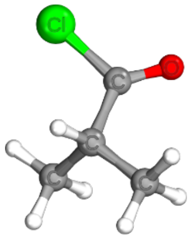

Cartesian coordinates of the PBE0-D3/def2-SVP+SMD(DCM) optimized geometry of triethylamine (**18**)
$E^{\omega B97M\text{-}V}$ = −292.37738381 hartree
Neutral, Singlet

C	-0.40601892531376	-2.60920137737182	0.15228274637766
C	-0.93146207735608	-1.20060686144432	-0.06904223308778
N	0.00199810473766	-0.16702413150658	0.34302867635242
C	1.10248345501263	0.00005182939545	-0.58839376953589
C	2.24611453843241	0.83665031093309	-0.04477212039541
C	-0.64388121583413	1.06903429907917	0.75257751260997
C	-1.34337103193775	1.87305532970621	-0.33973757856720
H	-0.12924776110595	-2.75689749699996	1.20816465488317
H	0.48141995623863	-2.82634655802183	-0.46243921545247
H	-1.17427953714003	-3.35291666929397	-0.11179310650361
H	-1.23910763943200	-1.08714079383438	-1.13275953253333
H	-1.85129552286441	-1.07268921661989	0.52466021169735
H	1.48965450060302	-1.00302552047942	-0.82587276380936
H	0.76407896021536	0.42483404779920	-1.56101660348377
H	2.57944003839834	0.45062035096323	0.93200289682180
H	1.97164418162794	1.89490850951977	0.08509418682142
H	3.10218159510508	0.80498040559760	-0.73621579575577
H	0.10622965818474	1.70895305146200	1.24559007674269
H	-1.37625657569459	0.81134714851108	1.53632948291726
H	-0.64055504160264	2.21894566335055	-1.11433637292324
H	-1.81968878914468	2.76731147065203	0.09253348282340
H	-2.13200052262979	1.28719608190275	-0.83853238199934

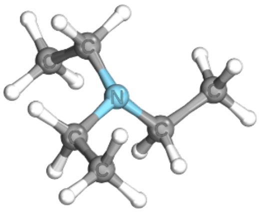

Cartesian coordinates of the PBE0-D3/def2-SVP+SMD(DCM) optimized geometry of structure **TS4**
$E^{\omega B97M\text{-}V}$ = −984.45017893 hartree
Neutral, Singlet

Cl	-1.81457197750000	-1.76122654183198	-1.00481998294990
O	-2.55026764498010	-0.29105810991792	1.07106877149366
C	-1.42110505352263	2.21508410910121	0.24150240428403
C	-0.95299287745377	1.05321285218375	-1.96938422576369
C	-1.17001521338957	0.90295355316302	-0.48033366294612
C	-1.94041314231687	-0.16363527043577	0.04519188931832
H	-0.62205026004897	2.93955249277074	0.01533975499954
H	-2.37554474648265	2.68539143611530	-0.06352184558931
H	-1.45794757680055	2.08555908497354	1.33338745482786
H	-1.83350956201837	1.49996042457806	-2.46998881714454
H	-0.10378808035587	1.72684032819919	-2.17006717619632
H	-0.74641168225052	0.09405626576272	-2.46123976773607

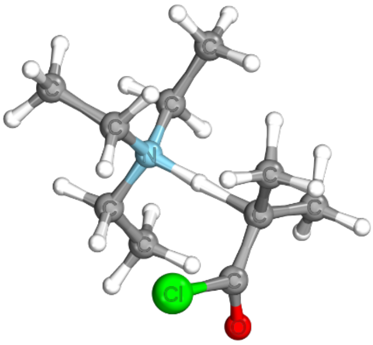

H	0.15585582796937	0.43608266158272	0.02773985218810
C	0.35580975082879	-0.33159482190984	2.70296336149352
C	1.04613987370558	-0.91617035133222	1.48864894355426
N	1.29784369243391	0.04475917218068	0.39159098183247
C	2.08723042729481	1.21102979954547	0.83637391502809
C	2.42553916661309	2.19252967868565	-0.26461425533276
C	1.83894015768351	-0.63895204201544	-0.80493846700467
C	3.23342516037545	-1.21458664859475	-0.66240244841737
H	0.98466846128073	0.38824776923365	3.24611441118573
H	0.12868562401481	-1.15396274966567	3.39778797746111
H	-0.59913709990341	0.14651935803552	2.44081863257842
H	2.00533208862952	-1.37090972851590	1.78514567094918
H	0.42335736174167	-1.71368112413459	1.05629799870436
H	1.48969567995111	1.71862273636811	1.60526381075887
H	3.00669311596985	0.85068925417060	1.32952566300335
H	1.52649251918293	2.52104049732080	-0.80711294462483
H	3.14035667286818	1.78431426987698	-0.99373597795033
H	2.88741062782013	3.08344255325183	0.18578869702744
H	1.80992145681372	0.08407370512543	-1.63064588588749
H	1.11365587289651	-1.42677673364070	-1.05598276518326
H	3.98182009215246	-0.44157289607985	-0.43103408748723
H	3.52367731775720	-1.67816884508188	-1.61716234625930
H	3.29147824433993	-1.99432928456848	0.11103764498486

Cartesian coordinates of the PBE0-D3/def2-SVP+SMD(DCM) optimized geometry of ion pair intermediate **19**
$E^{\omega B97M\text{-}V}$ = −984.46463448 hartree
Neutral, Singlet

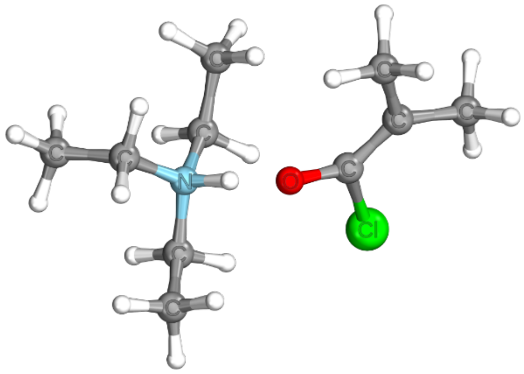

Cl	-1.10834603017183	-0.58177580144419	1.99377799215129
O	-0.71455206136128	-0.97444600121677	-0.58997077591665
C	-3.21629869758916	0.19788196594868	-1.52630945312878
C	-3.77678639683756	0.59194283202169	0.92640363912856
C	-2.80364255170582	0.07616190514718	-0.08511930002665
C	-1.59720989130503	-0.49293563520364	0.16697482756040
H	-2.44955348224945	-0.21056396689097	-2.19935792959770
H	-4.16398383068078	-0.33650491947282	-1.72803094846783
H	-3.39407556976280	1.25228591794648	-1.81145001419523
H	-4.00393928331397	1.66169539544541	0.75767050174835
H	-4.74725772180617	0.06400949607311	0.86198618911778
H	-3.40414994371420	0.48588719994052	1.95321713687244
H	0.63229525226341	-0.39646954320178	-0.43156343287068
C	2.33792360389309	-2.25485999904635	0.41736679194037
C	2.31263397711435	-0.77646481953593	0.73063489719309
N	1.63891169439854	0.03271225156484	-0.31912270313780
C	1.43487738202407	1.42709947089683	0.15748383020465
C	0.64788437366770	2.28895299476718	-0.80369009120843
C	2.26000592586041	-0.07138134692310	-1.66522351698404
C	3.66258223274693	0.48288684342555	-1.76516162969533
H	3.00546397505335	-2.50478115928271	-0.42001904052095
H	1.32453156126582	-2.61661576685995	0.18657838996939
H	2.70021715854378	-2.79883420646928	1.30181075948696
H	1.73292702816060	-0.60243277843922	1.64731699015901
H	3.32254426577210	-0.36965143051326	0.88898864368946
H	0.89052566563575	1.33032252046372	1.10749702321632
H	2.42021999204384	1.86349998807813	0.38036579030510
H	-0.30039444085848	1.80785144487083	-1.08939234310533
H	1.20996153087274	2.53272562308322	-1.71694169285788
H	0.40363281831561	3.23774936400429	-0.30404531084421
H	1.58300977387116	0.44114476913701	-2.36160316178949
H	2.23696716556566	-1.13457447424581	-1.93886009435614
H	3.70516258491199	1.55687274782122	-1.53060043853274

H	4.01763159440538	0.35683110532897	-2.79847877747842
H	4.36912044947022	-0.04512410761914	-1.10769948522887

Cartesian coordinates of the PBE0-D3/def2-SVP+SMD(DCM) optimized geometry of structure **TS5**
$E^{\omega B97M\text{-}V}$ = −984.45884570 hartree
Neutral, Singlet

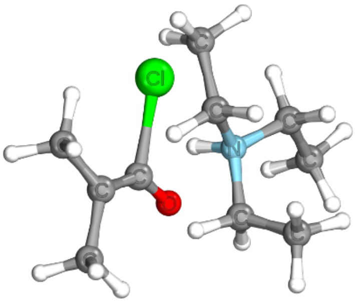

Cl	3.00790066624453	0.00622509458782	0.06907152870980
O	1.96723279751223	0.72333826995952	-2.38160852057126
C	0.18143250213884	2.91183824863785	-1.39754372261779
C	1.29774106461115	2.50577188523652	0.85617032177638
C	1.12725924210107	2.07900484527892	-0.56778094383857
C	1.84576840884465	1.14765574141555	-1.25862314990252
H	0.53792433981336	3.95436400077939	-1.51094663635757
H	0.04596587043518	2.49855837812223	-2.40754931780960
H	-0.81523595604425	2.97969030619277	-0.92365833655410
H	0.35060180787185	2.44199473271636	1.42481149097595
H	2.05291498816525	1.90193404710468	1.37334889985687
H	1.61354297888137	3.56540765227929	0.91778307494672
H	0.43861757391927	-0.02713524251600	0.08130482880879
C	0.49546292207707	-0.88344102261448	2.64660509961677
C	-0.66256979737125	-0.57675542456982	1.72503850340722
N	-0.33171962682416	-0.70628425762246	0.27341256999425
C	0.18257326640058	-2.06475706370660	-0.07725035995187
C	0.48111603598632	-2.23946615815550	-1.54758517045673
C	-1.45577915301611	-0.23202555031353	-0.59152576726216
C	-2.68167389572251	-1.11422800039216	-0.57920215888924
H	1.40687513330087	-0.34905579504176	2.34042745731811
H	0.72200422923895	-1.95798811783852	2.69437891519642
H	0.22580790838112	-0.55737422129299	3.66165380935114
H	-1.51615036981610	-1.23897718873215	1.92404355894321
H	-0.99959171866539	0.46056393236203	1.85719295155405
H	1.10374078685255	-2.18861759470489	0.50403763012835
H	-0.55717600342951	-2.79315486343538	0.28529140795573
H	1.13067880125174	-1.43401486453990	-1.92376129230022
H	-0.42827872918145	-2.28102022087240	-2.16438286342095
H	1.01225749825641	-3.19368654448590	-1.67834560041322
H	-1.04467377599728	-0.13388897782721	-1.60432079956017
H	-1.69234592526935	0.78154086664429	-0.24078952010296
H	-2.47443512572841	-2.12796063396037	-0.95144550149172
H	-3.43140724334174	-0.66547811065847	-1.24706333180667
H	-3.13638150187685	-1.19257814803675	0.41881094476755

Cartesian coordinates of the PBE0-D3/def2-SVP+SMD(DCM) optimized geometry of structure **20**
$E^{\omega B97M\text{-}V}$ = −753.23564795 hartree
Neutral, Singlet

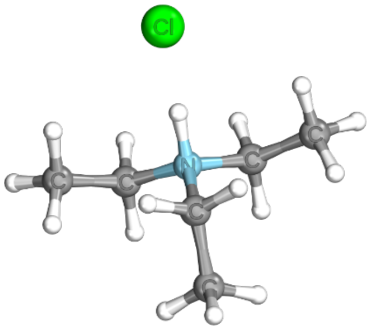

C	0.49840833200793	2.17161823420400	1.21281304797647
C	1.13901746531419	0.81107183038459	1.05635463266908
N	0.49838268022320	-0.02225216628374	0.00651147678118
C	0.43098201507214	0.61423023107047	-1.33287022131768
C	1.77147118918753	0.87904758351908	-1.97702222399991
C	1.06375850420338	-1.39625077220669	0.01092142372903
C	0.33375082548323	-2.34538520325664	-0.91240136987904
H	0.70765006151696	2.84065041019165	0.36555360694998
H	0.89713763125633	2.65024490472268	2.11907254036372
H	-0.59142424865369	2.06825612284110	1.32772189757379
H	2.21463135779565	0.88328177001518	0.83481414791979
H	1.03312552243520	0.23787843933950	1.98854310294416
H	-0.18879068021349	-0.04223309023804	-1.95737143450473
H	-0.14114335384509	1.54258694495143	-1.20658040937812
H	1.60607592696715	1.34764007502883	-2.95823675220798

H	2.34348024687756	-0.04567138047349	-2.14546259915260
H	2.39113156241971	1.56752397417004	-1.38320978149662
H	0.97727696429480	-1.74369395260245	1.05050033375546
H	2.13662892653883	-1.33129570616579	-0.22561965367460
H	0.51432707717920	-2.12810055605430	-1.97516951943845
H	-0.74896010887986	-2.30767805474695	-0.71706538026287
H	0.68483108747156	-3.36961914319127	-0.71979897426786
H	-0.54990009901861	-0.13094676787592	0.31322539543074
Cl	-2.30969613413380	-0.29620034064326	0.78466375538706

Cartesian coordinates of the PBE0-D3/def2-SVP+SMD(DCM) optimized geometry of ketene **21**
$E^{\omega B97M\text{-}V}$ = −231.23844340 hartree
Neutral, Singlet

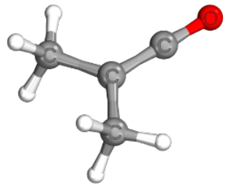

O	2.03777076700837	0.00002557261472	0.00013339138952
C	0.86790182771552	0.00001398898581	0.00007311636626
C	-0.44918119286439	0.00000131052162	0.00001852880980
C	-1.20762635349388	1.30104898590365	-0.00001329188841
C	-1.20760206611547	-1.30106051006817	-0.00003696945679
H	-1.85803175584856	1.37054359752913	0.88888316864027
H	-1.85784003466154	1.37062947676569	-0.88904382912760
H	-0.54084078770473	2.17514106090961	0.00010069994730
H	-1.85784802973645	-1.37061316795521	-0.88904591993131
H	-1.85797208550916	-1.37060747802237	0.88888139912583
H	-0.54080005778973	-2.17514007278450	0.00001172322511

Cartesian coordinates of the PBE0-D3/def2-SVP+SMD(DCM) optimized geometry of structure **TS6**
$E^{\omega B97M\text{-}V}$ = −1338.76289288 hartree
Neutral, Singlet

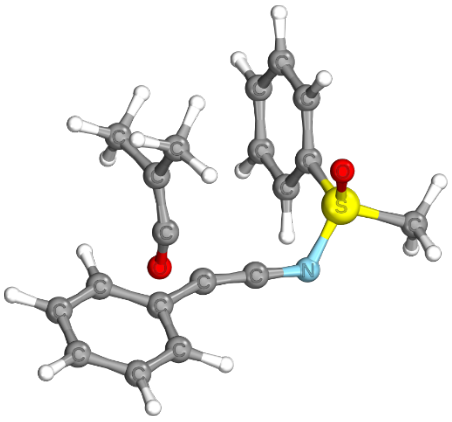

C	-3.16758059839935	1.21107837698317	2.42216089949196
S	-1.99571849590862	0.28545449667614	1.48953008596741
C	-2.59889171918237	0.25547618183431	-0.18123765371923
C	-3.22392824887957	-0.90959562018785	-0.62151775706153
C	-3.69607066136877	-0.95057013591552	-1.93164716295861
C	-3.53725195281082	0.15559247253512	-2.76711717597792
C	-2.90453607815534	1.31122795309026	-2.30458969990218
C	-2.42644746087226	1.37214035358274	-0.99909738358948
N	-0.71464174535772	1.24464590444652	1.52467231182527
C	0.43634265413939	0.75914026899761	1.15563204174422
C	1.52908042277017	0.39084974141677	0.68240594715465
C	2.91389959405569	0.01710622121671	0.82348838410632
C	3.65033102989235	-0.53283515028551	-0.23950647515655
C	4.98807917268060	-0.88133001092595	-0.06820992212798
C	5.61721250387660	-0.68713056228636	1.16132257201803
C	4.89601749291987	-0.14049780234703	2.22533157832403
C	3.55927622317424	0.20675962148544	2.06228612552402
O	-1.88856957910278	-1.08964264500553	1.98845655997780
H	-4.11868036149032	0.66416288491655	2.37986310010545
H	-3.25968906095158	2.21565796922579	1.98960070604253
H	-2.77663851986101	1.25332388323471	3.44904327864759
H	-3.32439071283684	-1.76770160101694	0.04584053014336
H	-4.18451000777891	-1.85488280554788	-2.30140385949232
H	-3.90412598606505	0.11441316156206	-3.79549741071024
H	-2.77022514632750	2.16824029921183	-2.96809702712229
H	-1.90043647995948	2.25618235319652	-0.63360163000217
H	3.15797972298597	-0.68505444911876	-1.20215909101920
H	5.54438521175867	-1.30840890087264	-0.90658261139940
H	6.66717849154650	-0.96095036078269	1.29199029933045
H	5.37973303915554	0.01591450970721	3.19308565782474
H	2.99632012114042	0.63318119575834	2.89590181601187
O	0.93670924579041	1.63945786563424	-1.65558323977063

C	0.81399966048015	0.50112418843783	-1.31126012193356
C	0.47679567903170	-0.76010096914937	-1.65211158955252
C	0.21616937224206	-0.96504406425227	-3.12668052356883
C	0.40578824213479	-1.98461283064927	-0.79811544905126
H	0.96639415498964	-1.63909968310244	-3.58116873527562
H	0.23216302808232	-0.02008247706747	-3.68836372754989
H	-0.76956109964287	-1.43740186861180	-3.28741070497713
H	1.21780239137501	-2.69848392043084	-1.03452894372255
H	-0.54030242651780	-2.52385341766111	-0.98480115629866
H	0.45230991404681	-1.76652255583264	0.27496691630007

Cartesian coordinates of the PBE0-D3/def2-SVP+SMD(DCM) optimized geometry of structure **22**

$E^{\omega B97M\text{-}V}$ = −1338.77550120 hartree

Neutral, Singlet

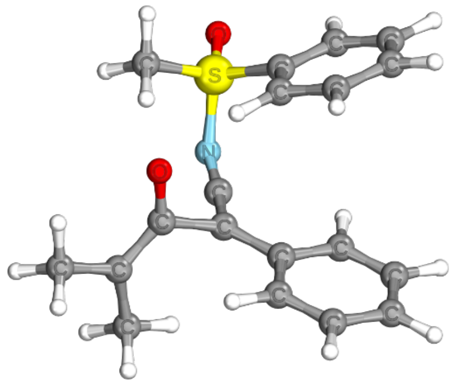

C	2.18680247268187	2.34523124355342	0.56565379875889
S	2.35420192859223	1.14613495541168	-0.70388803360111
C	2.30463616934569	-0.45113679630118	0.02916294859881
C	1.45376626357135	-0.71838365457766	1.10711140343929
C	1.39615762694705	-2.03275428965195	1.56658045317327
C	2.16083537709830	-3.03095750077844	0.96285330291282
C	3.00307901797096	-2.73311328598341	-0.11099793494356
C	3.08001052536384	-1.43200057499547	-0.59492296436829
N	0.94223501882703	1.35306148312050	-1.64535683774460
C	-0.10618136252134	0.93083238915579	-1.10888983183227
C	-1.10134271205463	0.57635786878325	-0.34112344568646
C	-1.75825658768780	-0.73433638500103	-0.29505798219383
C	-2.85631316960987	-0.93188432393819	0.55239664930135
C	-3.47294528980993	-2.17975547888207	0.62491539729782
C	-3.00474357360187	-3.24133807766128	-0.14872914074539
C	-1.91158146885374	-3.05060228192246	-0.99900610048127
C	-1.29248004480145	-1.80859527153997	-1.07303370302550
O	3.52237209145768	1.32931258265633	-1.55406626985995
H	3.07632975368929	2.26207783459395	1.20695971504125
H	1.23442952310581	2.14713495713618	1.11413927749414
H	2.15578600928535	3.31633352844825	0.04946480170250
H	0.81712793908212	0.06042639744953	1.55214613468850
H	0.73605583348650	-2.27555189211798	2.40228116588778
H	2.10090791941179	-4.05707937818259	1.33355983542059
H	3.60374279048680	-3.51795569813827	-0.57561834241880
H	3.73046959882990	-1.17505548739140	-1.43347934874416
H	-3.21980619928901	-0.09547033236418	1.15435016189026
H	-4.32752058982496	-2.32234221068632	1.29111689591214
H	-3.48967089397499	-4.21905869427768	-0.09084841622334
H	-1.53806350985750	-3.87874732378436	-1.60661326127003
H	-0.43177028017971	-1.66603979244387	-1.73268440491694
O	-0.54359678717691	1.58990128549626	1.72533064464245
C	-1.34482629302107	1.67790451586669	0.73208457381143
C	-2.35509005191342	2.57579858877967	0.50449801921732
C	-2.66694444028715	3.59209666684831	1.55987037549210
C	-3.23900711135220	2.56251996787376	-0.70315779986662
H	-1.94060328366434	3.52803906955903	2.38289008007284
H	-3.67958551601613	3.45570972836463	1.98641554841562
H	-2.64445464349620	4.62256069349012	1.15714723931898
H	-2.95732548452124	1.78685352173190	-1.43043520266597
H	-3.21932318998973	3.53228660110426	-1.23648542950964
H	-4.29922425132834	2.38991840439622	-0.43372868709238

Cartesian coordinates of the PBE0-D3/def2-SVP+SMD(DCM) optimized geometry of structure **TS7**
$E^{\omega B97M\text{-}V}$ = −1338.76948179 hartree
Neutral, Singlet

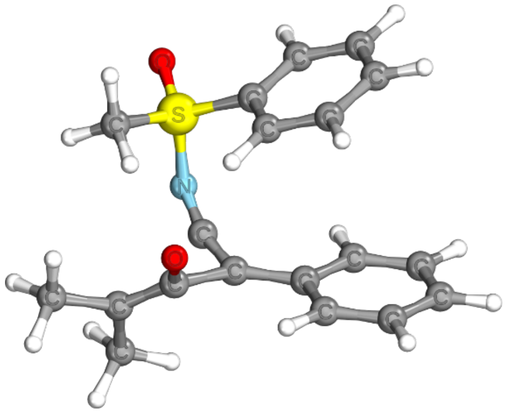

C	2.04282069255150	2.28720198938143	0.72586834142906
S	2.27816207748879	1.22624610540133	-0.66669956602521
C	2.30852588770906	-0.42640203597273	-0.04259353142606
C	1.49878661845907	-0.81736061799994	1.02863099484452
C	1.50759713853297	-2.16284822436507	1.39199465983280
C	2.29976069403639	-3.07868787576235	0.70055280895834
C	3.10133214660854	-2.66270727103686	-0.36487560085249
C	3.10990165229539	-1.32631443748330	-0.74941846604849
N	0.95322513974312	1.42550345081524	-1.61867506904507
C	-0.14165675420240	1.06089842799699	-1.05754518697774
C	-1.06492033866579	0.40056794206041	-0.39805557448100
C	-1.45372547556441	-1.00446238555498	-0.39744290994268
C	-0.99387527859556	-1.88092215648625	-1.39397419285174
C	-1.34726301946914	-3.22584182185683	-1.36342505353078
C	-2.16146867306952	-3.71513380804907	-0.33860615557904
C	-2.62234853806558	-2.84884866908951	0.65375590321112
C	-2.27571295911318	-1.49979502685985	0.62574530576933
O	3.49014575776768	1.54227603359343	-1.41128882048891
H	1.96430005009676	3.30268457636415	0.31241411009301
H	2.93270783209074	2.18407002596948	1.36244361784492
H	1.12150785442608	2.00026537039584	1.26147621058890
H	0.84338316790734	-0.11551159013409	1.55579688498851
H	0.87752603824324	-2.49426546317663	2.22034474912233
H	2.29357777795602	-4.13086461942289	0.99495859225069
H	3.72494909322391	-3.38233582684510	-0.89973075229866
H	3.73128247883977	-0.97925549162225	-1.57772830522731
H	-0.35155005631829	-1.49902466181126	-2.19207106046331
H	-0.98344401158582	-3.89971522401948	-2.14327617406158
H	-2.43548580573107	-4.77283592724668	-0.31415145572024
H	-3.25717639733865	-3.22765878896180	1.45892239609547
H	-2.61036650417021	-0.82110236701912	1.41281188187789
O	-0.96853015189045	1.22109206523008	1.83275638142782
C	-1.30326944739861	1.47783164556644	0.64638360536724
C	-1.51797907948392	2.74877189440978	0.07953841904667
C	-1.28459100855899	3.94005993973900	0.95097842122568
C	-2.24905383555023	3.01886234244768	-1.20094798031302
H	-0.80510231235825	4.75607504171611	0.38192030952791
H	-0.65803884215753	3.68296198526062	1.81753918388409
H	-2.23221908785193	4.35765702671200	1.34293228678521
H	-3.18277414940794	3.57775064809722	-0.99783844380870
H	-2.52100151774201	2.10469393116743	-1.74570020900359
H	-1.65793885368688	3.65642384845138	-1.88372055602589

Cartesian coordinates of the PBE0-D3/def2-SVP+SMD(DCM) optimized geometry of product **17**

$E^{\omega B97M\text{-}V}$ = −1338.86766731 hartree

Neutral, Singlet

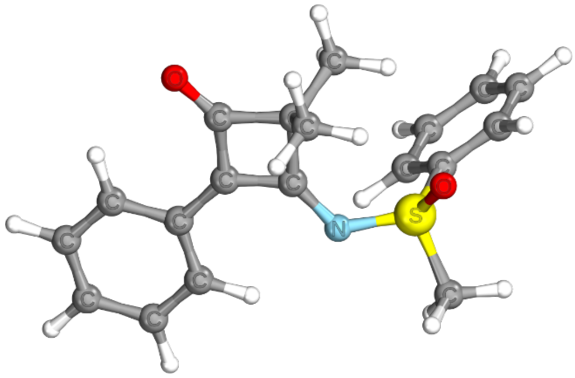

C	-1.45521662288424	1.30765593218442	-2.97707122104784
S	-0.35288759180494	1.49808780571086	-1.61289384495201
C	-1.30858909622039	2.30172623142558	-0.33880987001394
C	-2.12368497875873	1.54001720841076	0.49809782615082
C	-2.84972532457353	2.19486556577120	1.49009608092902
C	-2.75460320982228	3.58062455678826	1.62942617308390
C	-1.93350969633003	4.32408369454376	0.78051246175111
C	-1.19913463246543	3.68594919642612	-0.21649291735766
N	-0.10519258071194	0.00215395459577	-1.17210968864156
C	0.85559513962552	-0.32640860191906	-0.29035523200885
C	1.06913881856207	-1.51416829584426	0.39168426261389
C	0.39268892197690	-2.79623709694189	0.46429033478506
C	0.89318160512490	-3.80010900700059	1.31478597765572
C	0.25708810959422	-5.03528093282314	1.40301680031601
C	-0.88839437422962	-5.29460676063664	0.64733036073408
C	-1.39303768143987	-4.30557585100668	-0.19985068799474
C	-0.76179864620910	-3.06782553944103	-0.29366971217534
O	0.75446823703477	2.39120223950201	-1.99108129507233
H	-2.32673837965052	0.71972278147512	-2.66342300954987
H	-0.88455434476841	0.78539701104184	-3.75828257684420
H	-1.74358740582691	2.31513870200375	-3.30461394504993
H	-2.17813399714325	0.45528042385818	0.38312075699267
H	-3.48975358284989	1.61728408602199	2.16088193753848
H	-3.32485019588806	4.08665109728411	2.41220360937902
H	-1.85856443204156	5.40762277581049	0.89737846163509
H	-0.53908739050095	4.24608194356664	-0.88197785427240
H	1.78925412086681	-3.59048281214403	1.90384788188513
H	0.65877093589180	-5.80450552524657	2.06821224404767
H	-1.38636781291670	-6.26502218628904	0.71822072432745
H	-2.28893546271952	-4.50204585160531	-0.79518851567027
H	-1.15530821883211	-2.29606246760709	-0.95927588718028
O	3.06061881591976	-1.33586610158825	1.85899866032396
C	2.24587864022018	-0.95699873964823	1.04368359727571
C	2.08478565123128	0.39395986836708	0.29155678419129
C	3.21024225120755	0.67900628831944	-0.69297167801259
C	1.79788936661303	1.58365702217110	1.19547701777443
H	4.13241067914081	0.92987550459400	-0.14481899233397
H	2.95289648002215	1.52585745725170	-1.34584630616106
H	3.41875120480286	-0.19715413501335	-1.32695373542008
H	0.96589321850732	1.37867968358190	1.88708505571284
H	1.54797076622963	2.47954744263691	0.60600940234109
H	2.69138826351642	1.81821801291219	1.79589432431450

7.3 Cyclization of *N*-alkynylated sulfoximine 9 with isobutyryl chloride (16) (alternative mechanism 2)—Cartesian coordinates

Cartesian coordinates of the PBE0-D3/def2-SVP+SMD(DCM) optimized geometry of *N*-alkynylated sulfoximine **9**
$E^{\omega B97M\text{-}V}$ = −1107.54065719 hartree
Neutral, Singlet
(see section 7.2.)

Cartesian coordinates of the PBE0-D3/def2-SVP+SMD(DCM) optimized geometry of isobutyryl chloride (**16**)
$E^{\omega B97M\text{-}V}$ = −692.09418963 hartree
Neutral, Singlet
(see section 7.2)

Cartesian coordinates of the PBE0-D3/def2-SVP+SMD(DCM) optimized geometry of triethylamine (**18**)
$E^{\omega B97M\text{-}V}$ = −292.37738381 hartree
Neutral, Singlet
(see section 7.2)

Cartesian coordinates of the PBE0-D3/def2-SVP+SMD(DCM) optimized geometry of structure **TS8**
$E^{\omega B97M\text{-}V}$ = −1799.61116454 hartree
Neutral, Singlet

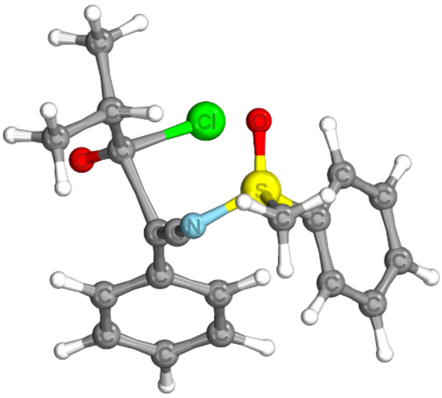

S	2.42291997395683	-0.89437219696175	-0.85395787801610
O	2.85655447152992	-1.59719287312980	0.35502561134768
N	0.97868046541248	-1.36381725059807	-1.46423218506724
C	-3.38976276703365	0.43348938561810	-0.48364088427936
C	-4.29111704060095	1.49590194223048	-0.53707555471841
C	-3.83636385744524	2.81215305602656	-0.45597467053736
C	-2.46915223826967	3.06420870849822	-0.32276142507115
C	-1.56277831554817	2.00895917330749	-0.27559648866233
C	-2.01480129106982	0.68190047871808	-0.36332626840184
C	-1.06739578919289	-0.41575301166689	-0.28953750169670
C	-0.05320207278389	-0.82633033388122	-0.91570500579492
C	2.53269358864109	1.28292565275122	0.74316311624606
C	2.43477228461989	2.64854747729003	1.00246961066269
C	2.19262015462233	3.54321007375008	-0.03899778145738
C	2.03822773682891	3.08612573126176	-1.35061587284654
C	2.12215449295567	1.72661655853704	-1.63081499342543
C	2.38199408260104	0.85240126890573	-0.57260619701653
C	3.50960947695324	-1.22260462393438	-2.19387630872357
H	-3.74510425246348	-0.59646218709452	-0.53844420901770
H	-5.35999652690615	1.29146667310829	-0.64094071242256
H	-4.54691020459670	3.64186647614277	-0.49432812769146
H	-2.10410926175582	4.09245436143517	-0.25414006163926
H	-0.49415484980762	2.20179145934348	-0.16295239832646
H	2.70584313360113	0.56136293548790	1.54254870159300
H	2.54315817785804	3.01057185003144	2.02706120485689
H	2.11673045641006	4.61245703789557	0.17244968536007
H	1.84044722315885	3.79205395238938	-2.15994022250371
H	1.97929043623470	1.36159976381448	-2.65047120382523
H	3.19184266426181	-0.65426954068609	-3.07778906332719
H	4.51976850192704	-0.94187984381833	-1.86726919018978
H	3.43521137059159	-2.30468843191967	-2.37475146839006
O	-2.56670864397519	-1.44400860094332	1.59888960360390
C	-1.38842573534933	-1.45235398121429	1.38577052133296
Cl	-0.33455381446426	-0.09764680605848	2.47902638631272
C	-0.51751253539175	-2.69977372431950	1.32329520644345
C	-0.45001241515958	-3.33448200956264	2.70904199004484
C	-1.09323245324303	-3.67232704462086	0.30110848246893
H	0.49519554457983	-2.39392517920354	1.02799915334230
H	-1.45221386406789	-3.63581398133468	3.05184742254100

H	-0.02903335031234	-2.63308525758641	3.44261974343632
H	0.18634883016557	-4.23217483485622	2.67797293196433
H	-0.45671926110162	-4.56796656317824	0.23687976150242
H	-1.15590271471538	-3.23049070652915	-0.70381730622238
H	-2.10489981165562	-3.99064503344527	0.59639384621104

Cartesian coordinates of the PBE0-D3/def2-SVP+SMD(DCM) optimized geometry of structure **29**
$E^{\omega B97M\text{-}V}$ = −1339.26511290 hartree
Monocation, Singlet

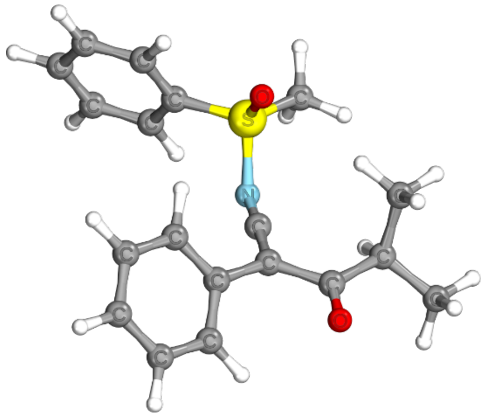

S	1.85653407783315	-0.65007943034622	0.08685161322182
O	1.60816232161677	-0.46084278092750	1.51037325099772
N	0.48606268965383	-0.71836131225066	-0.91681075833212
C	-3.65887716945806	1.28364082366931	-0.18762498770849
C	-4.13724013070518	2.58671296247785	-0.07644698126176
C	-3.32610977479096	3.59563287142333	0.44494356486484
C	-2.02798628578526	3.29896377824112	0.85993108838417
C	-1.54298974189515	1.99778530053650	0.75660033208784
C	-2.35504356078123	0.98236753074013	0.23115924267433
C	-1.82752078992487	-0.39755097528109	0.10154063397247
C	-0.62069710710904	-0.56611730168262	-0.39357034532587
C	3.26695661784746	1.62050369217200	0.27041521554689
C	4.00152785297630	2.67813340804169	-0.25856926920589
C	4.22193484266778	2.75919552229568	-1.63322439729416
C	3.71291315501287	1.78800623640379	-2.49958047807627
C	2.97560241084363	0.72100242183390	-2.00046799914829
C	2.77357107136793	0.66477030129496	-0.61828809750361
C	2.61831060389944	-2.18651169800815	-0.28425045739561
H	-4.29313428055771	0.50082340095061	-0.60405232618524
H	-5.15359782638919	2.81618075885693	-0.40576347503190
H	-3.70829445186055	4.61592906264549	0.52965203742806
H	-1.38944383196090	4.08265489357303	1.27461492377331
H	-0.53467726995696	1.76113416491782	1.10533749907829
H	3.08046445642558	1.53882439175934	1.34309404578060
H	4.40100994563169	3.44224356323494	0.41130888120911
H	4.79870313251651	3.59353747634073	-2.03907530006680
H	3.88755506748640	1.86313724560538	-3.57478025715403
H	2.56521517032508	-0.03584789870237	-2.67231328051160
H	2.76434081366365	-2.25650224047323	-1.37115942616898
H	3.57546774430000	-2.19239500486270	0.25710231144869
H	1.93853580998003	-2.96924952335841	0.08568061237540
O	-3.77647520564943	-1.44012100956621	0.90142263928881
C	-2.63514379747751	-1.60067040799854	0.54531483519277
C	-1.94291874200199	-2.94764550545345	0.55944411394773
C	-1.13773401676554	-3.08232379212867	1.85777715316923
C	-2.94823765315622	-4.07678854606536	0.40073634969594
H	-1.23666313949447	-2.96807799060109	-0.28902690518798
H	-1.80691846855235	-3.06262364021146	2.73196272070955
H	-0.39550479546424	-2.27927806450065	1.97981593441439
H	-0.59971863287236	-4.04222570520459	1.86106493278898
H	-3.51095790682625	-3.98757878294663	-0.54061966515523
H	-3.67146965376435	-4.07524660050676	1.22952535248647
H	-2.42952386583604	-5.04674733776656	0.39947022274883

Cartesian coordinates of the PBE0-D3/def2-SVP+SMD(DCM) optimized geometry of structure **TS9**
$E^{\omega B97M\text{-}V}$ = −2091.99825346 hartree
Neutral, Singlet

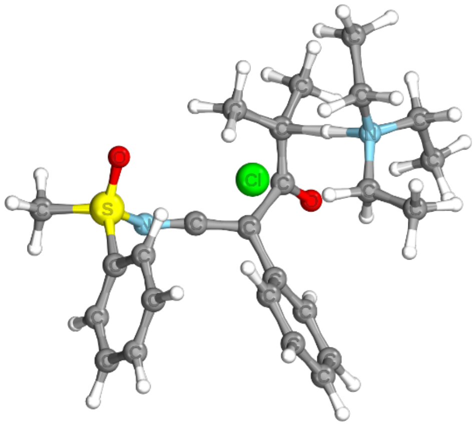

S	-2.63083111012558	-3.02544772834959	-0.18913217724491
O	-1.38496222355720	-3.67265724778982	-0.57501654984636
N	-2.56306583638298	-1.93313327415022	1.10212806765524
C	-1.71917907180896	2.46636494992108	1.27166544848845
C	-2.13490814996226	3.62084611997380	0.61054185686003
C	-2.29793914096743	3.62197050415020	-0.77628031716254
C	-2.03014611823040	2.46388461114024	-1.50696185187907
C	-1.60419518786635	1.30601572210885	-0.85834900316534
C	-1.45405702647818	1.30356354772829	0.53603654912318
C	-0.99243279929376	0.06295277759941	1.21578629432418
C	-1.73482633622087	-1.00134519392189	1.07953826083133
C	-2.52006451335453	-1.85441275937346	-2.59268098032651
C	-3.02854537287370	-1.06589347387473	-3.62404516250273
C	-4.32592788363069	-0.56442906803289	-3.55457077181860
C	-5.13543263188725	-0.83140649251836	-2.44523414232599
C	-4.65379048654949	-1.60641133460373	-1.39767335334333
C	-3.35422167694958	-2.11367448398281	-1.50762799248359
C	-3.79861577172220	-4.16714983577726	0.45147076701015
H	-1.58604432327058	2.45073558541394	2.35369541906313
H	-2.34098001847186	4.52682194067056	1.18647941292035
H	-2.62936536854079	4.52948539486066	-1.28760584361746
H	-2.13884773010253	2.46065412786757	-2.59489428194834
H	-1.31479845817727	0.42037759587618	-1.43400290915006
H	-1.47197783111685	-2.17807521846956	-2.61049233740158
H	-2.38239032595903	-0.82989242642526	-4.47229252167970
H	-4.71377502337776	0.05364671189418	-4.36795648822348
H	-6.14791242582212	-0.42578614667275	-2.39192713922132
H	-5.27805305107092	-1.79871750916368	-0.52302252459870
H	-4.70838663802652	-3.63682821675118	0.76118354189809
H	-3.99737247833634	-4.89783398923748	-0.34451273664258
H	-3.30623265548956	-4.63807042073985	1.31493026387146
O	0.18306779113624	0.98934855740299	3.00988844587296
C	0.21989900635859	0.08493487693720	2.17012697665623
Cl	0.65264303762524	-1.03152000041437	-2.54969092427798
C	1.28665636358638	-0.83989243707732	1.95944275393793
C	2.19271820361944	-1.02592089803795	3.16232455882535
C	1.04953240600693	-2.09916298521195	1.14944732756410
H	2.10531945675416	-0.01440616709083	1.10066440858349
H	1.75643912835536	-1.73700597224466	3.89002613178902
H	2.36075454514656	-0.07924109065064	3.69486660054429
H	3.17338427430011	-1.43291355618284	2.86883868681031
H	2.00690206222943	-2.62248159755370	0.99390487549022
H	0.64452163400262	-1.90143589397428	0.14396062470629
H	0.38650313373637	-2.82440647362110	1.65731264819727
H	1.29377290062579	0.81196800987011	-0.98430345071090
H	3.97227775771668	-1.80101498189666	0.80937783282813
H	1.22920855501890	1.98792631681249	0.34812578176582
H	2.20630184302658	2.03301057762450	2.64625833935542
C	1.89284520894806	1.52936123028647	-0.39772204422474
N	2.88548351002302	0.72180707144730	0.33908390431337
H	2.88208956973188	-0.65609211545251	-1.21144671749286
C	4.52929148843727	-1.17339890379188	0.09861947619606
C	3.04886450137720	2.49667365829477	2.10936885307281
H	2.65923864438278	3.35554627364821	1.54293965879918
C	3.63635349878876	-0.15634158484915	-0.58036114076020
H	1.61079180008314	3.13671622560368	-1.77869461407210
H	4.95195566968085	-1.83868560847035	-0.66939183295643

C	2.45217399790072	2.59303215570434	-1.32214972239904
C	3.77087490846484	1.50315380204934	1.22233861654412
H	5.37466914378092	-0.71443936420151	0.63224995423097
H	3.75713425613736	2.88952502236024	2.85423328948004
H	4.31396943977653	0.78103304681273	1.84716562344908
H	3.04274282812602	2.15952515246144	-2.14316815853109
H	4.24737454647121	0.47734822172028	-1.24725521686420
H	3.07731755022838	3.33225307413263	-0.79800129080293
H	4.52620500403929	2.01904158818251	0.60244294661655

Cartesian coordinates of the PBE0-D3/def2-SVP+SMD(DCM) optimized geometry of structure **20**
$E^{\omega B97M\text{-}V}$ = −753.23564795 hartree
Neutral, Singlet
(see section 7.2)

Cartesian coordinates of the PBE0-D3/def2-SVP+SMD(DCM) optimized geometry of structure **22**
$E^{\omega B97M\text{-}V}$ = −1338.77566574 hartree
Neutral, Singlet

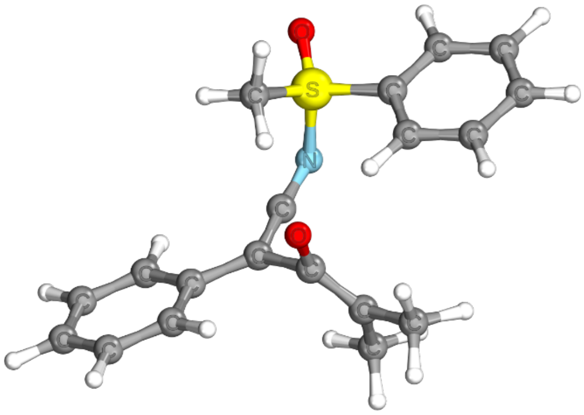

C	-1.03276657307717	-0.99836267196082	-1.86162006432122
S	-1.86322944923733	0.42342479916190	-1.22633236232447
C	-2.74140922074526	-0.06621806800951	0.21662878189686
C	-2.14060639336707	-0.91915305645433	1.14737865812653
C	-2.86679348750541	-1.22442280502640	2.29628192798332
C	-4.13720043115404	-0.68244475656350	2.49855957814368
C	-4.70827935628661	0.16946140826629	1.55132835419008
C	-4.00871547727277	0.49067051397717	0.39206561465345
N	-0.71974442249146	1.51628825601169	-0.63710534334309
C	0.43014243807546	1.05184955431382	-0.43966623076836
C	1.50604107375445	0.37177188443670	-0.15611309626982
C	2.74649369937862	0.24870712978445	-0.92363817179908
C	3.05201017960003	1.13825476143163	-1.96698166571554
C	4.24259985702385	1.00859523442675	-2.67491551391495
C	5.14390075124121	-0.00923754116685	-2.35407557653955
C	4.84530399807380	-0.89718587401874	-1.31980558429989
C	3.65659442359775	-0.77037868175729	-0.60536997215115
O	-2.73256555206943	1.07243766730741	-2.19811436688579
H	-1.82748996815730	-1.69905820986614	-2.15495977326277
H	-0.36458536450692	-1.41857255692850	-1.08198661418703
H	-0.46455141314944	-0.65300409176610	-2.73794358337025
H	-1.14128433906512	-1.35258013209734	0.98439187086048
H	-2.42946155551798	-1.89263782485874	3.04156302734522
H	-4.69191257876100	-0.92939646391258	3.40704064428128
H	-5.70440284080621	0.58686943446382	1.71333770739676
H	-4.43414208005465	1.15235210041658	-0.36525319276704
H	2.35412785046832	1.94357809355441	-2.21243295232920
H	4.47183842118485	1.71017521602948	-3.48102711582643
H	6.07945353242919	-0.10857202395041	-2.91021870064925
H	5.54474234546461	-1.69794026049750	-1.06654688221457
H	3.40823291520518	-1.47597415052063	0.18990660390453
O	0.82317387850866	-1.72031819230994	0.64188886427762
C	1.17171708332083	-0.55783681084441	1.03888876175855
C	1.21926098030485	-0.04801976538375	2.31267517748614
C	0.90417062842859	-0.95754975683270	3.46009649228434
C	1.65570719504520	1.34120456500178	2.65706121226662
H	1.77909700097935	-1.10946181178385	4.12112179693152
H	0.58065283823046	-1.94431317724647	3.09848799935400
H	0.10741934887210	-0.54131987946966	4.10499226178584
H	0.84949001022934	1.91647822387954	3.15132444354804
H	1.97547388982351	1.91854748963443	1.77773275578213
H	2.49954527858493	1.33175458742838	3.37304004778247

Cartesian coordinates of the PBE0-D3/def2-SVP+SMD(DCM) optimized geometry of structure **TS10**

$E^{\omega B97M\text{-}V}$ = −1338.76812081 hartree

Neutral, Singlet

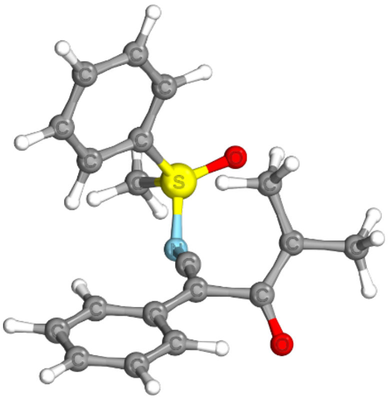

C	3.21950035735623	0.70038050985940	-2.62415175613300
S	2.29068721792670	-0.22169618708905	-1.45188325556134
C	2.37055803882614	0.63628891152281	0.09495286288253
C	1.71195678206120	1.85770850275393	0.24450956312128
C	1.77575686532191	2.49048802529635	1.48201011315558
C	2.48742387388747	1.90624533102288	2.53187681339183
C	3.14486128628116	0.68832462485619	2.35550126347049
C	3.09150080833706	0.03659586044010	1.12600136892272
N	0.78199213291734	-0.07935776830695	-2.07522339248851
C	-0.23969553813303	-0.32040204501524	-1.35025114253992
C	-1.41511033288186	-0.45252172221233	-0.80268871701470
C	-2.39779575647415	0.55095721303476	-0.38695895068585
C	-2.10848105834610	1.92444637944357	-0.44226869416852
C	-3.04144473664575	2.86144132527149	-0.00917727802002
C	-4.28066645983786	2.44386896345431	0.48172202744793
C	-4.57998166661686	1.08169297411335	0.52987784594932
C	-3.64866760201920	0.13943798321371	0.09954977216006
O	2.81481777968150	-1.58019534075941	-1.31310720256828
H	3.08789368817980	0.18615419517502	-3.58714078848641
H	4.26707893369226	0.67118669090563	-2.29500146962926
H	2.83461090239633	1.72808799041242	-2.66545876833073
H	1.15582717545518	2.30672812051922	-0.58116228261080
H	1.26164719073152	3.44306794761535	1.62657577063480
H	2.52876271645292	2.40794031634736	3.50158275247662
H	3.69977701986299	0.23707219174520	3.18081792131575
H	3.58751431341571	-0.92287779379262	0.96843142597832
H	-1.14144444161326	2.25611152220422	-0.82792490305994
H	-2.80035917050591	3.92663561192577	-0.05508026167323
H	-5.01299479158754	3.18036415086067	0.82206192659556
H	-5.55128350684134	0.74815518059557	0.90442509684290
H	-3.88535100510586	-0.92589207687064	0.10469912778977
O	-2.64986394052162	-2.38201068699714	-1.43798898738282
C	-1.65714159358817	-1.97017966113247	-0.80123222748185
C	-0.64680212435061	-2.73412441606748	-0.19625363047314
C	-0.75313838051076	-4.21973673955573	-0.34342293244439
C	0.31136467018353	-2.26548071804920	0.85525108303204
H	-1.37499731106678	-4.48127399248768	-1.21240389681859
H	-1.20907748356847	-4.70182430523512	0.54497664623562
H	0.24400938070261	-4.68099290316601	-0.45984327717711
H	0.24743693907935	-1.18746897444697	1.05686785906229
H	1.35986175420238	-2.49579539772562	0.59270661657546
H	0.11945707326384	-2.78754979367960	1.81422595770754

Cartesian coordinates of the PBE0-D3/def2-SVP+SMD(DCM) optimized geometry of structure product **17**

$E^{\omega B97M\text{-}V}$ = −1338.86766731 hartree

Neutral, Singlet

(see section 7.2)
